# Spatially resolved protein map of intact human cytomegalovirus virions

**DOI:** 10.1038/s41564-023-01433-8

**Published:** 2023-08-07

**Authors:** Boris Bogdanow, Iris Gruska, Lars Mühlberg, Jonas Protze, Svea Hohensee, Barbara Vetter, Jens B. Bosse, Martin Lehmann, Mohsen Sadeghi, Lüder Wiebusch, Fan Liu

**Affiliations:** 1 Research group ‘Structural Interactomics’, Leibniz Forschungsinstitut für Molekulare Pharmakologie (FMP), Berlin, Germany; 2 Labor für Pädiatrische Molekularbiologie, Department of Pediatric Oncology and Hematology, Charité - Universitätsmedizin Berlin, Berlin, Germany; 3 Research group ‘Structural Bioinformatics’, Leibniz Forschungsinstitut für Molekulare Pharmakologie (FMP), Berlin, Germany; 4 Cellular Imaging core facility, Leibniz Forschungsinstitut für Molekulare Pharmakologie (FMP), Berlin, Germany; 5 Centre for Structural Systems Biology, Hamburg, Germany; 6 Hannover Medical School, Institute of Virology, Hannover, Germany; 7 Cluster of Excellence RESIST (EXC 2155), Hannover Medical School, Hannover, Germany; 8 Leibniz-Institute of Virology (LIV), Hamburg, Germany; 9 Department of Mathematics and Computer Science, Freie Universität Berlin, Berlin, Germany; 10 Charité Universitätsmedizin Berlin, Berlin, Germany

**Keywords:** Herpes virus, Systems analysis

## Abstract

Herpesviruses assemble large enveloped particles that are difficult to characterize structurally due to their size, fragility and complex multilayered proteome with partially amorphous nature. Here we used crosslinking mass spectrometry and quantitative proteomics to derive a spatially resolved interactome map of intact human cytomegalovirus virions. This enabled the de novo allocation of 32 viral proteins into four spatially resolved virion layers, each organized by a dominant viral scaffold protein. The viral protein UL32 engages with all layers in an N-to-C-terminal radial orientation, bridging nucleocapsid to viral envelope. We observed the layer-specific incorporation of 82 host proteins, of which 39 are selectively recruited. We uncovered how UL32, by recruitment of PP-1 phosphatase, antagonizes binding to 14-3-3 proteins. This mechanism assures effective viral biogenesis, suggesting a perturbing role of UL32-14-3-3 interaction. Finally, we integrated these data into a coarse-grained model to provide global insights into the native configuration of virus and host protein interactions inside herpesvirions.

## Abstract (web summary)

Quantitative and spatial resolution on the protein interactome of intact human cytomegalovirus virions map host and virus proteins within the multilayered tegument.

## Main

The structured assembly of infectious particles, called virions, is fundamental for virus transmission among cells and organisms. Virions contain the viral nucleic acid genome enclosed in a capsid protein shell. A number of co-packaged proteins facilitate the infection process and the onset of viral gene expression. Herpesviruses, a family of double-stranded DNA viruses, assemble particularly large and complex particles, accommodating many different proteins that are delivered to the host cell upon infection.

The capacity of herpesvirions to incorporate a large set of proteins is enabled by their typical multilayered architecture^1^. The outer lipid envelope harbours various viral glycoproteins required for host cell receptor binding and membrane fusion^2^. The space between the envelope and the central icosahedral nucleocapsid is filled with a proteinaceous matrix, the tegument. While individual tegument proteins have been allocated to distinct inner- and outer sublayers on the basis of biochemical^3^ and microscopic data^4,5^, the details of tegument protein organization are not understood. Herpesvirus particles also incorporate numerous host proteins, but very few of these events have been functionally or mechanistically characterized^6,7^.

Recent cryogenic electron microscopy (cryoEM) studies of herpesvirions have revealed substructures of the nucleocapsid^8,9^, the portal^10,11^ and several glycoprotein complexes^2,12,13^. In addition, previous studies only provided insight into the overall protein composition of herpesvirions, identifying between 46 and 82 viral proteins^14–18^. However, a systematic characterization of the spatial coordination and interactions of virus and host proteins within virions is lacking.

Here we use crosslinking mass spectrometry (XL–MS) to build a virion-wide proximity map of 32 viral and 82 host proteins in intact extracellular virions of human cytomegalovirus (HCMV), the largest human herpesvirus. The data enable de novo allocation of host and virus proteins and their protein–protein interactions (PPIs) to virion layers, providing insights into the organization of the tegument sublayers. We find that the viral protein UL32 (also known as pp150) acts as a dominant scaffold, engages in PPIs across the particle and mediates the recruitment of host proteins, such as 14-3-3 proteins and protein phosphatase 1 (PP-1). PP-1 antagonizes 14-3-3 binding to UL32 and is required for the efficient start of viral gene expression and production of viral progeny. Thus, by charting the proteome organization within native herpesvirions, we provide a basis for the structural and functional understanding of crucial PPIs.

## A PPI network of the intact HCMV particle

XL–MS allows capturing protein contacts from native environments^19^ such as organelles^20–24^. We reasoned that XL–MS is also suitable for gaining global insights into PPI networks of large and structurally heterogeneous herpesviral particles.

**Fig. 1:**
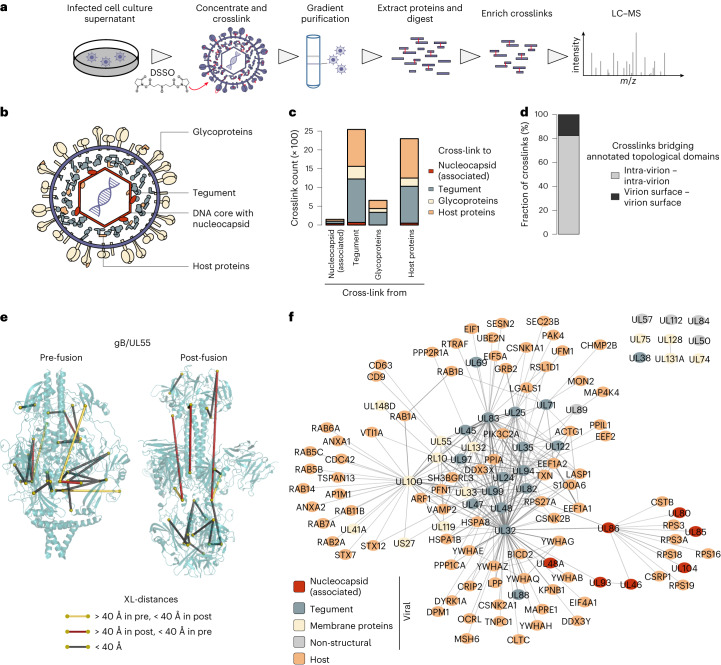
The spatial proteome of intact cytomegalovirus virions. **a**, Workflow for the XL–MS analysis of intact HCMV particles. The experiment was performed in *n* = 2 biological replicates. **b**, Schematic depiction of the HCMV virion layers. **c**, Number of crosslinks between viral proteins with known virion layer localization and between viral and host proteins. **d**, Proportion of crosslinks within annotated extra-virion and intra-virion domains of viral glycoproteins or intra-virion resident proteins. No crosslinks were observed linking extra-virion domains of viral glycoproteins to their intra-virion domains or to intra-virion resident proteins. **e**, Crosslink mapping onto structural models of UL55 in pre- and post-fusion conformations. See also Extended Data Fig. 2a–c. **f**, Network of PPIs inside HCMV particles. The line width (edges) scales with the number of identified crosslinks between interaction partners. Source data

**Extended Data Fig. 1:**
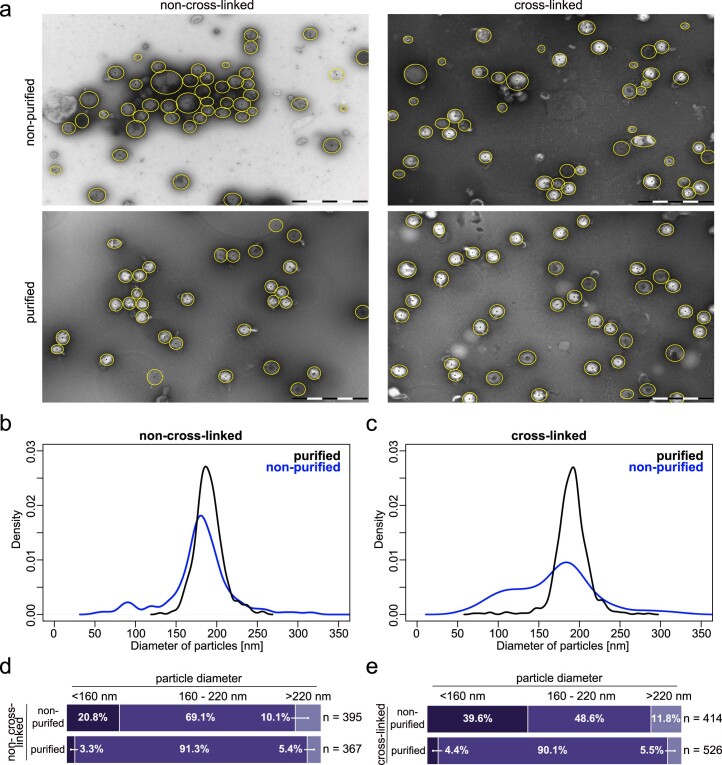
Estimation of sample purity from cross-linked and non-cross-linked HCMV particles. (**a**) Exemplary micrographs of negatively stained HCMV particles. Purification using glycerol tartrate gradients and cross-linking treatment as indicated. Scale bar represents 1000 nm. Manually selected particles are indicated by yellow circles. Representative micrographs of at least 10 per condition. (**b, c**) Distribution of diameters of manually picked particles of the non-cross-linked (b) or cross-linked (c) samples. Purification led to a more homogenous population of particle diameters. As previously noted^25^, we also find that the purification leads to membrane blebs, thus representing non-native particle morphologies. (**d, e**) Two cut-offs were defined to categorize unusually small (<160 nm) and large particles (>220 nm). These correspond to the median of diameters from the purified sample ± two times the standard deviation. The fraction of particles with diameters that fall below 160 nm, exceed 220 nm or fall in between are indicated for the non-cross-linked (d) or cross-linked (e) sample types. Purification results in more homogeneous particle diameters, enriching virions in the expected size range. Thus, our protocol captures the native configuration of the particles at relatively high sample purity. At least 367 particles were counted per condition. Source data

Therefore, we isolated extracellular particles from infectious cell culture supernatant and crosslinked them with disuccinimidyl sulfoxide (DSSO), which connects lysines from proteins in close spatial proximity. After purification of the crosslinked virions using tartrate-glycerol density gradient centrifugation, protein extraction and tryptic digestion, the crosslinked peptides were identified by liquid chromatography mass spectrometry (LC–MS) (Fig. 1a). We evaluated the quality of the preparations by performing negative-staining electron microscopy (EM) on crosslinked and/or gradient-purified particles (Extended Data Fig. 1).

We identified 9,643 unique lysine–lysine connections at a 1% false discovery rate (FDR) (Supplementary Table 1). First, we asked whether these data correctly captured the overall spatial organization of the virion (Fig. 1b) and counted the crosslinks between proteins having previously reported virion layer localization (Fig. 1c). We found most crosslinks within the tegument, followed by host proteins, the envelope and the nucleocapsid. Importantly, we did not observe any crosslinks connecting viral membrane proteins to viral nucleocapsid proteins, confirming that the crosslinks reflect the known layered virion architecture. Furthermore, we did not identify crosslinks connecting the interior of the virion to extra-virion domains of viral glycoproteins (Fig. 1d), providing confidence that the viral particles were not damaged before crosslinking. Host proteins contributed to crosslinks in all virion layers, confirming their association with herpesvirus particles^15–18^.

**Extended Data Fig. 2:**
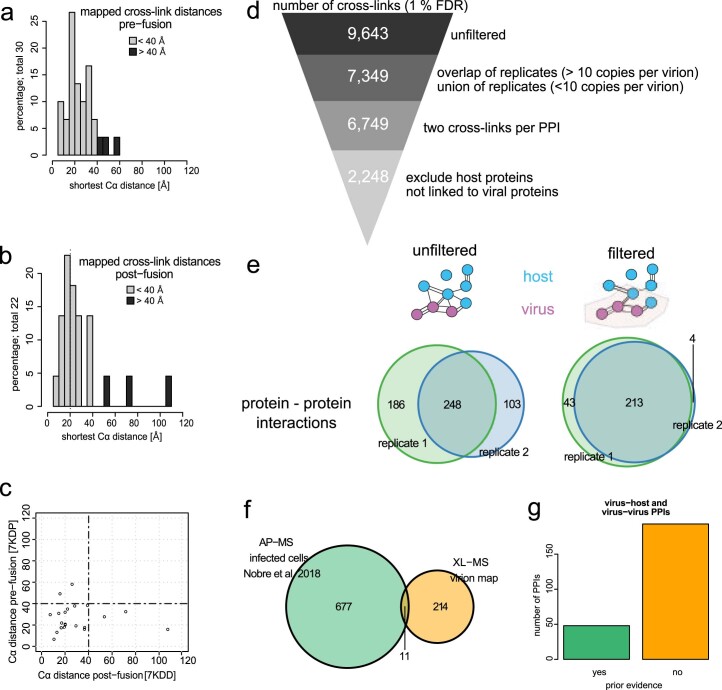
Data filtering and reproducibility. (**a, b**) Histogram of distances between Cα atoms obtained from mapping cross-links onto the cryoEM structural models of homotrimeric UL55 in pre- (a) or post-fusion (b) conformations (pdb: 7kdp, 7kdd). See Fig. 1e for visualization of the cross-links on the structural models. (**c**) Direct comparison of distances between pre- and post-fusion conformations. (**d**) The number of cross-links is shown that survive the indicated filtering steps. (**e**) Venn diagrams comparing the number of PPIs between both replicates in unfiltered (Supplementary Table 1) and filtered (Supplementary Table 2) dataset. Homomeric interactions (based on intra-links) were removed for this analysis. (**f**) Comparison to the AP-MS based interactome from infected cells^26^, based on viral baits included in the XL-MS PPI-map. (**g**) Number of PPIs with or without existing evidence in the literature. See also Supplementary Table 3.

Next, we investigated whether our XL–MS data are in agreement with published HCMV protein structures. Considering that two protein side chains can only be crosslinked if their mutual distance is small enough to be bridged by the crosslinker, the linear distance between the crosslinked lysines should be below 40 Å. To analyse whether this constraint is met, we mapped our crosslinks on structural models of the homotrimeric viral glycoprotein UL55 (also known as gB) in its pre- and post-fusion state^12^ (Fig. 1e and Extended Data Fig. 2a,b). We identified 16 crosslinks that agree with both the pre- and the post-fusion structures. In addition, 3 crosslinks could only be explained by the pre-fusion and 2 crosslinks could only be explained by the post-fusion structure (Extended Data Fig. 2c). These conformation-specific crosslinks confirm the previous finding that UL55 exists in different functional states^25^ and demonstrate that we capture biologically relevant structural states of viral proteins.

We then set out to build an XL-based map of intra-virion PPIs and applied further filtering criteria on our list of crosslinks to increase confidence (Extended Data Fig. 2d). This resulted in a reduced list of 2,248 crosslinks (Supplementary Table 2) supporting 260 PPIs (143 virus–host, 18 host–host, 99 virus–virus PPIs, Supplementary Table 3) with 82% overlap between two biological replicates (Extended Data Fig. 2e and Fig. 1f). Of the PPIs involving viral proteins, 5% were found in an affinity purification–MS (AP–MS) dataset from infected cells^26^ and an additional ~15% had previous evidence in the literature (Supplementary Table 3 and Extended Data Fig. 2f,g). The highly connected centre of the network contains tegument proteins that are frequently linked to each other and to neighbouring virion layers, in line with the characterization of the tegument as a highly interconnected module bridging nucleocapsid and viral envelope^27^. Viral membrane proteins as well as nucleocapsid proteins cluster independently and are in general much less connected.

## Allocation of viral proteins into distinct virion layers

While the localization and structure of the viral transmembrane and nucleocapsid proteins are well established, the spatial organization of the viral tegument is largely elusive. We hypothesized that the proximity-based nature of our data should enable the de novo allocation of viral tegument proteins to distinct layers within the particle.

**Fig. 2:**
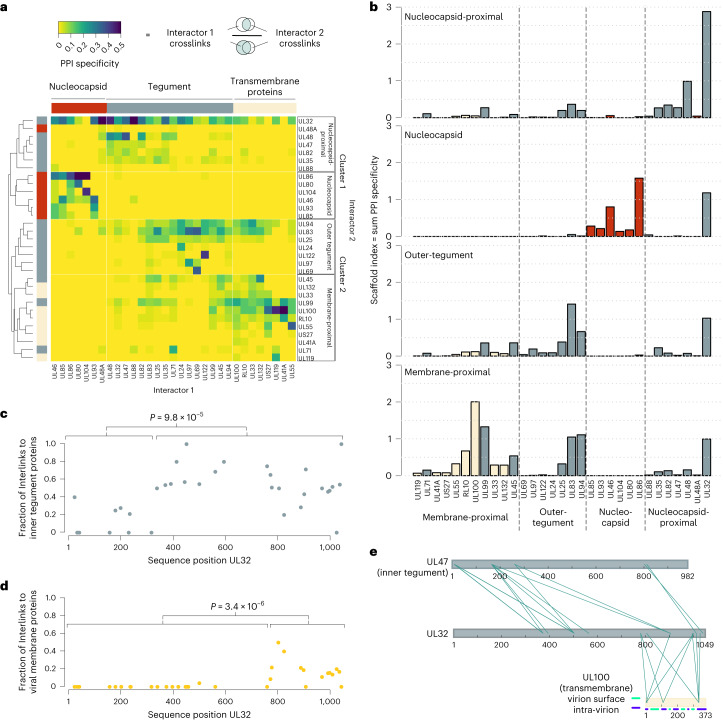
Spatial arrangement of viral proteins. **a**, Heat map of PPI specificity values of all viral proteins with at least 9 identified crosslinks. PPI specificity values were calculated for each PPI by dividing the number of crosslinks supporting a PPI by the total number of crosslinks of the interaction partner (‘interactor 1’). Interactor 2 proteins (rows) were clustered using 1-Pearson’s correlation distance and complete linkage. Interactor 1 proteins (columns) were manually annotated on the basis of previous knowledge. **b**, By summing up the PPI specificity values of interactor 2 proteins across all PPIs of the respective subcluster, a scaffold index was calculated and plotted using the same order of proteins as in the columns of **a**. Bar colours indicate the membership of proteins to nucleocapsid, tegument or viral envelope (transmembrane proteins), identical to **a**. **c**,**d**, For each individual lysine in UL32, the fraction of crosslinks in UL32 linking to other viral inner-tegument proteins (**c**) or viral membrane proteins (**d**) is plotted. *P* values are based on two-sided Wilcoxon rank-sum tests comparing the indicated lysines within the UL32 primary sequence. **e**, Crosslink network between UL32 and selected proteins of the inner tegument (UL47) and viral envelope (UL100). The membrane topology of UL100 is indicated by blue and green lines. Source data

To this end, we calculated ‘PPI specificity’ values for all viral PPIs as the ratio of crosslinks between two interaction partners (‘interactor 1’ and ‘interactor 2’) to the total number of crosslinks of the ‘interactor 1’ proteins (Fig. 2a). We treated each viral protein as interactor 1 (columns in Fig. 2a) and as interactor 2 (rows in Fig. 2a) and performed hierarchical clustering. This yielded two main clusters that can both be separated into two smaller clusters. As expected, nucleocapsid and viral transmembrane proteins separated into distinct main clusters (clusters 1 and 2 in Fig. 2a) and further contained two subclusters, supporting the view that the four subclusters represent the four architectural virion layers.

Focusing on the main cluster 1, we observed that nucleocapsid and tegument proteins form distinct subclusters. Only the smallest capsid protein UL48A, for which we detected relatively few crosslinks, co-clusters with the tegument group of cluster 1. Besides UL48A, this subcluster contained typical inner-tegument proteins such as UL32 and UL48, which are characterized by their tight association with the nucleocapsid^3^ as well as the UL48-associated protein UL47 (ref. ^28^), UL82 (also known as pp71), the UL82 interactors UL35 (ref. ^29^) and UL88, which are important for recruiting UL47, UL48 and UL82 into virions^30^. As UL82 and UL35 proteins traffic independently from the nucleocapsid upon infection^31,32^, their allocation to the nucleocapsid-proximal tegument layer indicates that proximity to the nucleocapsid does not necessarily reflect tight attachment upon cell entry.

The second main cluster (cluster 2) contained two subclusters, one with viral transmembrane proteins and tegument proteins, and the other with tegument proteins only. The first subcluster contained tegument proteins UL71, UL99 (also known as pp28) and UL45, indicating their membrane association, consistent with previous reports^33,34^. The second distinct subcluster represents the outer non-membrane-bound tegument. Its composition fits previous observations showing that the UL83 (also known as pp65) protein is important for incorporating UL69, UL97 and UL25 into mature particles^16,35^. Thus, on the basis of our cluster analysis, we define at protein-level resolution a distinct nucleocapsid layer and three tegument sublayers, including a nucleocapsid-proximal inner tegument, an outer tegument and a membrane-associated tegument. The identification of three separately organized tegument substructures points to a potential hierarchy in the herpesvirus tegumentation process.

After establishing the layer-specific organization of viral proteins, we asked which of these proteins are most important for the overall organization of PPIs. We reasoned that such proteins would act as scaffolds that specifically recruit other proteins. To find these, we summed up the ‘PPI specificity’ values of each viral protein in the respective subcluster (Fig. 2b), which revealed several main scaffolds within the individual layers: UL86 (also known as major capsid protein MCP) for the nucleocapsid, UL32 for the nucleocapsid-proximal tegument, UL83 for the outer tegument^16^ and UL100 (also known as gM) for the membrane-proximal proteins. Importantly, UL32 contributed to scaffolding in all layers and had the overall highest scaffold index, indicating that it serves as an organizational hub.

Despite its allocation to the nucleocapsid-proximal inner tegument, UL32 crosslinked with proteins from all layers of the virion (Fig. 2a, top row): nucleocapsid (for example, UL86), inner tegument (for example, UL48, UL47), outer tegument (for example, UL83) as well as viral glycoproteins (for example, UL55, UL100). Considering that UL32 is anchored with its N-terminal domain at the nucleocapsid^36^, we reasoned that the disordered C-terminal domain (amino acids 303–1,049) may engage in interactions with the other layers. Analysing the crosslinking partners of each UL32 lysine residue shows that residues 340–1,049 engage more frequently with other inner-tegument proteins (Fig. 2c). Likewise, a region in UL32 (residue 775-end) associates with viral membrane proteins (Fig. 2d). This indicates that a central region within UL32 locates to the tegument, whereas the more C-terminal distal region associates with both the tegument and the viral membrane. This domain-specific interaction pattern is further exemplified by its domain-selective crosslinking to an inner-tegument protein UL47 and a transmembrane protein UL100 (Fig. 2e). Thus, UL32 engages with all layers of the particle in an N-to-C-terminal radial orientation, bridging the nucleocapsid to the viral membrane.

## Layer-specific organization of host proteins

**Fig. 3:**
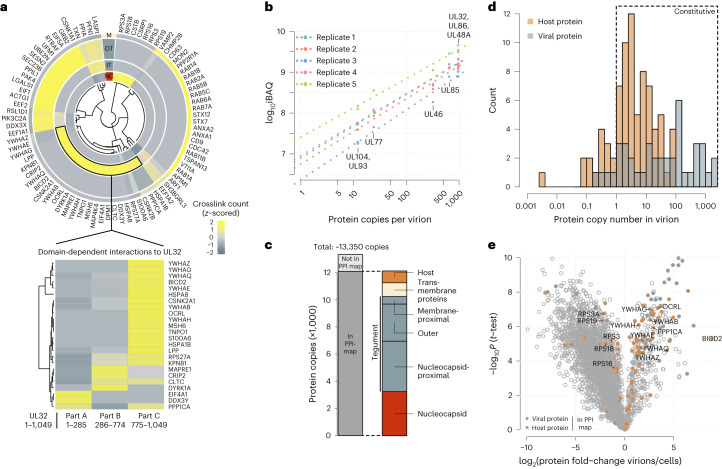
Abundant and layer-specific incorporation of host proteins. **a**, Top: heat map depiction of host protein localization across virion layers. Crosslink counts of host proteins to viral proteins of the respective layers (NC, nucleocapsid; IT, inner tegument (nucleocapsid-proximal tegument); OT, outer tegument; M, membrane, including viral transmembrane proteins and membrane-proximal tegument) were *z*-scored and hierarchically clustered using Euclidean distance. Bottom: *z*-scored crosslink counts of inner-tegument-localized host proteins to different parts of UL32. **b**, iBAQ of protein copy numbers using the copy numbers of nucleocapsid-associated proteins as standard (*n* = 2 biological replicates, with technical duplicates or triplicates). The offset between the replicates is caused by differences in the amount of input material, but the similar slopes demonstrate reproducibility of the quantitative data. **c**, Protein copies represented in the PPI map and their subvirion localization. **d**, Histogram of copy numbers from host and viral proteins. Proteins with more than one copy number on average are classified as likely constitutive. **e**, Relative quantification of proteins comparing virion lysates to cell lysates. Proteins included in the PPI map are highlighted. See Extended Data Fig. 4a for experimental design. Means of fold-change differences and *P* values of a two-sided *t*-test without multiple hypothesis correction are based on *n* = 4 biological replicates. Source data

**Extended Data Fig. 3:**
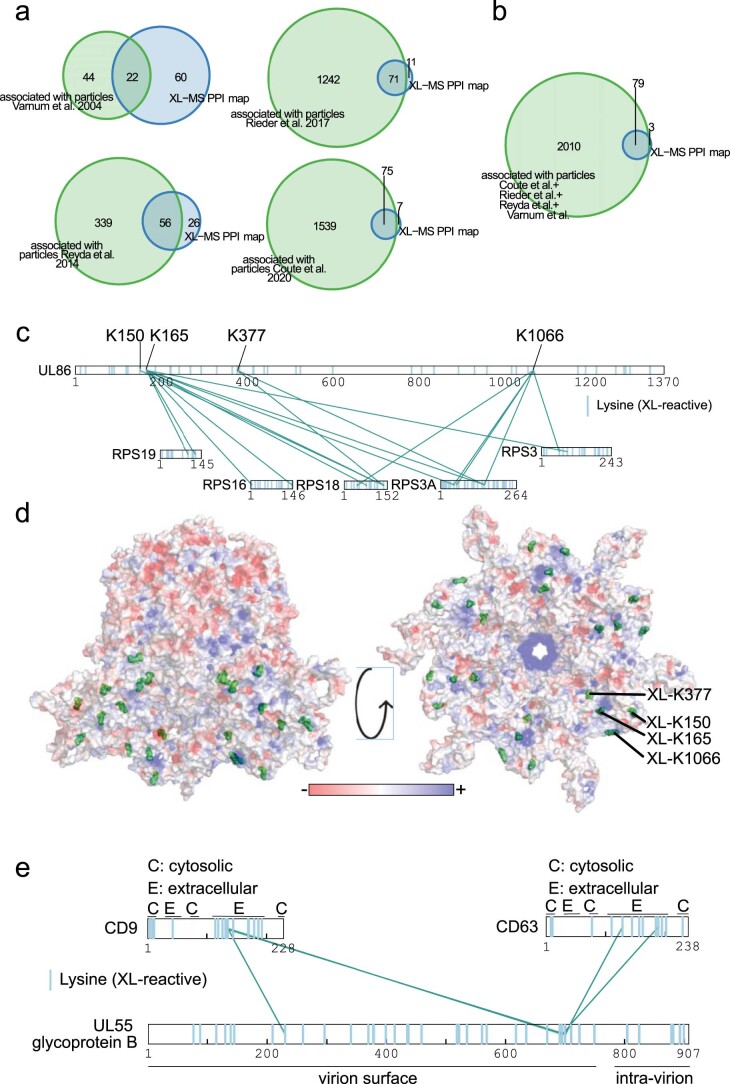
Host protein recruitment to HCMV particles. (**a, b**) Host proteins included in the PPI map (see Fig. 1f) were compared to host proteins from individual (a) or (b) combined published proteomics dataset^15–18^ of purified HCMV particles. (**c**) Cross-link network between ribosomal 40S proteins and UL86 protein. (**d**) Cross-linked lysines were mapped onto a nucleocapsid hexon (UL86 hexamer), depicted from top (left side) (that is inner tegument side) or bottom (right side) (that is DNA-accommodating side). Electrostatic surface rendering with charges depicted ranging from negatively charged (red) to positively charged (blue) (pdb 5vku). (**e**) Depiction of cross-links between glycoprotein UL55 and tetraspanins CD9 and CD63. Domain annotations were obtained from uniprot.org.

After establishing the organization of viral proteins within the particle, we turned to the 82 incorporated host proteins. Of these host proteins, 79 have been observed in previous proteomic studies as associated with purified virions using standard proteomics^15–18^ (Extended Data Fig. 3a,b). To determine their location, we first counted the crosslinks that individual host proteins had to viral proteins of the different layers, as established in Fig. 2a. To normalize for differences in the absolute number of crosslinks, the layer-specific link counts were then *z*-scored. Clustering these data resolved host protein localization to the individual layers (Fig. 3a). For example, ribosomal 40S proteins (RPS3A, RPS18, RPS16, RPS3, RPS19) specifically crosslinked with the viral nucleocapsid at four distinct lysine residues on the UL86 protein (Extended Data Fig. 3c). Mapping these crosslinks on a hexon structure revealed that ribosomal proteins associate with the nucleocapsid interior (Extended Data Fig. 3d), which is predominantly positively charged as it has to accommodate viral DNA. Thus, electrostatic interactions between negatively charged ribosomes and the positively charged nucleocapsid interior could explain this association.

The inner tegument incorporated a Lim-domain containing protein (LPP), heat-shock proteins (HSPA8, HSPA1), a cargo adaptor (BICD2) and clathrin (CLTC). In addition, we found various phospho-regulatory proteins in the inner tegument, such as 14-3-3 proteins (YWHAx, with x being either B, E, G, H, Q or Z), kinases (DYRK1A, casein kinases CSNK2B, CSNK2A1) and PP-1 (PPP1CA). Most of the inner-tegument-resident host proteins were crosslinked to UL32 predominantly to the region close to its C terminus (Fig. 3a, bottom).

The outer tegument contained proteins involved in innate immunity (DDX3X), translation factors (EEF2, EEF1A1, EIF5A, EIF1), chaperones (PPIA, PPIL1) and cytoskeletal proteins (ACTG1, PFN1, LASP1). At the viral envelope, we observed host proteins related to exo- and endocytosis and membrane trafficking, such as RAB-proteins (RAB1A/B, RAB2A, RAB5B/C, RAB6A, RAB11B, RAB14), adaptor proteins (AP1M1), snare proteins (VAMP2, VTI1A), annexins (ANXA1, ANXA2), syntaxins (STX7, STX12) and the tetraspanins TSPAN13, CD63 and CD9. Interestingly, the latter two tetraspanins were found to be crosslinked at the outside of the particle, associating with the UL55 virion surface domain (Extended Data Fig. 3e).

Collectively, this spatially resolved map of host proteins within virions indicates that host proteins are localized to different layers, reflecting the physical encounters of host proteins during different stages of virion assembly. Furthermore, UL32, in particular its C-terminal disordered region, engages in many interactions with host proteins, consistent with its scaffolding role for viral proteins.

## Quantitative assessment of host protein recruitment

After analysing the subvirion localizations of viral and host proteins, we aimed to characterize the quantitative dimension of their incorporation into the viral particle. To acquire an accurate protein inventory of the particle, we calculated copy numbers of host and viral proteins (Fig. 3b and Supplementary Table 4) and found that HCMV virions contain on average ~13,350 protein copies (Fig. 3c). Some 53 viral proteins are present with more than one copy per virion. Considering the spherical diameter of 200 nm, this results in a virion protein density of ~3.2 × 10^6^ proteins per cm^3^, similar to density estimates for mammalian and prokaryotic cells^37^. Of the copies, 91% belong to proteins that are contained in our virion network, indicating that we obtained a comprehensive spatial characterization of an average particle. Importantly, the vast majority of these proteins are incorporated with more than 1 copy per virion, indicating that these proteins are, on average, constitutive components (Fig. 3d).

**Extended Data Fig. 4:**
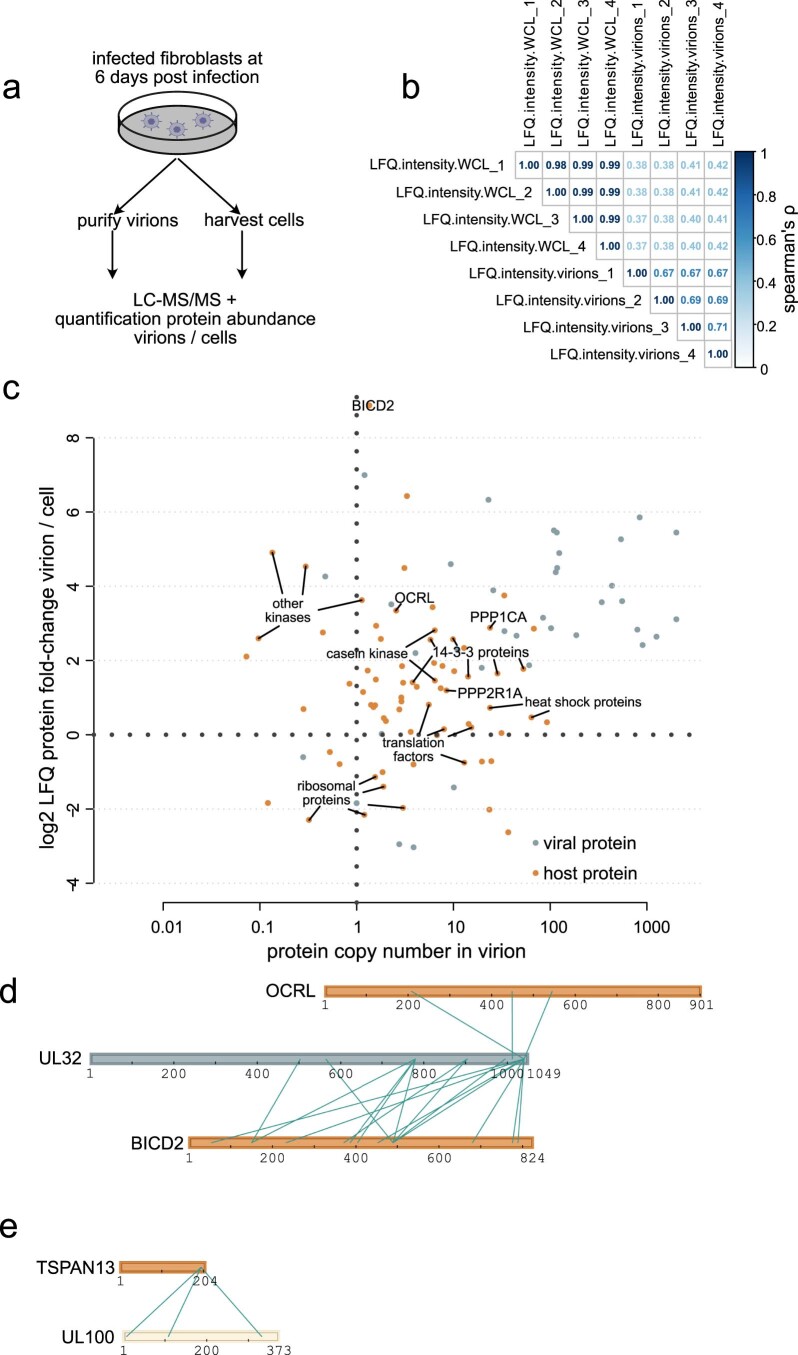
Quantitative proteomics identifies selective recruitment of host proteins. (**a**) Experimental setup to identify selectively recruited proteins (n=4 biological replicates). (**b**) Spearman’s correlation coefficients comparing log2 transformed LFQ-values of the experiment outlined in a. (**c**) Cross-comparison of protein copy numbers and enrichment levels of host and viral proteins. Selected proteins are depicted with their gene symbols. Enrichment levels are based on the mean of n=4 biological replicates and copy numbers based on the mean of n=2 biological replicates (with technical duplicates or triplicates). Enrichment level values below log2 -4 are not depicted. (**d, e**) Cross-link networks between selected host-virus PPIs.

We next asked whether host proteins are specifically targeted to virions, which may indicate functional relevance. We hypothesized that host proteins are incorporated either non-selectively, especially if they are highly abundant in the host cell, or on the basis of specific interactions with viral proteins promoting their recruitment. These scenarios can be distinguished by comparing the relative levels of host proteins in the virion and in cells, where increased protein levels in the virion relative to the cell are indicative of active recruitment. To address these options, we collected infectious cell culture supernatant for virion purification and the infected cells in parallel, and analysed both samples by quantitative proteomics (Extended Data Fig. 4a and Supplementary Table 5) with overall good reproducibility between replicates (Extended Data Fig. 4b). While most host and viral proteins contained in our spatial virion map were significantly enriched in virion lysates, several proteins were not enriched or were even depleted in virion preparations (Fig. 3e). Comparing enrichment levels to copy numbers (Extended Data Fig. 4c), we found that some proteins, such as heat-shock proteins, ribosomal proteins and translation factors, were constitutively incorporated but only to levels that reflect their cellular abundances, indicating that they are non-specifically packaged into virions.

In contrast, several low-abundant cellular proteins, such as the cargo transporter adaptor BICD2 and the phosphodiesterase OCRL, were strongly enriched in virions. While these proteins are not among the most abundant components of the virion, they exclusively crosslinked to one viral protein (Extended Data Fig. 4d,e), supporting the idea that they are actively recruited to the virion through specific PPIs. Overall, 39 of the 82 host proteins in the PPI map were more than 2-fold enriched in virions, indicating that they are selectively recruited.

## UL32 recruits 14-3-3 and PP-1 via specific binding motifs

**Fig. 4:**
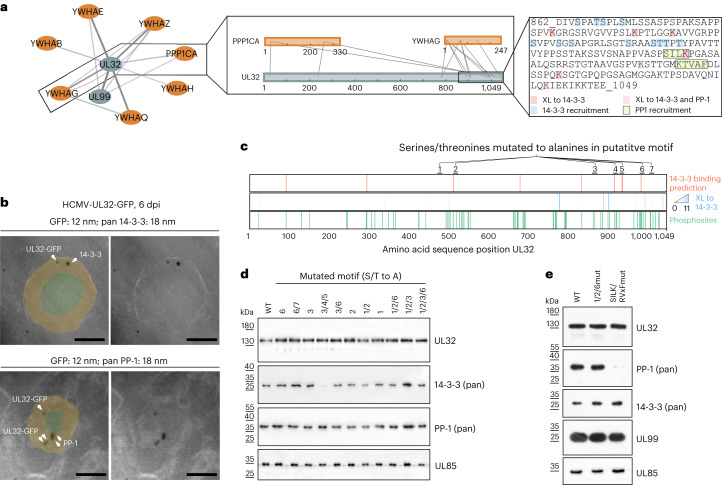
Nearby short linear motifs in UL32 recruit 14-3-3 and PP-1 proteins into virus particles. **a**, PPI network including interactors of PP-1 (PPP1CA) and 14-3-3 (YWHAx) proteins, with insets showing crosslinks involving UL32, 14-3-3 protein gamma (YWHAG), PP-1 and the UL32 sequence containing 14-3-3 and PP-1 recruitment sites. The line width scales with the number of identified crosslinks. Host and viral proteins are highlighted orange and grey, respectively. **b**, EM images of intracellular virions from HCMV-UL32-GFP-infected fibroblasts (MOI = 5). Ultrathin sections were stained with immunogold against GFP (12 nm gold) and either pan 14-3-3 or PP-1 (18 nm gold), as indicated. Approximate areas of tegument and nucleocapsid layers were manually highlighted in light orange and light green. Scale bars, 100 nm. See Extended Data Fig. 5a,b for uncropped micrographs. **c**, Mutational approach for identifying recruitment sites for 14-3-3 on UL32. Motif predictions above score 0.6 (top bar), crosslink positions to 14-3-3 (middle bar) and identified phosphorylation sites (localization probability >0.75 in *n* = 2 biological replicates) in virions (bottom bar). **d**,**e**, Purified virions from recombinant mutant viruses harbouring alanine substitutions in the designated motifs were assessed for protein levels by immunoblotting. Representative experiments of *n* = 2 biological replicates are shown. Source data

**Extended Data Fig. 5:**
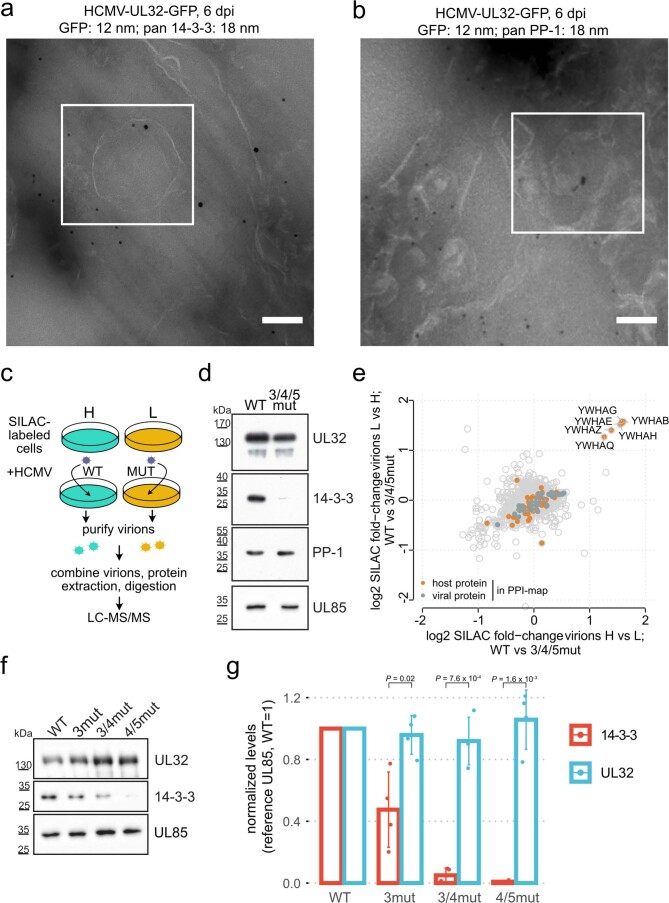
Characterization of the 14-3-3 binding-deficient viral mutants. (**a, b**) EM images of the intracellular environment from HCMV-UL32-GFP infected HELFs (MOI=5) prepared using Tokoyasu method. Ultrathin sections were stained with immunogold against GFP (12 nm gold) and pan 14-3-3 or PP-1 (18 nm gold), as indicated. Scale bars: 100 nM. Magnified views of the boxed regions in white are depicted in Fig. 4b. (**c**) Experimental workflow for SILAC-based comparison of virion protein content between mutant and WT viruses. The experiments were performed in label-swap duplicates. (**d**) Purified virions from WT or mutant viruses harbouring alanine substitutions in the ¾/5 region (see also Fig. 4c) were assessed for UL32, PP-1, 14-3-3 and UL85 levels by immunoblotting. Control experiment to panel (**e**). (**e**) SILAC-based proteomic comparison of log2 protein fold-changes comparing purified particles of WT and 14-3-3 binding mutants for both replicates, based on n=2 biological replicates. (**f, g**) Western blot analysis of abundance levels of selected proteins of purified virions for different 14-3-3 binding site mutant viruses. Exemplary blots in (**f**) and quantification based on n=2 biological replicates (with n=2 technical replicates) in (**g**). The height of the bars corresponds to the mean and the error bars to the standard deviation. P-values based on two-sided t-test without multiple hypothesis correction. Sites 4/5 are most important for 14-3-3 incorporation. Source data

We then selected PP-1 and 14-3-3 proteins for further investigation because they are among the most abundant host proteins that are selectively recruited into virions according to our quantitative data (Extended Data Fig. 4c). They crosslinked to the membrane-proximal tegument protein UL99 and, more frequently, to UL32 (Fig. 4a). We validated the incorporation of 14-3-3 and PP-1 into the tegument and their association with UL32 by double-immunogold labelling and electron microscopy analysis of intracellular HCMV particles (Fig. 4b and Extended Data Fig. 5a,b).

We hypothesized that PP-1 and 14-3-3 proteins are recruited via interaction motifs in UL32. Dimeric 14-3-3 proteins typically bind to phosphorylated serines or threonines within motifs that can be predicted by machine learning on the basis of the local sequence context^38^. Combining these predictions with information from our crosslink data and measured phosphorylation sites of UL32 in virions (Supplementary Table 6 and Fig. 4c), we hypothesized 7 potential motifs as 14-3-3 binding sites. We then mutated serines and threonines within these 7 potential motifs to alanines in different combinations in the viral genome. Combined mutation of three motifs abolished 14-3-3 incorporation into the particle (Fig. 4d). Comparison of the virion proteome of this mutant to wild-type (WT) viruses using quantitative proteomics based on stable isotope labelling by amino acids in cell culture (SILAC) further indicated that 14-3-3 proteins were selectively lost from mutant virions without strongly affecting other host or viral proteins (Extended Data Fig. 5c–g).

**Extended Data Fig. 6:**
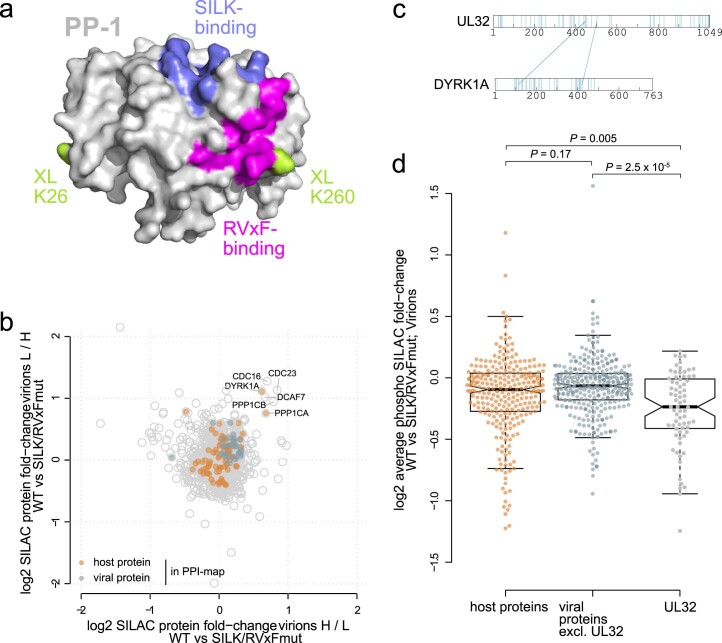
Characterization of the PP-1 binding-deficient viral mutant. (**a**) Crystal structure of PP-1 (pdb: 1fjm) with highlighted SILK and RVxF binding grooves as well as lysines cross-linked to UL32. (**b**) SILAC-based proteomic comparison of protein abundance comparing purified particles of wild-type and PP-1-binding mutant viruses. (**c**) Cross-links between DYRK1A kinase and UL32. Cross-link reactive lysines are highlighted blue in the sequence of both UL32 and DYRK1A. (**d**) Average log2 phosphosite fold-changes comparing wild-type and PP-1-binding mutant purified particles (centre line, median; box limits, upper and lower quartiles; whiskers, 1.5× interquartile range, overlaid with all data points). Phosphosites belonging to host proteins, viral proteins (excluding UL32) or to UL32 were compared using a two-sided Wilcoxon rank sum test. The average phosphosite ratio corrected by the protein ratio of n=2 biological replicates (including label-swap) are depicted. Experimental design as in Extended Data Fig. 5c with additional phosphopeptide enrichment.

Next, we investigated the recruitment of PP-1 into HCMV particles, a characteristic of HCMV^39^ with unknown mechanistic basis and biological significance. PP-1 has several surface grooves that bind to interactors containing for instance RVxF and SILK motifs^40^. In addition, UL32 harbours these motifs in its C-terminal 100 amino acids (Fig. 4a) and crosslinks in close spatial proximity to the RVxF binding groove (Extended Data Fig. 6a). Again, we mutated critical amino acid residues to alanine (SILK/RVxFmut) within these motifs, purified the virions and assessed the levels of PP-1. Incorporation of PP-1 was reduced in the mutant virus (Fig. 4e), which was confirmed by SILAC-based analysis of the whole proteome of mutant and WT virions (Extended Data Fig. 6b). The abundances of most other proteins were not altered in the mutant virions. However, PP-1 depletion correlated with depletion of DYRK1A, another specific crosslinking partner of UL32 (Extended Data Fig. 6c), and DYRK1A-interactors, such as the APC/C complex subunits CDC23 and CDC16 (ref. ^41^), and DCAF7 (ref. ^42^). A phosphopeptide analysis of these SILAC-labelled mutant and WT virions (Extended Data Fig. 6d) showed that PP-1 depletion correlated with significantly higher abundances of phosphopeptides derived from UL32 compared with other viral or host proteins, indicating that PP-1 preferentially dephosphorylates UL32. Thus, PP-1 is recruited to the particle via SILK/RVXF motifs that are in proximity to 14-3-3 recruitment motifs, confirming that host protein incorporation into viral particles is mediated by specific host–virus interactions.

## PP-1 recruitment regulates early and late events

**Fig. 5:**
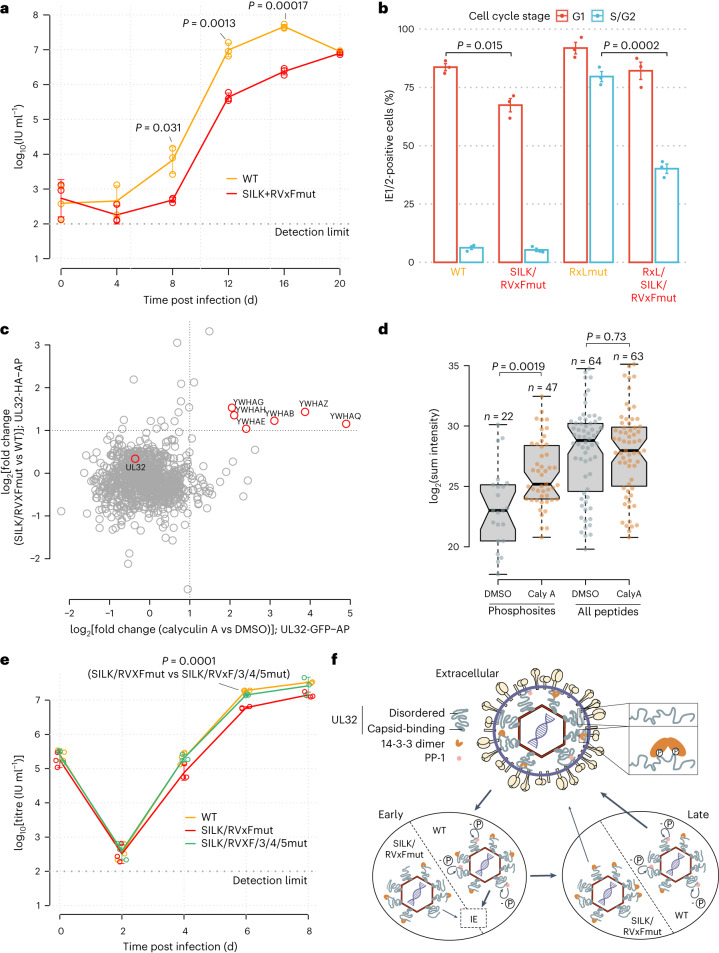
PP-1 recruitment controls early and late events during HCMV biogenesis. **a**, Growth curve of mutant and wild-type virus on HELFs (MOI = 0.05). Means ± s.d. of *n* = 3 biological replicates are depicted. Unpaired two-sided *t*-tests without multiple hypothesis correction were performed at the indicated timepoints. **b**, Flow cytometry analysis of IE protein levels as a function of the cell cycle stage. HELFs were infected with the indicated recombinant viruses (MOI = 5, 6 h post infection). The percentage of IE1/2-positive cells in G1 or S/G2 compartments is given, with mean ± s.e. of *n* = 3 biological replicates. Unpaired two-sided *t*-tests were performed comparing the fraction of IE1/2-positive cells between the indicated viruses and cell cycle compartments. Representative contour plots of one replicate are shown in Extended Data Fig. 7c. **c**, Comparison of differential interaction partners to UL32 upon phosphatase inhibition (*x* axis) or genetic disruption of the SILK/RVxF motifs (*y* axis). See Extended Data Fig. 9 for volcano plots, experimental design and controls. **d**, Abundance levels of quantified phosphosites or quantified peptides of pp150/UL32 are depicted with their sum intensity across *n* = 3 biological replicates in UL32-GFP precipitates (centre line, median; box limits, upper and lower quartiles; whiskers, 1.5× interquartile range). *P* values from two-sided Wilcoxon rank-sum test as indicated. **e**, Same as **a** but with MOI = 5. **f**, Location of 14-3-3 and PP-1 inside virions and their role during early and late stages of HCMV infection. WT viruses, able to recruit PP-1, dephosphorylate (-P) UL32 efficiently (bold arrows), start viral gene expression (IE) and produce infectious progeny. PP-1-binding-deficient RVxF/SILKmut viruses are impaired (light arrows) at the start of IE gene expression and production of novel progeny. No adjustments for multiple comparisons were performed for **a**, **b**, **d** and **e**. Source data

**Extended Data Fig. 7:**
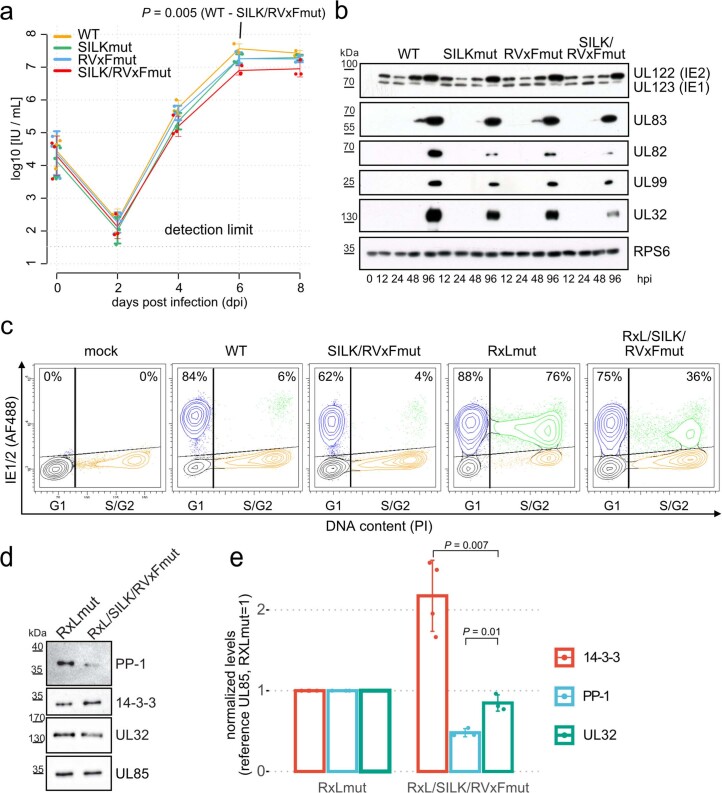
PP-1-UL32 binding promotes viral growth and the onset of viral gene expression. (**a**) Growth curve of mutant and wildtype viruses on HELFs (MOI=5). Means (centre of the error bars) and standard deviations of the mean of n = 3 biological replicates are depicted. Unpaired two-sided t-tests without multiple hypothesis correction were performed comparing the indicated viruses and time points. (**b**) Kinetics of viral protein levels of wildtype and mutant viruses across the replication cycle (MOI = 5). The experiment was replicated at least three times with similar results. (**c**) Flow cytometry analysis of Immediate Early (IE) protein levels as a function of the cell cycle stage. HELFs were infected with the indicated recombinant viruses (MOI = 5, 6 h post infection). The percentage of IE1/2-positive cells in G1 or S/G2 compartments is given. (**d, e**) Western blot analysis of abundance levels of selected proteins of purified virions for RxLmut and RxL/SILK/RVxFmut viruses. Exemplary blots in (d) and quantification based on n=4 (14-3-3) or n=3 (PP-1, UL32) technical replicates in (e). The height of the bars corresponds to the mean and the error bars to the standard deviation. P-values based on two-sided t-tests without multiple hypothesis correction. Control experiments to Fig. 5b. Source data

To test the functional relevance of the UL32-controlled recruitment of PP-1 to viral particles, we first investigated the ability of the UL32-PP-1-binding-deficient mutant virus (SILK/RVXFmut) to replicate in cell culture. Compared with the parental WT virus, the mutant virus produced up to 20-fold fewer viral progeny at 12 d post infection and low multiplicity of infection (MOI) (Fig. 5a and Extended Data Fig. 7a). Consistently, the kinetics of viral gene expression was delayed in the mutants (Extended Data Fig. 7b). Together this supports that recruitment of PP-1 to UL32 is functionally important for efficient production of viral progeny.

While virion-delivered PP-1 and UL32 enter the cell as part of the tegument, new copies of UL32 are produced in the infected cell only during later infection cycles. PP-1-UL32 binding might thus be important during (1) events directly after entry before viral gene expression has started, (2) during late stages when new copies of UL32 are produced or (3) both.

**Extended Data Fig. 8:**
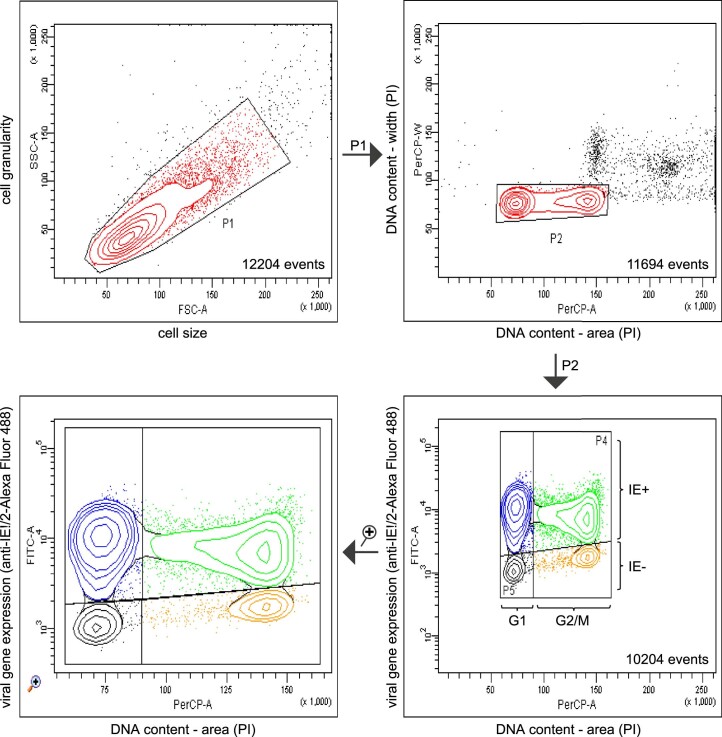
Hierarchical gating strategy for analysis of cell cycle-dependent viral gene expression by flow cytometry. First, a contour plot was created displaying on a linear scale the forward light scatter (FSC) and sideward light scatter (SSC) of measured particles. A population P1 was gated that excludes damaged cells and cell aggregates from further analysis. Based on the area (A) and width (W) of the propidium iodide (PI) fluorescence signal (PerCP channel), a cell population P2 was defined that excludes cell doublets and cells with a greater than 2n DNA content from further analysis (upper right contour plot). Based on cellular DNA content and on the Alexa Fluor 488 fluorescence (FITC channel) from IE1/IE2 immunostaining, four subpopulations of P2 were defined (lower right contour plot): P3 consists of IE-positive G1 cells; P4 of IE-positive G2/M cells, P5 of IE-negative G1 cells, P6 of IE-negative G2/M cells. For better visibility, these four cell populations were depicted in a ‘zoom-in’ representation mode (lower left contour plot). The described gating strategy was used for all flow cytometry data presented in Fig. 5b and Extended Data Fig. 7c. Contour levels were set to 15% probability. Outliers falling outside the lowest contour level are displayed as dots.

We first focused on early events and assessed the production of the very first viral antigens (IE1/2 proteins). As the onset of HCMV gene expression is blocked during S/G2 phases of the cell cycle^43^, we analysed IE1/2 levels throughout the cell cycle by flow cytometry analysis (Fig. 5b, and Extended Data Figs. 7c–e and 8). During susceptible G1, the WT virus accumulated immediate early (IE) proteins in 84% of cells, which dropped to 62% in PP-1-binding-deficient SILK/RVxFmut virus. To assess the contribution of PP-1 during the non-susceptible stage S/G2, we analysed the PP-1-binding-site mutations in the genetic background of a virus able to start IE1/2 gene expression in S/G2 (ref. ^44^). Integrating the PP-1-binding-site mutations into this backbone (RxLmut) led to a decrease in the fraction of cells expressing IE1/2 proteins from 76% to 36% during S/G2. Together, this implicates UL32-recruited PP-1 phosphatase as crucial for the onset of viral gene expression.

**Extended Data Fig. 9:**
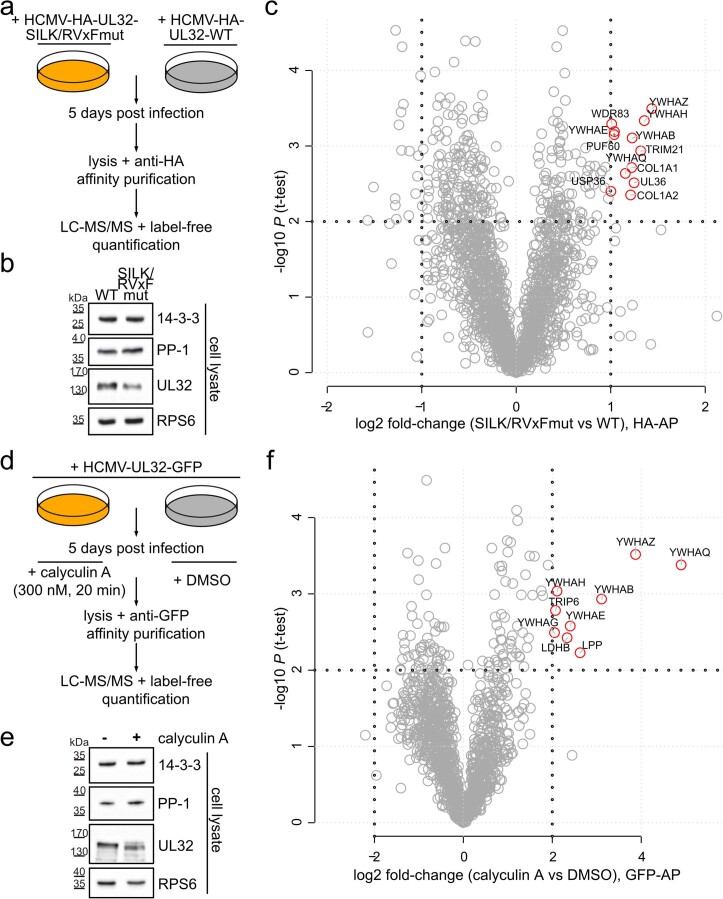
Recruitment of PP-1 functionally antagonizes 14-3-3 binding to UL32. (**a, d**) Experimental strategy to quantify changes in the UL32 interactome that were induced by loss of PP-1 binding sites (**a**) or pharmacologic inhibition of PP-1 phosphatase activity (**d**). Both experiments were performed in n=3 biological replicates. See also Fig. 5c,d. (**b, e**) HCMV infected cells were lysed at 5 days post infection. Before the cell lysates were used as input for HA or GFP affinity purification, they were controlled for UL32, PP-1 and 14-3-3 expression by immunoblot analysis. The ribosomal protein RPS6 indicates equal loading. Experiments were replicated with similar results at least three times. (**c, f**) Volcano plot analysis of LC-MS/MS data showing the average log2 fold-change of identified proteins and the corresponding p-values of a two-sided t-test without multiple hypothesis correction. Cut-offs were set manually at P = 0.01 and log2 fold-change = 1 (c) or log2 fold-change = 2 (f). Proteins passing these cut-offs were labelled with gene symbols. Source data

We then explored the role of the PP-1–UL32 association during later stages of the infection cycle and asked whether PP-1 modulates the interactions of UL32 with other proteins. To this end, we performed two interaction proteomics experiments from virus-infected cells: first, comparing the interactors of WT-UL32 to the SILK/RVxFmut-UL32 (Extended Data Fig. 9a–c) and second, comparing the interactors of WT-UL32 under treatment with phosphatase inhibitor calyculin A to a control (Extended Data Fig. 9d–f). We observed that both the genetic disruption of PP-1-UL32 binding and chemical inhibition of phosphatase activity led to increased presence of 14-3-3 on UL32 (Fig. 5c), indicating that the presence of active PP-1 phosphatase limits the association of UL32 with 14-3-3 proteins. Consistently, phosphatase inhibition led to more and higher-abundant phosphopeptides of UL32 (Fig. 5d). To assess the functional relevance of this, we created a virus in which both the PP-1 and the 14-3-3 binding sites were mutated (SILK/RVxF/3/4/5mut). Comparing this mutant to the PP-1-binding-deficient but 14-3-3-binding-competent SILK/RVxFmut virus showed that abolishing 14-3-3 recruitment rescued viral multiplication to almost WT levels (Fig. 5e). Thus, the phosphatase protects HCMV from overloading 14-3-3 proteins onto the inner tegument (Fig. 5f), implying a perturbing or antiviral role for 14-3-3 proteins, antagonizing efficient production of viral progeny.

## A coarse-grained model of the virion

**Fig. 6:**
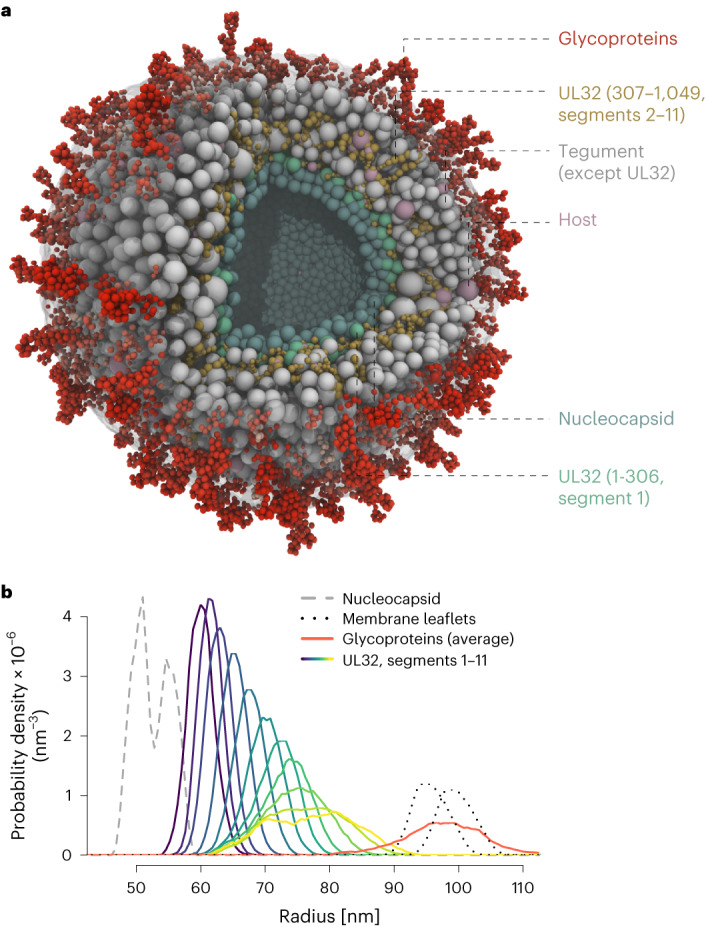
A coarse-grained model of the HCMV virion. **a**, Cross-sectional view through the equilibrium state with transparent membrane. Bead species identity as indicated. UL32 C-terminal segments make occasional contacts with intra-virion domains of viral glycoproteins. **b**, Probability distributions of UL32 segments (see Extended Data Fig. 10) in the tegument. Nucleocapsid and membrane layers are given as reference. Source data

**Extended Data Fig. 10:**
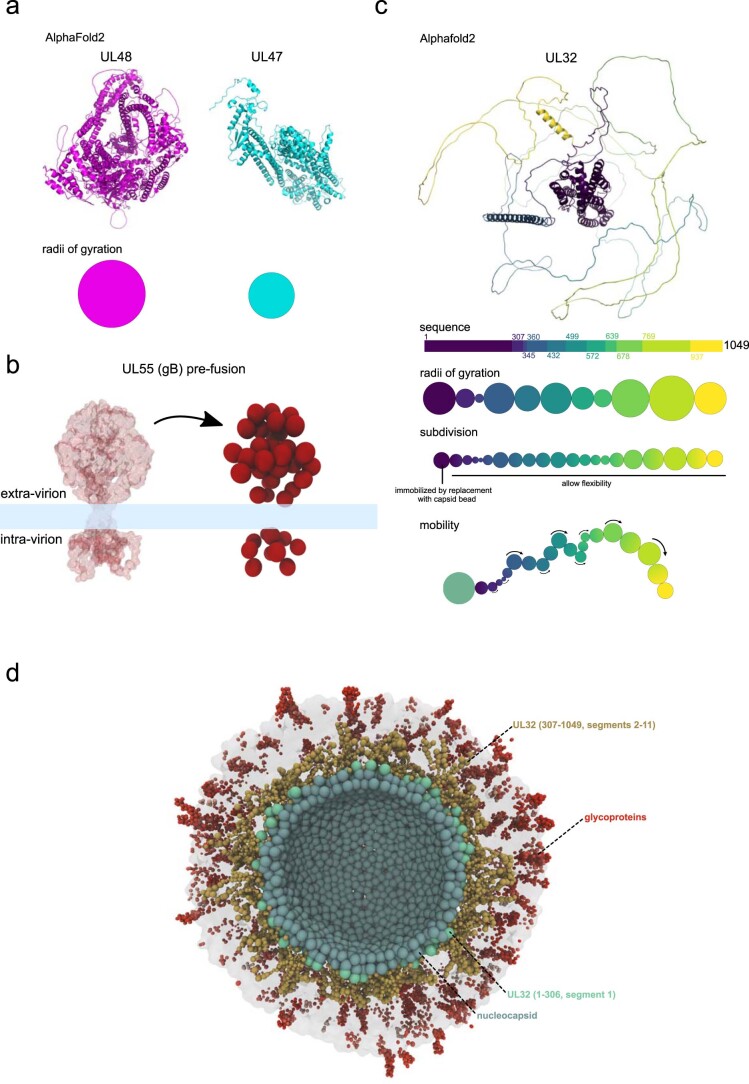
Parametrization of proteins for coarse-grained modelling. (**a**) Bead sizes for tegument and host proteins other than UL32 were approximated by the gyroscopic radii from their corresponding AF2 predictions, which is exemplarily shown for UL47 and UL48 proteins. (**b**) Representation of glycoproteins in the coarse-grained model via clustering of ɑ-carbon from cryoEM or AF2 models into beads, exemplarily shown for gB pre-fusion structure. The transmembrane domains are substituted by beads from the membrane model (light blue). (**c**) The UL32 AF2 prediction was segmented into 11 parts based on a molecular dynamics simulation. Segment dimensions were calculated from the segment-specific radii of gyration. As the radius of gyration overestimates the volume for a disordered protein, the chain of beads was further subdivided to yield a chain of more but smaller beads. The most n-terminal bead was immobilized by replacing it with a bead from the nucleocapsid shell. The chain of beads was allowed to have segment-specific flexibility at hinges (indicated arrows). (**d**) Cross-sectional view of the HCMV virion model with tegument proteins other than UL32 removed.

We then aimed to integrate our datasets into a unified picture of the particle. Therefore, we performed coarse-grained modelling of protein components within a 190 nm sphere enclosed by a lipid bilayer^45–47^, guided by the proteomics-based copy number estimates as well as pairwise bead interaction constraints from our crosslinking data (see Methods) (Fig. 6a). Tegument and host proteins are presented as individual beads whose size corresponds to the proteins’ radii of gyration derived from Alphafold2 (AF2) (ref. ^48^) models (Extended Data Fig. 10a). Sizes and shapes of glycoproteins and the nucleocapsid were approximated by multiple beads on the basis of AF2 or cryoEM models^8,13,49^ (Extended Data Fig. 10b). To account for the long and disordered C-terminal domain of UL32, we generated a flexible model of UL32 allowing for domain-specific interactions to other proteins in the virion (Extended Data Fig. 10c).

After placing the beads with a self-avoiding random distribution inside the particle, we simulated Brownian dynamics of diffusing and interacting particles, allowing for the system to relax to its free energy minimum. Sampling the system configurations afterwards (Supplementary Video 1) provides likely localizations for individual domains of UL32. They have a wide distribution across the tegument, with the C-terminal parts contacting the nucleocapsid or the intra-virion domain of viral glycoproteins (Fig. 6b, Extended Data Fig. 10d and Supplementary Video 2). Thus, the integration of copy numbers, XL–MS-based PPI information, structural predictions and biophysical constraints into a unified model supports the role of UL32 in tegument organization.

We integrated XL–MS and quantitative proteomics with molecular virology to provide detailed insights into the stoichiometry, architecture and domain-level PPIs of an infectious HCMV particle. Our study addresses long-standing questions regarding the organization of herpesvirions, as it (1) enabled de novo reconstruction of the layered virion architecture including the spatial organization of the structurally complex viral tegument, (2) allowed comprehensive mapping of host–virus PPIs and their interaction contacts in the native configuration of an intact virion and (3) demonstrated the biological significance of recruited host proteins.

On the basis of our unbiased clustering of the viral tegument, we catalogued tegument proteins into three sublayers: a nucleocapsid-proximal inner, an outer and a membrane-proximal tegument. While many of the allocations are in congruence with previous observations^16,30,33–35^, some were unexpected. For example, pp71/UL82 and UL35 clustered to the nucleocapsid-proximal tegument but have previously been associated with typical outer-tegument properties, such as detergent solubility and nuclear trafficking^3,31,32^. In this context, it is interesting that the release of pp71/UL82 from the nucleocapsid-proximal tegument may have regulatory potential^50^. Remarkably, we observed that the different tegument sublayers are bridged by UL32. While its N-terminal domain is tightly capsid-bound^36^, its C-terminal 250–300 amino acids are associated with viral envelope proteins. The capsid-to-membrane tethering architecture of UL32 may be critical for the structural integrity of the virion and may also explain why UL32 is important during cytosolic maturation events^51,52^. The UL32 C-terminal region was found crosslinked not only to envelope but also to tegument proteins, suggesting that XL–MS captured different co-existing functional UL32 states as also observed for UL55^25^. In agreement with its disordered state^44^, our model proposes that the UL32 C terminus is thus likely to adopt multiple structural arrangements, of which a fraction makes membrane contact.

This structural flexibility of UL32, paired with its high abundance and considerable sequence length, allows it to act as the dominant scaffold in the virion, organizing many interactions with other viral and cellular proteins. Importantly, UL32 associated with a variety of phospho-regulatory proteins and recruited 14-3-3 and PP-1 via nearby binding sites in its C terminus. PP-1 is a target that is exploited by various viral proteins, such as HSV-1 ICP34.5 (ref. ^53^), HSV-1 pUL21 (ref. ^54^), measles/Nipah virus V protein^55^ and HIV Tat^56^. This regulates phospho-sensitive processes such as translation^53^, transcription^56^ and RNA sensing^55^. Recruitment of PP-1 to UL32 is important for high-titre replication of HCMV (Fig. 5a), consistent with previous inhibitor experiments targeting the substrate recruitment site of PP-1 (ref. ^57^). We show that this interaction modulates the phosphorylation status of UL32 and positively regulates the onset of viral gene expression (Fig. 5b,d and Extended Data Fig. 6d).

At the late stage of infection, PP-1 functionally and biochemically antagonizes the binding of 14-3-3 to UL32, indicating that HCMV recruits PP-1 to limit the antiviral or perturbing effects of 14-3-3. It is conceivable that overloading of the rigid 14-3-3 proteins might structurally restrain the flexible UL32 C terminus and impair efficient virion assembly. These findings illustrate how host–virus interactions found within viral particles are relevant for early and late events of the replicative cycle.

Besides 14-3-3 and PP-1, we found many other host proteins associated with the virion, ranging from the nucleocapsid interior to the virion surface. Such host proteins are constitutively incorporated on the basis of two main routes. First, specific host–virus interactions lead to the selective enrichment of host proteins irrespective of their cellular abundance. One example is the cargo-adapter-protein BICD2, which is known to facilitate trafficking and nuclear import of HIV-1 genomes^58^. We found that it specifically associated with UL32, suggesting that it may allow movement of the incoming HCMV nucleocapsid along microtubules, in analogy to cellular kinesin in HSV-1 virions^6^. Second, non- or low-specific host–virus interactions lead to incorporation of highly abundant cellular proteins that are not or are only modestly enriched. Among these are ribosomal proteins that we found associated with the DNA-accommodating side of the capsid. This association is substoichiometric and consistently, ribosomal proteins are not overrepresented in virions compared with cells. In addition, HCMV virions contain RNA species^59,60^, an aspect that our study did not cover.

Comparing our XL–MS dataset from intact virions to a previously published large-scale AP–MS dataset from infected cells^26^ reveals a modest overlap in PPIs of ~5%. The complementarity of the XL–MS and AP–MS data is unsurprising for several reasons. First, they comprise PPIs from lysed infected cells (AP–MS) and intact virions (XL–MS)—different systems with divergent relative abundances of host and viral proteins. Second, AP–MS requires cell lysis, washing and affinity purifications, possibly causing loss of low-affinity interactions and formation of PPIs that may not exist in the intact cell. In contrast, XL–MS identifies contact sites of proteins in their intact environment, usually retaining low-affinity interactions. Third, the complexity of crosslinked peptides makes it challenging to identify crosslinks from low-abundant proteins, which might still be captured by AP–MS. In our situation, this appears not to be particularly problematic, as our map included >91% of the protein copies inside a virus particle.

The understanding of the HCMV virion architecture has substantially improved through recently reported atomic structures of the nucleocapsid^8^ and viral glycoproteins^12,13,61^. Expanding on this knowledge, our study provides global insights into the organization of proteome, interactome and host protein recruitment. By integrating these data, we developed a coarse-grained model that accounts for biophysical constraints and recapitulates tegument organization by UL32. This model serves as a blueprint that can incorporate further structural and molecular details as they emerge. We anticipate that XL–MS will be a valuable tool for studying other viral particles, in particular, complex, large and enveloped viruses.

## Methods

### Cells and viruses

Human embryonic lung fibroblasts (HELFs) were maintained as previously described^62^. In preparation for SILAC proteomic analysis, cells were SILAC-labelled for at least five passages using lysine and arginine-deprived DMEM medium, supplemented with 10% dialysed serum (cut-off: 10 kDa, PAN-Biotech), heavy (l-[^13^C_6_,^15^N_2_]-lysine (Lys8), l-[^13^C_6_,^15^N_4_]-arginine (Arg10)) or light (natural lysine (Lys0) and arginine (Arg0)) amino acids. For phosphoproteome analysis, we supplemented DMEM medium with 200 mg l^−1^ l-proline. Labelling efficiency was checked using LC–MS/MS. HELF cells were also used for preparation of viral stocks. The HCMV strain TB40-BAC4 (ref. ^63^) was used for all experiments. Infectious virus titres were determined from extracellular virus by immunotitration and indicated as IE-forming units (IU) per ml, as previously described^64^. Infection experiments were carried out using either a high (5 IU per cell) or low (0.05 IU per cell) MOI. Bacterial artificial chromosome mutations were created by traceless mutagenesis according to established protocols^65^. Mutations were verified by Sanger sequencing and PCR. See Supplementary Table 7 for a list of mutagenesis primers and an overview of viral mutants in this study.

### Immunoblotting

Gradient-purified virions were resuspended in PBS and adjusted to equal concentrations on the basis of their optical density at 600 nm wavelength. Then, equal virion amounts were centrifuged for 60 min at 35,000 r.p.m. (~105,000 × *g*) using a TLS-55 rotor (Beckman). The virions were lysed by sonication in 50 mM Tris-Cl (pH 6.8), 2% sodium dodecyl sulfate (SDS), 10% glycerol, 100 mM dithiothreitol, 2 µg ml^−1^ aprotinin, 10 µg ml^−1^ leupeptin, 1 µM pepstatin, 0.1 mM Pefabloc and bromophenol blue, and boiled at 95 °C for 3 min. The samples were resolved by SDS–PAGE and blotted to polyvinylidene fluoride membranes. To prevent non-specific binding, blots were incubated in Tris-buffered saline, 0.1% Tween-20 and 5% skim milk. The following primary antibodies were used: anti-UL32 (clone XP1, 1/3,000), anti-UL85 (polyclonal, 1/2,000), anti-PP-1 (clone E9, sc-7482, Santa Cruz, 1/400), anti-14-3-3 (clone H8, sc-1657, Santa Cruz, 1/400), anti-RPS6 (clone 5G10, Cell Signaling, 1/1,000), anti-UL82 (clone 2H10-9, 1/100), UL83 (clone CH12, sc-56976, Santa Cruz, 1/1,000) and UL122/123 (clone E13, Biomerieux, 11-003, 1/4000). Blots were developed using horseradish peroxidase-conjugated secondary antibodies in conjunction with suitable enhanced chemiluminescence detection systems.

### Flow cytometry

Flow cytometric analysis of DNA content and IE1/IE2 expression was carried out as described previously^62^. In brief, cells were collected by trypsinization, fixed and permeabilized by incubation in 75% ethanol for at least 12 h at 4 °C and stained with specific antibodies and propidium iodide. Alexa Fluor 488-conjugated anti-IE1/IE2 (clone 8B1.2, MAB810X, Merck, 1/1,000) was used. Flow cytometry was performed using a FACSCanto II instrument and FACSDiva software (both from BD Biosciences). Cellular debris, cell doublets and aggregates were gated out of analysis (see Extended Data Fig. 8 for gating strategy).

### Virion purification and crosslinking

To prepare HCMV particles for in situ crosslinking, we first collected infectious cell culture supernatants and clarified them of cellular debris by centrifugation at 1,500 r.c.f. for 10 min. The remaining viral supernatant was centrifuged for 1 h at 25,000 r.p.m. (~112,500 × *g*) in an SW-28 rotor (Beckman). The resulting virus pellets were resuspended in 1–2 pellet volumes of PBS and supplemented with 2.5 mM DSSO (100 mM stock solution in dimethyl sulfoxide). The crosslinking reaction was incubated for 30 min at 25 °C under shaking conditions (1,000 r.p.m.). The crosslinking step was repeated once with additional 2.5 mM DSSO before the reaction was quenched with 50 mM Tris-HCl (pH 8.0) for 20 min at 25 °C under constant agitation (1,000 r.p.m.). Subsequently, the crosslinked material was loaded onto glycerol-tartrate gradients as described elsewhere^66^ and centrifuged for 1 h at 25,000 r.p.m. (~111,000 × *g*) in an SW-40 rotor (Beckman), with brakes set at the slowest possible deceleration. The virion band was aspirated through the wall of the ultracentrifuge tube using a 1 ml insulin syringe equipped with a 1.2 ×40 mm needle. The virion fraction was washed twice in PBS. The first washing step included virion sedimentation at 30,000 r.p.m. (~121,000 × *g*) in an SW-60 rotor (Beckman) and the second step, sedimentation at 35,000 r.p.m. in a TLS-55 rotor (77,000 × *g*). The purified virions were stored at −80 °C for XL–MS sample preparation (see below). A detailed step-by-step protocol is provided in Supplementary Information.

### Virion sample preparation for XL–MS and bottom-up proteomics

First, to increase proteomic coverage of glycoproteins, crosslinked virion samples were deglycosylated using Protein Deglycosylation Mix II (P6044, NEB) under denaturing conditions according to manufacturer instructions. Lysis of virion samples was performed by adding 3 volumes of lysis buffer containing 8 M Urea, 1% Triton X-100, 30 mM chloroacetamide (CAA), 5 mM tris(2-carboxyethyl)phosphine hydrochloride (TCEP) and 700 units Benzonase (70746, Merck), and incubating on ice for 30 min followed by sonication for 45 min (30 s on, 30 s off) in a Bioruptor Pico (Diagenode) at 4 °C. Proteins were extracted using methanol–chloroform precipitation according to standard protocols^67^, dried and resuspended in digestion buffer (50 mM triethylammonium bicarbonate, pH 8.0, 1% sodium deoxycholate, 5 mM TCEP and 30 mM CAA). Proteins were digested by adding trypsin at an enzyme-to-protein ratio of 1:25 (w/w) and LysC at a 1:100 ratio (w/w) at 37 °C overnight in the dark. Peptides from crosslinked samples were desalted using Sep-Pak C8 cartridges (Waters). Peptides from non-crosslinked samples were desalted using C18 stage-tip purification followed by LC–MS.

Peptides destined for crosslink analysis were further fractionated by strong cation exchange using a PolySULFOETHYL A column (PolyLC) on an Agilent 1260 Infinity II system. A 90 min gradient was applied and 33–35 fractions were collected, desalted by C8 stage tips, dried under speed vacuum and subjected to LC–MS analysis.

### Cell sample preparation for LC–MS

Cells were collected at 6 d post infection by scraping in PBS. Cell lysis, protein extraction and digestion were performed exactly as described above for virions.

### AP–MS

HELFs were infected with HCMV-UL32-GFP (phosphatase-activity-dependent interactome, GFP-AP), HCMV-UL32-HA or HCMV-UL32-SILK/RVxFmut-HA (PP-1-binding-dependent interactome, HA-AP) at an MOI of 5 IU per cell. Experiments were performed in *n* = 3 replicates, with one confluent 15 cm dish as starting material per replicate and experiment. At 5 d post infection, cells were treated with 300 nM calyculin A (BML-EI192-0100, Enzo Life Sciences) for 20 min or left untreated. Directly after, cells were collected by scraping in PBS and processed as previously described^68^. In brief, cells were washed in PBS and lysed for 20 min in lysis buffer (25 mM Tris-HCl (pH 7.4), 125 mM NaCl, 1 mM MgCl_2_, 1% Nonidet P-40, 0.1% SDS, 5% glycerol, 1 mM dithiothreitol, 2 µg ml^−1^ aprotinin, 10 µg ml^−1^ leupeptin, 1 µM pepstatin, 0.1 mM Pefabloc, 0.5 mM Na_3_VO_4_, 10 mM β-glycerophosphate, 1 mM NaF). Wash and lysis buffer of calyculin A treated samples contained additional 20 nM calyculin A. Lysates were sonicated to solubilize nucleocapsid-associated UL32-GFP before clearing the lysates for 20 min at 12,000 r.c.f. at 4 °C. For GFP-AP, GFP-trap agarose (gta-20, ChromoTek) was employed. Lysates were incubated with the agarose for 1 h, lysis buffer was used for the first two washing steps, and lysis buffer without detergent for the third and final washing step. Proteins were eluted by incubating the beads in a total volume of 0.2 ml 8 M guanidine hydrochloride at 95 °C under shaking. For HA-AP, a µMACS HA isolation kit (Miltenyi Biotec) was employed according to manufacturer instructions, with the following modifications. Lysates were incubated with the the magnetic microbeads for 1 h. After loading onto the microcolumns, lysis buffer was used for the first washing step, lysis buffer without detergent for the second and 25 mM Tris-HCl (pH 7.4) for the final washing step. Proteins were eluted by adding 200 µl 8 M guanidine hydrochloride that had been prewarmed to 95 °C.

Proteins were precipitated from the eluates by adding 1.8 ml LiChrosolv ethanol (Merck) and 1 µl GlycoBlue (Thermo Fisher). After incubation at 4 °C overnight, samples were centrifuged for 1 h at 4 °C, ethanol was decanted and the pellet was air-dried. Proteins were then resolved in digestion buffer (see above), and supplemented with trypsin and LysC at 1:25 and 1:100 enzyme-to-protein ratios (w/w), respectively. Digests were incubated overnight at 37 °C, subjected to C18 stage-tip desalting, followed by LC–MS analysis.

### Phosphopeptide enrichment

Peptides destined for phosphoproteome analysis were subjected to immobilized metal affinity chromatography (IMAC) enrichment using a ProPac IMAC-10 column (Thermo Fisher) on a 1260 Infinity II system (Agilent Technologies). A 30 min gradient was applied and the fraction corresponding to the phosphopeptides was collected, dried under speed vacuum and subjected to LC–MS analysis.

### LC–MS analysis

LC–MS analysis of crosslinked and strong cation exchange-fractionated peptides was performed using an UltiMate 3000 RSLC nano LC system coupled online to an Orbitrap Fusion Lumos mass spectrometer (Thermo Fisher). Reversed-phase separation was performed using a 50 cm analytical column (in-house packed with Poroshell 120 EC-C18, 2.7 µm, Agilent Technologies) with a 120 or 180 min gradient. Crosslink acquisition was performed using an LC–MS2 method. The following parameters were applied: MS resolution 120,000; MS2 resolution 60,000; charge state 4–8 enabled for MS2; stepped HCD energy 21, 27, 33.

LC–MS analysis of linear peptides (unmodified and phosphopeptides) was performed using a Dionex UltiMate 3000 system (Thermo Fisher) connected to a PepMap C18 trap column (0.075 ×50 mm, 3 μm particle size, 100 Å pore size; Thermo Fisher) and an in-house-packed C18 column (column material: Poroshell 120 EC-C18, 2.7 µm; Agilent Technologies) at 300 nl min^−1^ flow rate and 120–240 min gradient lengths. The MS1 scans were performed in the orbitrap using 120,000 resolution. Precursors were isolated with a 1.6 Da isolation window and fragmented by higher energy collision dissociation (HCD) with 30% normalized collision energy. The MS2 scans were acquired either in the ion trap or the orbitrap. For the ion trap, standard automatic gain control (AGC) target settings, an intensity threshold of 1 × 10^4^ (5 × 10^3^ for 240 min gradients) and maximum injection time of 50 ms were used. A 1 s cycle time was set between master scans. For MS2 acquisition in the orbitrap, we used standard AGC settings, an intensity threshold of 5 × 10^4^, 50 ms maximum injection time and a resolution of 15,000 for unmodified peptides or 30,000 for phosphopeptides. A 2 s cycle time was set between master scans. Xcalibur (Thermo Fisher) software was used for acquisition. A list of raw files for the different proteomic experiments is supplied in Supplementary Table 8.

### XL–MS data analysis

Peak lists (.mgf files) were generated in Proteome Discoverer (v.2.1) to convert each .raw file into one.mgf file containing HCD-MS2 data. The .mgf files were used as input to identify crosslinked peptides with a stand-alone search engine based on XlinkX v.2.0 (ref. ^69^). The following settings of XlinkX were used: MS ion mass tolerance, 10 ppm; MS2 ion mass tolerance, 20 ppm; fixed modification, Cys carbamidomethylation; variable modification, Met oxidation; enzymatic digestion, trypsin; allowed number of missed cleavages, 3; DSSO crosslinker, 158.0038 Da (short arm, 54.0106 Da; long arm, 85.9824 Da).

All MS2 spectra were searched against concatenated target-decoy databases generated on the basis of the virion proteome determined by bottom-up proteomics, containing 1,318 target sequence entries. Raw files from both biological replicates were searched, combined and crosslinks reported at 1% FDR at unique lysine–lysine connection level on the basis of a target-decoy calculation strategy using randomized decoys. Peptides were matched to proteins encoded by the respective genes and the gene name (HUGO official gene symbol or viral gene name) was then used for display in figures, tables and text. All identified crosslinks can be accessed in Supplementary Table 1. Quality-control analyses (related to Fig. 1c–e), clustering of viral proteins (related to Fig. 2a), analysis of scaffold indices (related to Fig. 2b), analysis of domain-specific interactions to UL32 (related to Fig. 2c,d) and coarse-grained modelling (Fig. 6) were performed with this set of crosslinks. All other analyses were based on the filtered set of crosslinks (see below), representing a high-confidence PPI map of the particle.

To supply an XL–MS PPI map with high density of relevant biological information, we applied further filtering criteria. For this filtering, we first checked whether crosslinks were identified in both replicates independently at 1% FDR. We required that crosslinks from abundant virion proteins (>10 copies) be identified in both replicates. When a crosslink involved a lower-abundance protein (that is, <10 copies), we required that the crosslink be identified in only one of the replicates. The rationale is that this balances crosslink confidence for highly abundant proteins with sensitivity for low-abundance proteins. Second, PPIs were only reported when there were two unique crosslinks identified, giving a higher-confidence set of PPIs. To retain only host proteins incorporated into the particle, host proteins that did not directly link to viral proteins were removed. The filtered set of crosslinks is available in Supplementary Table 2. The list of the corresponding PPIs together with evidence from existing data is available in Supplementary Table 3. The XL-based PPI network was generated on the basis of interprotein crosslinks from Supplementary Table 2 and visualized using edge-weighted spring-embedded layout^70^ in Cytoscape v.3.7.2. Edge weighting was based on the number of identified interlinks between protein pairs. Additional crosslink networks between selected proteins were visualized using xiNET^71^.

For clustering of viral proteins across virion (sub)layers, we used the full set of identified crosslinks at 1% FDR (Supplementary Table 1). Only crosslinks of viral proteins (intralinks and interlinks) were considered and viral proteins were excluded when (1) less than 10 of such crosslinks were identified or (2) the respective protein only formed intralinks. The crosslink count between any protein pair (‘interactor 1’ and ‘interactor 2’) was then divided by the total number of crosslinks for one of the proteins (‘interactor 1’) to yield PPI specificity values, according to equation (1). These calculations use concepts of graph theory, where the network is first partitioned into weighted subgraphs with path = 1 for each node (protein). Weights of the edges are represented by the number of crosslinks. For the nodes (A,B), the local connectivity is then divided by the centrality of one of the nodes (A).

1 $${\rm{PPIspec}}(A,B)={\rm{crosslink}\,{count}}(A,B)\div{\rm{crosslink}\,{count}}(A)$$

Correlation-based clustering (1-Pearson’s *R*) was performed on the resulting matrix using complete linkage and data were visualized in R (v.4.1.2.)/Rstudio (v.1.3.1093) using the heatmap.2 function from the gplots package. For calculating the scaffold index, we removed the PPI specificity values for interactions linking the same protein and summed up the PPI specificity values for each PPI of the interactor 2 proteins within the sublayers.

The circular heat map was generated using the circos.heatmap function in the circlize R package and clusters were based on Euclidean distances. Crosslinks of host proteins to viral proteins of the specific clusters (as obtained from the analysis in Fig. 2a, except for UL48A, which we considered as a nucleocapsid^8^) were *z*-scored by subtracting the mean and dividing by the standard deviation.

### Bottom-up proteomics data analysis

Raw data were analysed and processed using MaxQuant 1.6.2.6 software^72^. Search parameters included two missed cleavage sites, fixed cysteine carbamidomethyl modification and variable modifications including methionine oxidation, N-terminal protein acetylation. In addition, serine, threonine and tyrosine phosphorylations were searched as variable modifications for phosphoproteome analysis. Arg10 and Lys8 were set as labels where appropriate (double SILAC samples). The ‘second peptide’ option was enabled and peptide mass tolerance was 6 ppm for MS scans and 20 ppm for MS/MS scans. The software options ‘re-quantify’, intensity-based absolute quantification (‘iBAQ’) and ‘LFQ’ were enabled where appropriate. Database search was performed using Andromeda, the integrated MaxQuant search engine, against a protein database of HCMV strain TB40-BAC4 (ref. ^63^) and a Uniprot database of *Homo sapiens* proteins (downloaded 2020) with common contaminants. Peptides were matched to proteins encoded by the respective genes and the gene name (HUGO official gene symbol or viral gene name) was then used for display in figures, tables and text. FDR was estimated on the basis of target-decoy competition and set to 1% at peptide spectrum match, protein and modification site levels. For subsequent analysis, we used proteinGroups.txt, peptides.txt or Phospho (STY).txt (phosphoproteomics) MaxQuant output files with potential contaminants, reverse database hits and proteins only identified by (modification) site removed.

### Bottom-up proteomics data processing

For label-free AP–MS data, LFQ values from proteinGroups.txt were log_2_ transformed and two-sample *t*-test *P* values between experimental groups were calculated along with the average fold-change difference.

For SILAC-based quantification of virion-level protein differences, we used SILAC ratios as normalized by MaxQuant. For the corresponding phosphoproteome data, only sites with a localization probability >0.75 were considered (from the Phospho (STY).txt table). The positions of individual phosphosites as identified from this experiment were used to map phosphosites on the UL32 amino acid sequence (Fig. 4c). Phosphosite SILAC ratios were also corrected for the protein-level SILAC ratios (from proteinGroups.txt) as quantified from an analysis of the whole-virion proteome without IMAC enrichment. Phosphosites were excluded when the corresponding phosphopeptide contained residues mutated in the SILK/RVxF motifs of UL32. Then, protein-level corrected phosphosite ratios were averaged (quantification in both replicates required).

For comparing phosphosite and peptide abundances in label-free AP–MS samples, Maxquant output tables Phospho (STY).txt and peptides.txt were used, respectively. Phosphosites and peptide intensities for UL32 were summed up across the three replicates.

Enrichment levels comparing virions versus cells were calculated on the basis of log_2_-transformed LFQ intensities in MaxQuant output file proteinGroups.txt using Perseus software^73^. Therefore, the proteinGroups file was filtered by requiring at least four values in either virion or cell samples. Missing values were imputed on the basis of a normal distribution shrinked by a factor of 0.3 and downshifted by 1.8 standard deviations. *t*-tests, log_2_ differences and Spearman’s correlation coefficients (*⍴*) were calculated on the basis of these values.

### Absolute quantification of protein copy numbers

We absolutely quantified the copy numbers of host and viral proteins in purified particles on the basis of iBAQ values^74^. As calibrators, we used proteins with known copy numbers, associated with the nucleocapsid^8^ and portal complex^11^. Therefore, iBAQ values from MaxQuant output file proteinGroups.txt were extracted. We then used the following known copy numbers of HCMV proteins as calibrators for a linear regression analysis: UL93, UL104, 12 copies; UL77, 24 copies; UL46, 320 copies; UL85, 640 copies; UL86, UL48A, UL32, 960 copies^8,11^. Slope and offset were used to calculate unknown copy numbers of host and viral proteins according to equation (2). Including several proteins as reference points is expected to give more reliable copy number estimates than previously used one-point calibration of the intensity of UL86 with its copy number^16,18^.

2 $$\log_{10}({\rm{iBAQ}})={\rm{slope}}\times \log_{10}({\rm{copy}\,{number}})+{\rm{offset}}$$

Total protein copies were summed up for all identified proteins and divided by the volume of a sphere of 200 nM diameter to yield protein density. Protein mass was calculated by multiplying the total protein copy number with the average mass of a human protein (44 kDa), as previously calculated^37^.

### 14-3-3 binding-site prediction

We used the freely available 14-3-3pred tool (https://www.compbio.dundee.ac.uk/1433pred)^38^ to map putative 14-3-3 binding sites. Serines or threonines with a consensus score >0.6 were considered.

### Structure mapping and calculations

Mapping of crosslinks to cryoEM structures of UL55 (pdb 7kdp, 7kdd) was done using the PyMOL Molecular Graphics System (v.2.0). The shortest possible distances between individual chains were considered. One crosslink with distance greater than 40 Å in pre-fusion conformation could not be mapped to the post-fusion structural model due to missing coordinates at lysine residue 700.

Electrostatic surface charges of a nucleocapsid hexon (pdb 5vku) were calculated using the APBS Tools 2.1. plugin to Pymol and individual crosslinked lysines were highlighted as spheres.

### Tokoyasu staining

Human fibroblasts infected with HCMV-UL32-GFP were fixed with 4% formaldehyde for 120 min while shaking. After fixation, cells were pelleted, cryoprotected in 2.3 M sucrose and plunge-frozen on pins for Tokuyasu sectioning^75^. For immunogold labelling, ultrathin sections were collected on coated grids, blocked and co-stained with anti-GFP (1:250, 132005, Synaptic Systems), anti-pan 14-3-3 (1:50, clone H8, sc-1657, Santa Cruz) and anti-PP-1(1:50, anti-PP-1, clone E9, sc-7482, Santa Cruz) followed by secondary antibodies gp-12 nm gold (species: guinea pig) and ms-18 nm gold (species: mouse). After washing, sections were contrasted and covered with polyvinyl alcohol and tungsto-silicic acid hydrate. Stained ultrathin sections were examined with a Zeiss 902 transmission electron microscope at 80 kV and photographs were taken with a Morada G2 TEM camera.

### Negative-staining electron microscopy of HCMV particles

Cell culture supernatant was collected from infected fibroblasts at 6 d post infection as described in ‘Virion purification and crosslinking’ and either purified and/or crosslinked, or directly prepared for negative staining. Samples were fixed with glutaraldehyde at a concentration of 2% for 1 h at room temperature while shaking at 600 r.p.m. Afterwards, glutaraldehyde was removed by centrifuging samples in a TLS-55 rotor (Beckmann) at 30,000 r.p.m. (~77,000 × *g*) for 1 h and resuspending the resulting pellets in PBS. Samples were kept at 4 °C. Subsequently, samples were applied on 300 mesh copper grids coated with 1.8% pioloform and carbon. Grids were glow-discharged with an EMITECH K100X for 1 min. For each sample, a 4 µl drop was applied on the grid for 1 min before the solution was removed with a filter paper. After sample application, the grids were washed twice with ultra pure water (Fresenius, Ampuwa) and subsequently stained twice with 2% uranyl acetate for 1 min. Grids were imaged with a Zeiss 900 transmission electron microscope equipped with an 11 megapixel Olympus Morada G2 digital camera at an acceleration voltage of 80 kV. Around 10 to 12 pictures per condition were taken at a magnification of 30,000. For analysis of particle diameters, images were anonymized and analysed in Fiji (https://imagej.net/software/fiji/) image analysis software. Particles were manually picked by encircling vesicular structures with the oval selection mask tool and the major diameters of the resulting ovals were compared.

### Coarse-grained modelling

For coarse-grained modelling of the particle, we considered each protein molecule (except for UL32, viral glycoproteins, nucleocapsid proteins) as a single bead inside a sphere of 190 nm diameter (see Extended Data Fig. 1) enclosed by a coarse-grained lipid bilayer with realistic membrane kinetics^45^. The model integrates mean copy numbers of host and viral proteins (Supplementary Table 4) as bead counts. The sizes of the beads were approximated by their radii of gyration as calculated by the rgyr function in the bio3d package in R on the basis of AlphaFold2 models^48^. Host protein structural models (v.4) were downloaded from https://alphafold.ebi.ac.uk/. Viral structural models were downloaded from https://www.bosse-lab.org/herpesfolds/.

Due to its domain-dependent crosslinking pattern and the disordered state in its C-terminal region (AA 303–1,049), a more complex model of UL32 was required. We started with an all-atom model of UL32 from the AF2 structure and performed a molecular dynamics simulation using CHARMM 36m force field for 100 ns. We determined which residues tend to move together during the simulation (that is, have a highly correlated motion) on the basis of the fluctuations in pairwise distances between all residues. Residues grouped together in this manner constitute beads in the coarse-grained model of UL32. We approximated the segment-specific bead sizes on the basis of the expected radii of gyration of each residue group. We built the chain of beads representing one UL32 protein using harmonic bonds between subsequent beads, plus an angle-bending interaction between triplets of sequential beads to give the chain a non-vanishing bending rigidity. We additionally did a further subdivision of these beads while keeping the rigidity of the links intact (Extended Data Fig. 10c).

Extra- and intra-virion domains of glycoproteins were partitioned into smaller-sized beads on the basis of existing cryoEM structures (if available) or the respective predicted AF2 models. Positions of α-carbon atoms were clustered together using *k*-means and the size of individual beads was determined on the basis of the number of co-clustering α-carbon atoms (Extended Data Fig. 10b). This was done to accommodate (1) realistic binding of tegument proteins to the intra-virion domains and (2) accurate steric repulsions between extra-virion domains of glycoproteins, facilitating their sorting on the envelope. For the construction of the nucleocapsid, UL32 *n*-terminal domain was removed from the cryoEM structure (pdb 5vku) and the resulting capsid was partitioned into beads using the same approach used with glycoproteins. In both cases, structures were stabilized with elastic networks, which allows for some flexibility but prevents structural change. The UL32 *n*-terminal bead was then immobilized on this bead-approximated nucleocapsid, as supported by the corresponding cryoEM evidence^8^.

In addition, the model incorporates interaction restriction constants (IRC) to approximate the binding strength between individual beads. These were calculated for proteins contained in the virion XL map (Fig. 1f) and are based on heteromeric links (interlinks) extracted from Supplementary Table 1. Crosslinks of UL32 were grouped into 11 segments dependent on the crosslink position and the segment boundaries. Crosslinks for nucleocapsid proteins (UL32 *n*-terminal bead, UL93, UL48A, UL46, UL85, UL104, UL80) were then grouped and considered as one outer-capsid layer. Crosslinks to UL86 were considered as inner-capsid layer. In addition, we immobilized all UL48 beads on the outer-capsid layer as supported by cryoEM evidence^11^ and the observation that UL48 is not removed from virions by tegument stripping^3^.

For a species (bead or layer) pair *A* and *B*, IRC was calculated according to equation (3) as:

3 $$\begin{array}{l}{\rm{IRC}}\left(A,B\right)=\\\frac{{\rm{copy}\,{number}}\left(A\right)\times {\rm{PPIspec}}\left(A,B\right)+{\rm{copy}\,{number}}\left(B\right)\times {\rm{PPIspec}}\left(B,A\right)}{{\rm{copy}\,{number}}\left(A\right)+{\rm{copy}\,{number}}\left(B\right)},\end{array}$$

where PPIspec is calculated as in equation (1).

The IRCs were translated into binding free energies using a simple stochastic binding/unbiding model. All the beads in the model interacted via soft harmonic repulsions that model space exclusion between spherical beads. In addition, piecewise harmonic attractions were included between beads with non-zero IRCs. The well-depth of these bead-specific interactions were determined such that the resulting binding free energy matched the values obtained on the basis of IRCs.

Individual beads were arranged inside the particle in a self-avoiding random distribution. A random walk that avoids leaving the tegument region was used to model the initial configuration of UL32 chains emanating from the nucleocapsid.

Dynamics of all the beads in the model followed Brownian motion. Membrane particles possessed anisotropic diffusion coefficients derived on the basis of the hydrodynamic model of refs. ^45,76^. For beads in the UL32 chains, as well as all other proteins with individual beads, isotropic diffusion coefficients were determined on the Stokes–Einstein relation $\left(D=\frac{{k}_{\rm{B}}T}{6\pi \eta R}\right)$, in which *k*_B_ is the Boltzmann constant, *T* is the temperature, *η* is the viscosity of the medium and *R* is the hydrodynamic radius of the bead. We estimated the hydrodynamic radii from the radii of gyration using the geometric ratio of $\sqrt{3/5}\simeq 0.775$ between the latter and the former in a solid sphere. For glycoproteins as well as particles forming the nucleocapsid, we set the diffusion coefficient on the basis of a sphere enclosing the whole structure.

The whole model was initially simulated for 2,000 steps with a small timestep of 20 ps to allow for space-exclusion interactions to relax. This was followed by a simulation of ~58 μs total time, with a timestep of 120 ps. Equilibration was monitored by observing the mean radial distance (with respect to the centre point of the virus particle) of different species in the model. Sampling was performed when these values had settled to fluctuations about a well-defined mean.

Radial distances sampled in each frame for each bead type were gathered and organized into raw radial histograms. The histograms pertaining to each species (bead type) were transformed into radial probability densities according to equation (4) as:

4 $$p\left(r\right)=\frac{n\left(r\right)}{4\pi {r}^{2}\delta r}$$

where $n\left(r\right)$ denotes the fraction of beads positioned in the spherical shell formed between the radial distances of $r$ and $r+\delta r$. Thus, in spherical coordinates, this probability density integrates to unity for all bead types and properly highlights the localization of different species.

All simulations were done with in-house software developed in C++. The Python packages SciPy and Numpy were used for both the construction of bead models from protein structures and the analysis of the trajectories^77,78^. We used the Python package Biopython for processing cryoEM and AF2 structures^79^. The software package Visual Molecular Dynamics (VMD) was used for visualization of coarse-grained simulations^80^.

### Statistics and reproducibility

The analyses were based on viruses grown from human embryonic lung fibroblasts. XL-proteomics experiments, SILAC experiments and virion protein abundance measurements were conducted from biological duplicates. Label-free proteomics samples for cellular to virion protein enrichment levels were based on biological quadruplicates. Proteomics experiments (AP–MS) were conducted from biological triplicates. FACS experiments and growth curves were based on biological triplicate experiments and quantitative western blots were based on *n* = 3 or *n* = 4 technical replicates and *n* = 1 or *n* = 2 biological replicates. Other western blots were based on representative experiments of biological duplicates and results were additionally quantified by quantitative (SILAC) proteomics. Electron micrographs (133) from Tokoyasu staining of HCMV-UL32-GFP-infected cells and 50 additional micrographs of a specificity control (WT-UL32) were acquired from single biological replicates. No statistical methods were used to determine sample size, but our sample sizes are similar to those reported in previous publications^22,26,62^. No data were excluded from these analyses, experiments were not randomized and investigators were not blinded. Transmission electron microscopy images of viral particles were acquired from single experiments with blinding and randomization before analysis. In the case of parametric tests (*t*-tests), data were assumed to be normally distributed, but this was not formally tested.

### Reporting summary

Further information on research design is available in the Nature Portfolio Reporting Summary linked to this article.

## Supplementary information

Supplementary Information Supplementary Methods containing a step-by-step protocol for XL proteomics of virions.

Reporting Summary

Peer Review File

Supplementary Tables 1–8 Supplementary Tables 1–8.

Supplementary Video 1 Equilibrium sampling of the coarse-grained model. The viral envelope is rendered transparent, viral glycoproteins in red, tegument proteins in grey and host proteins in light pink.

Supplementary Video 2 Cross-sectional view of the virion model in equilibrium sampling, with tegument other than UL32 removed. See also Extended Data Fig. 10d.

## Data Availability

The mass spectrometry proteomics data have been deposited to the ProteomeXchange Consortium via the PRIDE^81^ partner repository with the dataset identifier PXD031911. A list of viral mutants and their amino acid exchanges is supplied in Supplementary Table 7. A list of raw files for each proteomic experiment is supplied in Supplementary Table 8. The simulation trajectory can be obtained from https://ftp.mi.fu-berlin.de/pub/cmb-data/hcmv_trajectories. Fasta files for proteomic searches were downloaded from Uniprot (https://www.uniprot.org/, human sequences) or GenBank (identifier: EF999921.1, viral sequences). AlphaFold2 models of human proteins (v.4) were downloaded from https://alphafold.ebi.ac.uk/. AlphaFold2 models of viral proteins were downloaded from https://www.bosse-lab.org/herpesfolds/. Source data are provided with this paper.

## Source data

Source Data Figs. 1–6, Source Data Extended Data Fig. 1 Statistical source data.
Source Data Fig. 4, Source Data Extended Data Figs. 5, 7 and 9 Statistical source data and unprocessed western blots.

## Code availability

The code used for analysing the mesoscopic simulations of the whole virion is available on GitHub at https://github.com/MohsenSadeghi/hcmv_mesoscopic_simulation. This analysis code contains information on importing and formatting of the trajectory data. The software used for mesoscopic simulations has a specific-purpose codebase developed in C++ with the aim of high performance and flexibility for the implementation of the membrane model used in this work. It does not possess a well-developed interface for general use and is therefore available from the corresponding author(s) upon reasonable request. R scripts for analysis of (proteomics) data is available at https://github.com/Bogdanob/VirionXLMS.

## References

[1] Liu, F. & Zhou, Z. H. in *Human Herpesviruses: Biology, Therapy, and Immunoprophylaxis* (eds Arvin, A. et al.) Ch. 3 (Cambridge Univ. Press, 2007). 21348071

[2] Connolly SA, Jardetzky TS, Longnecker R (2021). The structural basis of herpesvirus entry. Nat. Rev. Microbiol..

[3] Yu X (2011). Biochemical and structural characterization of the capsid-bound tegument proteins of human cytomegalovirus. J. Struct. Biol..

[4] Laine RF (2015). Structural analysis of herpes simplex virus by optical super-resolution imaging. Nat. Commun..

[5] Bohannon KP, Jun Y, Gross SP, Smith GA (2013). Differential protein partitioning within the herpesvirus tegument and envelope underlies a complex and variable virion architecture. Proc. Natl Acad. Sci. USA.

[6] Pegg CE (2021). Herpesviruses assimilate kinesin to produce motorized viral particles. Nature.

[7] Cavignac, Y. et al. The cellular proteins Grb2 and DDX3 are increased upon human cytomegalovirus infection and act in a proviral fashion. *PLOS ONE*10.1371/journal.pone.0131614 (2015). 10.1371/journal.pone.0131614 PMC4509573 26121620

[8] Yu, X., Jih, J., Jiang, J. & Hong Zhou, Z. Atomic structure of the human cytomegalovirus capsid with its securing tegument layer of pp150. *Science* **356**, eaam6892 (2017). 10.1126/science.aam6892 PMC5715728 28663444

[9] Draganova EB, Valentin J, Heldwein EE (2021). The ins and outs of herpesviral capsids: divergent structures and assembly mechanisms across the three subfamilies. Viruses.

[10] Rao VB, Fokine A, Fang Q (2021). The remarkable viral portal vertex: structure and a plausible model for mechanism. Curr. Opin. Virol..

[11] Li Z, Pang J, Dong L, Yu X (2021). Structural basis for genome packaging, retention, and ejection in human cytomegalovirus. Nat. Commun..

[12] Liu Y (2021). Prefusion structure of human cytomegalovirus glycoprotein B and structural basis for membrane fusion. Sci. Adv..

[13] Kschonsak M (2021). Structures of HCMV trimer reveal the basis for receptor recognition and cell entry. Cell.

[14] Leroy B, Gillet L, Vanderplasschen A, Wattiez R (2016). Structural proteomics of herpesviruses. Viruses.

[15] Rieder FJJ (2017). Human cytomegalovirus phosphoproteins are hypophosphorylated and intrinsically disordered. J. Gen. Virol..

[16] Reyda S (2014). The tegument protein pp65 of human cytomegalovirus acts as an optional scaffold protein that optimizes protein uploading into viral particles. J. Virol..

[17] Varnum SM (2004). Identification of proteins in human cytomegalovirus (HCMV) particles: the HCMV proteome. J. Virol..

[18] Couté Y (2020). Mass spectrometry-based characterization of the virion proteome, phosphoproteome, and associated kinase activity of human cytomegalovirus. Microorganisms.

[19] O’Reilly, F. J. & Rappsilber, J. Cross-linking mass spectrometry: methods and applications in structural, molecular and systems biology. *Nat. Struct. Mol. Biol*. **25**, 1000–1008 (2018). 10.1038/s41594-018-0147-0 30374081

[20] Gonzalez-Lozano MA (2020). Stitching the synapse: cross-linking mass spectrometry into resolving synaptic protein interactions. Sci. Adv..

[21] Schweppe DK (2017). Mitochondrial protein interactome elucidated by chemical cross-linking mass spectrometry. Proc. Natl Acad. Sci. USA.

[22] Liu F, Lössl P, Rabbitts BM, Balaban RS, Heck AJR (2018). The interactome of intact mitochondria by cross-linking mass spectrometry provides evidence for coexisting respiratory supercomplexes. Mol. Cell. Proteomics.

[23] Fasci D, van Ingen H, Scheltema RA, Heck AJR (2018). Histone interaction landscapes visualized by crosslinking mass spectrometry in intact cell nuclei. Mol. Cell. Proteomics.

[24] Mirzakhanyan Y, Gershon P (2019). The Vaccinia virion: filling the gap between atomic and ultrastructure. PLoS Pathog..

[25] Si Z (2018). Different functional states of fusion protein gB revealed on human cytomegalovirus by cryo electron tomography with Volta phase plate. PLoS Pathog..

[26] Nobre LV (2019). Human cytomegalovirus interactome analysis identifies degradation hubs, domain associations and viral protein functions. eLife.

[27] Owen DJ, Crump CM, Graham SC (2015). Tegument assembly and secondary envelopment of alphaherpesviruses. Viruses.

[28] Tullman JA, Harmon M-E, Delannoy M, Gibson W (2014). Recovery of an HMWP/hmwBP (pUL48/pUL47) complex from virions of human cytomegalovirus: subunit interactions, oligomer composition, and deubiquitylase activity. J. Virol..

[29] Schierling K, Stamminger T, Mertens T, Winkler M (2004). Human cytomegalovirus tegument proteins ppUL82 (pp71) and ppUL35 interact and cooperatively activate the major immediate-early enhancer. J. Virol..

[30] Kumar R, Cruz L, Sandhu PK, Buchkovich NJ (2020). UL88 mediates the incorporation of a subset of proteins into the virion tegument. J. Virol..

[31] Hensel GM (1996). Intracellular localization and expression of the human cytomegalovirus matrix phosphoprotein pp71 (ppUL82): evidence for its translocation into the nucleus. J. Gen. Virol..

[32] Fabits M (2020). The cytomegalovirus tegument protein UL35 antagonizes pattern recognition receptor-mediated type I IFN transcription. Microorganisms.

[33] Sanchez V, Sztul E, Britt WJ (2000). Human cytomegalovirus pp28 (UL99) localizes to a cytoplasmic compartment which overlaps the endoplasmic reticulum-golgi-intermediate compartment. J. Virol..

[34] Dietz AN, Villinger C, Becker S, Frick M, von Einem J (2018). A tyrosine-based trafficking motif of the tegument protein pUL71 is crucial for human cytomegalovirus secondary envelopment. J. Virol..

[35] Chevillotte M (2009). Major tegument protein pp65 of human cytomegalovirus is required for the incorporation of pUL69 and pUL97 into the virus particle and for viral growth in macrophages. J. Virol..

[36] Dai X (2013). The smallest capsid protein mediates binding of the essential tegument protein pp150 to stabilize DNA-containing capsids in human cytomegalovirus. PLoS Pathog..

[37] Milo R (2013). What is the total number of protein molecules per cell volume? A call to rethink some published values. Bioessays.

[38] Madeira F (2015). 14-3-3-Pred: improved methods to predict 14-3-3-binding phosphopeptides. Bioinformatics.

[39] Michelson, S. et al. Human cytomegalovirus carries serine/threonine protein phosphatases PP1 and a host-cell derived PP2A. *J. Virol.*10.1128/jvi.70.3.1415-1423.1996 (1996). 10.1128/jvi.70.3.1415-1423.1996 PMC189961 8627658

[40] Heroes, E. et al. The PP1 binding code: a molecular-lego strategy that governs specificity. *FEBS J.*10.1111/j.1742-4658.2012.08547.x (2013). 10.1111/j.1742-4658.2012.08547.x 22360570

[41] Guard SE (2019). The nuclear interactome of DYRK1A reveals a functional role in DNA damage repair. Sci. Rep..

[42] Glenewinkel F (2016). The adaptor protein DCAF7 mediates the interaction of the adenovirus E1A oncoprotein with the protein kinases DYRK1A and HIPK2. Sci. Rep..

[43] Bogdanow B, Phan QV, Wiebusch L (2021). Emerging mechanisms of G/S cell cycle control by human and mouse cytomegaloviruses. mBio.

[44] Bogdanow B (2013). Human cytomegalovirus tegument protein pp150 acts as a cyclin A2-CDK-dependent sensor of the host cell cycle and differentiation state. Proc. Natl Acad. Sci. USA.

[45] Sadeghi M, Noé F (2020). Large-scale simulation of biomembranes incorporating realistic kinetics into coarse-grained models. Nat. Commun..

[46] Sadeghi M, Weikl TR, Noé F (2018). Particle-based membrane model for mesoscopic simulation of cellular dynamics. J. Chem. Phys..

[47] Sadeghi M, Noé F (2021). Thermodynamics and kinetics of aggregation of flexible peripheral membrane proteins. J. Phys. Chem. Lett..

[48] Jumper J (2021). Highly accurate protein structure prediction with AlphaFold. Nature.

[49] Vollmer B (2020). The prefusion structure of herpes simplex virus glycoprotein B. Sci. Adv..

[50] Lee J-H, Kalejta RF (2019). Human cytomegalovirus enters the primary CD34 hematopoietic progenitor cells where it establishes latency by macropinocytosis. J. Virol..

[51] Tandon R, Mocarski ES (2008). Control of cytoplasmic maturation events by cytomegalovirus tegument protein pp150. J. Virol..

[52] AuCoin DP, Smith GB, Meiering CD, Mocarski ES (2006). Betaherpesvirus-conserved cytomegalovirus tegument protein ppUL32 (pp150) controls cytoplasmic events during virion maturation. J. Virol..

[53] He B, Gross M, Roizman B (1997). The gamma(1)34.5 protein of herpes simplex virus 1 complexes with protein phosphatase 1alpha to dephosphorylate the alpha subunit of the eukaryotic translation initiation factor 2 and preclude the shutoff of protein synthesis by double-stranded RNA-activated protein kinase. Proc. Natl Acad. Sci. USA.

[54] Benedyk TH (2021). pUL21 is a viral phosphatase adaptor that promotes herpes simplex virus replication and spread. PLoS Pathog..

[55] Davis ME (2014). Antagonism of the phosphatase PP1 by the measles virus V protein is required for innate immune escape of MDA5. Cell Host Microbe.

[56] Nekhai, S., Ammosova, T., Charles, S. & Jeang, K. Regulation of hiv‐1 transcription by protein phosphatase 1. *FASEB J.*10.1096/fasebj.21.6.a1033-b (2007).

[57] Stecher C (2021). Protein phosphatase 1 regulates human cytomegalovirus protein translation by restraining AMPK signaling. Front. Microbiol..

[58] Dharan A (2017). Bicaudal D2 facilitates the cytoplasmic trafficking and nuclear import of HIV-1 genomes during infection. Proc. Natl Acad. Sci. USA.

[59] Liu, Y.-T., Strugatsky, D., Liu, W. & Hong Zhou, Z. Structure of human cytomegalovirus virion reveals host tRNA binding to capsid-associated tegument protein pp150. *Nat. Commun.* **12**, 5513 (2021). 10.1038/s41467-021-25791-1 PMC8448752 34535641

[60] Bresnahan WA, Shenk T (2000). A subset of viral transcripts packaged within human cytomegalovirus particles. Science.

[61] Chandramouli, S. et al. Structural basis for potent antibody-mediated neutralization of human cytomegalovirus. *Sci. Immunol.* **2**, eaan1457 (2017). 10.1126/sciimmunol.aan1457 28783665

[62] Zydek M, Hagemeier C, Wiebusch L (2010). Cyclin-dependent kinase activity controls the onset of the HCMV lytic cycle. PLoS Pathog..

[63] Sinzger C (2008). Cloning and sequencing of a highly productive, endotheliotropic virus strain derived from human cytomegalovirus TB40/E. J. Gen. Virol..

[64] Eifler M (2014). PUL21a-Cyclin A2 interaction is required to protect human cytomegalovirus-infected cells from the deleterious consequences of mitotic entry. PLoS Pathog..

[65] Tischer BK, Smith GA, Osterrieder N (2010). En passant mutagenesis: a two step markerless red recombination system. Methods Mol. Biol..

[66] Zimmermann C (2018). The abundant tegument protein pUL25 of human cytomegalovirus prevents proteasomal degradation of pUL26 and supports its suppression of ISGylation. J. Virol..

[67] Wessel D, Flügge UI (1984). A method for the quantitative recovery of protein in dilute solution in the presence of detergents and lipids. Anal. Biochem..

[68] Bogdanow B (2020). Cross-regulation of viral kinases with cyclin A secures shutoff of host DNA synthesis. Nat. Commun..

[69] Liu F, Lössl P, Scheltema R, Viner R, Heck AJR (2017). Optimized fragmentation schemes and data analysis strategies for proteome-wide cross-link identification. Nat. Commun..

[70] Kamada, T. & Kawai, S. An algorithm for drawing general undirected graphs. *Inf. Process. Lett.* **31**, 7–15 (1989).

[71] Combe CW, Fischer L, Rappsilber J (2015). xiNET: cross-link network maps with residue resolution. Mol. Cell. Proteomics.

[72] Cox, J. & Mann, M. MaxQuant enables high peptide identification rates, individualized p.p.b.-range mass accuracies and proteome-wide protein quantification. *Nat. Biotechnol.* **26**, 1367–1372 (2008). 10.1038/nbt.1511 19029910

[73] Tyanova S (2016). The Perseus computational platform for comprehensive analysis of (prote)omics data. Nat. Methods.

[74] Schwanhäusser B (2011). Global quantification of mammalian gene expression control. Nature.

[75] Tokuyasu, K. T. A technique for ultracryotomy of cell suspensions and tissues. *J. Cell Biol.* **57**, 551–565 (1973). 10.1083/jcb.57.2.551 PMC2108989 4121290

[76] Sadeghi M, Noé F (2021). Hydrodynamic coupling for particle-based solvent-free membrane models. J. Chem. Phys..

[77] Virtanen P (2020). SciPy 1.0: fundamental algorithms for scientific computing in Python. Nat. Methods.

[78] Harris CR (2020). Array programming with NumPy. Nature.

[79] Cock PJA (2009). Biopython: freely available Python tools for computational molecular biology and bioinformatics. Bioinformatics.

[80] VMD: visual molecular dynamics. *J. Mol. Graph*. **14**, 33–38 (1996). 10.1016/0263-7855(96)00018-5 8744570

[81] Perez-Riverol Y (2022). The PRIDE database resources in 2022: a hub for mass spectrometry-based proteomics evidences. Nucleic Acids Res..

