# Supplementary material for: Spatially resolved protein map of intact human cytomegalovirus virions
Source: Nat Microbiol. 2023 Aug 7;8(9):1732–47. doi: 10.1038/s41564-023-01433-8 (PMC10465357; doi:10.1038/s41564-023-01433-8)
Supplement: Supplementary file 1 — Supplementary Methods containing a step-by-step protocol for XL proteomics of virions. [file 41564_2023_1433_MOESM1_ESM.pdf]

nature microbiology

Article

https://doi.org/10.1038/s41564-023-01433-8

Spatially resolved protein map of intact human cytomegalovirus virions

In the format provided by the authors and unedited

Supplementary Methods

Step-by-step protocol for preparation of XL-proteomics samples from cross-linked HCMV particles

Materials:

- 15 cm cell culture dish
- 1.5 mL/2 mL reaction tube, Eppendorf
- 50mL centrifugation tube, Falcon
- SW-28 rotor, Beckmann
- SW-40 rotor, Beckmann
- SW-60 rotor, Beckmann
- TLS-55 rotor, Beckmann
- ultracentrifugation tube 38.5 mL, Open-Top Thinwall Ultra-Clear Tube, 25 x 89mm, Beckmann
- ultracentrifugation tube 14 mL, Open-Top Thinwall Ultra-Clear Tube, 14 x 95mm, Beckmann
- ultracentrifugation tube 4 mL, Open-Top Thinwall Ultra-Clear Tube, 11 x 60mm, Beckmann
- ultracentrifugation tube 2.2 mL, Open-Top Thinwall Ultra-Clear Tube, 11 x 34mm, Beckmann
- protein deglycosylation kit (Protein Deglycosylation Mix II), NEB
- Sep-Pak C8 1 cc Vac Cartridge, Waters
- PolySULFOETHYL-A column (100 × 2.1 mm, 3 µm particles), PolyLC inc.
- Pierce™Quantitative Colorimetric Peptide Assay, Thermo Fisher Scientific

Chemicals:

- methanol (MS grade), VWR International
- disuccinimidyl sulfoxide (DSSO), Cayman Chemical Company
- water-free dimethy sulfoxide (DMSO), Sigma-Aldrich
- chloroform (laboratory reagent grade), Fisher Scientific
- formic acid (FA), VWR International
- triethylammonium bicarbonate (TEAB, 1.0 M), Sigma-Aldrich
- Triton X-100, Carl Roth
- tris(2-carboxyethyl)phosphine (TCEP), Sigma-Aldrich
- 2-chloracetamide (CAA), Aldrich Chemistry
- sodium chloride (NaCl), Carl Roth
- acetonitrile (ACN, LC-MS), Fisher Scientific
- hydrochloric acid (HCl, TitriPUR®), Merck
- tris(hydroxymethyl)aminomethane (Tris), Carl Roth
- urea (BioScience grade), Carl Roth
- EDTA-free protease inhibitor mix (Pierce™), Thermo Scientific

Buffers and Media:

- phosphate buffered saline (PBS), Gibco

- Human embryonic lung fibroblasts (Fi301) medium
    - Eagle's minimum essential medium (EMEM), Lonza
    - 10 % (v/v) FCS, Sigma-Aldrich
    - 0.15 % (w/v) sodium bicarbonate (Gibco)
    - 1 mM sodium pyruvate (Gibco)
    - 0.1 mM non-essential amino acids (Lonza)
    - 2 mM GlutaMAX™ (Gibco)
    - 50 µg/ml gentamicin (Corning)
- lysis buffer
    - 50 mM triethylammonium bicarbonate (TEAB)
    - 1% (v/v) Triton X-100
    - 5 mM tris(2-carboxyethyl)phosphine (TCEP)
    - 30 mM 2-chloracetamide (CAA)
    - 1 pill/10 mL EDTA-free protease inhibitor mix
    - pH adjusted to 8.0
- digestion buffer
    - 50 mM triethylammonium bicarbonate (TEAB)
    - 1% (m/v) sodium deoxycholate (SDC)
    - 5 mM tris(2-carboxyethyl)phosphine (TCEP)
    - 30 mM 2-chloracetamide (CAA)
    - pH adjusted to 8.0
- Tris-HCl
    - tris(hydroxymethyl)aminomethane (Tris)
    - pH adjusted with hydrochloric acid (HCl) to 8.0
- Sep-Pak buffer A
    - 0.1 % (v/v) formic acid
- Sep-Pak buffer B
    - 0.1 % (v/v) formic acid
    - 60 % (v/v) acetonitrile (ACN)
- strong cation exchange (SCX) buffer A
    - 0.05 % (v/v) formic acid
    - 20 % (v/v) acetonitrile
- strong cation exchange (SCX) buffer B
    - 0.05 % (v/v) formic acid
    - 20 % (v/v) acetonitrile
    - 0.5 M sodium chloride (NaCl)

all buffers have been prepared with Milli-Q (18 MΩ) water

Instruments:

- XL-80 Ultracentrifuge, Beckmann
- Optima TLX Ultracentrifuge, Beckmann
- Centrifuge 5427 R, Eppendorf
- Agilent 1260 Infinity II UPLC system
- SpeedVac Savant SPD1010, Thermo Scientific
- Bioruptor® Pico sonication device, Diagenode

Enzymes:

- trypsin / EDTA 0.025% solution, Lonza

- trypsin (MS approved), Serva Electrophoresis
- lysyl endopeptidase (LysC, MS grade), FUJIFILM Wako Pure Chemical Corporation
- Benzonase Nuclease HC, EMD Millipore Corp.

Cells and virus:

- Human embryonic lung fibroblasts (Fi301), gifted from Institute of Virology Charité, Berlin, Germany
- HCMV TB40-BAC4, gifted from Christian Sinzinger, Ulm, Germany

Procedure

Purification and cross-linking of HCMV particles

- transfer infectious cell culture supernatant at 6 days post infection from confluent 15 cm cell culture plates to 50 mL polypropylene tubes
- centrifuge at 1,500 * g for 10 min and proceed with supernatant by decantation determine virus titer according to [1]
- proceed with ca. 2E+10 infectious units per replicate
- centrifuge respective volume of virus containing cell culture supernatant in 38.5 mL ultracentrifugation tubes (Beckmann) in a SW-28 rotor (Beckmann) at 25,000 rpm and 10 °C for 1 h (~112,500 x g)
- a pellet should build up at the bottom of the tube
- resuspend the pellet in 20 mL of PBS
- repeat previous centrifugation step
- resuspend pellet in 1-2 pellet volumes (ca. 100 µl) PBS, transfer into 1.5 mL reaction tube
- resuspend 1 µg solid, crystalline DSSO in 25 µl water-free DMSO, resulting in a 0.1 M stock solution
- add 2.5 µl of DSSO solution to virion resuspension and shake at RT at 1000 rpm for 30 min
- repeat addition of 2.5 µl DSSO and shake again at 1000 rpm for 30 min
- quench cross-linking reaction by addition of 50 mM Tris-HCl (pH 8.0), continue with shaking at 1000 rpm for 20 min
- in a 14 mL ultracentrifugation tube prepare glycerol-tartrate gradient as described [2] . Prepare several gradients in order not to overload.
- carefully add cross-linked virion suspension on top of density gradient
- centrifuge in a SW-40 rotor at 25,000 rpm for 1 h at 10 °C with brakes set at lowest deceleration (~111,000 x g)
- virions form a sharp band below a diffuse upper band
- take off virion band by laterally piercing the ultracentrifugation tube with a 1 mL syringe with a 1.2*40 mm needle
- in a 4mL ultracentrifugation tube mix the extracted virion fraction with 2 mL of PBS by inverting the tube multiple times to dissolve remaining gradient material
- centrifuge in a SW-60 rotor (Beckmann) for 1 h at 30,000 rpm at 10 °C (~84,000 x g)
- remove supernatant. The virion pellet appears slightly bluish.
- resuspend virion pellet in 1 mL PBS and transfer into 2.2 mL ultracentrifugation tube
- centrifuge in a TLS-55 rotor (Beckmann) at 35,000 rpm for 1 h at 10°C (77,000 x g)
- remove supernatant

- pellets can be stored at -80 °C

Sample preparation for XL-MS

- resuspend virion pellet in 80 µl PBS
- perform deglycosyltaion of surface proteins using deglycosylation kit (Protein Deglycosylation Mix II, NEB) under denaturating conditions
    - add 10 µl Deglycosylation Mix Buffer 2
    - incubate at 75 °C for 10 minutes, cool down to RT
    - add 10 µl Protein Deglycosylation Mix II enzyme
    - incubate for 30 min at RT
    - incubate for 1 h at 37°C while shaking at 600 rpm
- add 900 µl freshly prepared lysis buffer
- add 700 units of Benzonase
- incubate on ice for 30 min
- incubate in Bioruptor sonicator for 45 min (30 s on, 30 s off) at 4 °C to support full lysis
- perform methanol/chloroform precipitation according to [3]
    - add 4 lysate volumes of methanol, vortex
    - add 1 lysate volumes of chloroform, vortex
    - add 3 lysate volumes of Milli-Q water, vortex
    - centrifuge at 13,000*g for 15 min
    - remove upper liquid layer
    - add 3 lysate volumes of methanol, vortex
    - discard supernatant
- air-dry protein pellets for 10 min
- resuspend protein pellet in 500 µl digestion buffer and incubate at 37 °C while shaking at 1000 rpm
- add 10 µg trypsin and 2.5 µg LysC
- incubate over-night at 37 °C
- stop digestion by adding FA to a concentration of 1 % (v/v)
- centrifuge at 10,000*g for 10 min at RT
- continue with desalting of peptide containing supernatant using Sep-Pak C8 1 cc Vac Cartridge, Waters:
    - add 1 mL of methanol to Sep-Pak column, allow to empty by gravity-flow
    - add 1 mL of Sep-Pak buffer B to Sep-Pak column, allow to empty by gravity-flow
    - add 1 mL of Sep-Pak buffer A to Sep-Pak column, allow to empty by
    - gravity-flow, repeat step once
    - apply digested sample to Sep-Pak column, allow to empty by gravity-flow
    - add 1 mL of Sep-Pak buffer A to Sep-Pak column, allow to empty by gravity-flow, repeat step once
    - allow the column to dry completely
    - apply 400 µl buffer B and collect flow-through in 2 mL reaction tube
- determine peptide yield with Pierce™Quantitative Colorimetric Peptide Assay
- yield should be 200-300 µg of peptides per replicate
- dry completely in SpeedVac
- resuspend in 90 µl 1 % (v/v) FA

- fractionate peptide mixture by strong cation exchange exchange chromatography with a PolySULFOETHYL A ™ (100 × 2.1 mm, 3 µm particles) column connected to an Agilent 1260 Infinity II HPLC system
- separate peptides by a 95 min gradient, ranging from buffer A (0.05 % (v/v) FA, 20 % (v/v) ACN) to buffer B (0.05 % (v/v) FA, 20 % (v/v) ACN, 0.5 M NaCl) using the following gradient: 0–0.01 min (0–2% B); 0.01–8.01 min (2–3% B); 8.01–14.01 min (3–8% B); 14.01–28 min (8–20% B); 28–48 min (20–40% B); 48–68 min (40–90% B); 68–74 min (90% B); 74–95 min (0% B)
- collect fractions every 60 s
- desalt individual fractions by c8 stage tipping as described [4]
- store dried stage tips at 4 °C and elute peptides directly prior to mass spectrometric analysis with 40 µl Sep-Pak buffer B

To achieve a high-confidence XL-MS network, we recommend two independent replicates.

References

1. Eifler, M. *et al.* PUL21a-Cyclin A2 interaction is required to protect human cytomegalovirus-infected cells from the deleterious consequences of mitotic entry. *PLoS Pathog.* **10**, e1004514 (2014).
2. Zimmermann, C. *et al.* The Abundant Tegument Protein pUL25 of Human Cytomegalovirus Prevents Proteasomal Degradation of pUL26 and Supports Its Suppression of ISGylation. *J. Virol.* **92**, (2018).
3. Wessel, D. & Flügge, U. I. A method for the quantitative recovery of protein in dilute solution in the presence of detergents and lipids. *Anal. Biochem.* **138**, 141–143 (1984).
4. Rappsilber, J., Mann, M. & Ishihama, Y. Protocol for micro-purification, enrichment, pre-fractionation and storage of peptides for proteomics using StageTips. *Nat. Protoc.* **2**, 1896–1906 (2007).
