# Supplementary material for: Spatially resolved protein map of intact human cytomegalovirus virions
Source: Nat Microbiol. 2023 Aug 7;8(9):1732–47. doi: 10.1038/s41564-023-01433-8 (PMC10465357; doi:10.1038/s41564-023-01433-8)
Supplement: Supplementary file 2 — Reporting Summary [file 41564_2023_1433_MOESM2_ESM.pdf]

nature portfolio

Corresponding author(s): Boris Bogdanow, Mohsen Sadeghi, Lüder Wiebusch, Fan Liu

Last updated by author(s): Jun 6, 2023

Reporting Summary

Nature Portfolio wishes to improve the reproducibility of the work that we publish. This form provides structure for consistency and transparency in reporting. For further information on Nature Portfolio policies, see our Editorial Policies and the Editorial Policy Checklist.

Statistics

For all statistical analyses, confirm that the following items are present in the figure legend, table legend, main text, or Methods section.

n/a	Confirmed	
☐	☒	The exact sample size (*n*) for each experimental group/condition, given as a discrete number and unit of measurement
☐	☒	A statement on whether measurements were taken from distinct samples or whether the same sample was measured repeatedly
☐	☒	The statistical test(s) used AND whether they are one- or two-sided<br>*Only common tests should be described solely by name; describe more complex techniques in the Methods section.*
☒	☐	A description of all covariates tested
☐	☒	A description of any assumptions or corrections, such as tests of normality and adjustment for multiple comparisons
☐	☒	A full description of the statistical parameters including central tendency (e.g. means) or other basic estimates (e.g. regression coefficient) AND variation (e.g. standard deviation) or associated estimates of uncertainty (e.g. confidence intervals)
☐	☒	For null hypothesis testing, the test statistic (e.g. *F*, *t*, *r*) with confidence intervals, effect sizes, degrees of freedom and *P* value noted<br>*Give P values as exact values whenever suitable.*
☒	☐	For Bayesian analysis, information on the choice of priors and Markov chain Monte Carlo settings
☒	☐	For hierarchical and complex designs, identification of the appropriate level for tests and full reporting of outcomes
☐	☒	Estimates of effect sizes (e.g. Cohen's *d*, Pearson's *r*), indicating how they were calculated

Our web collection on statistics for biologists contains articles on many of the points above.

Software and code

Policy information about availability of computer code

Data collection	FACSDiva version 6.1.2<br>Thermo Scientific Xcalibur (v4.4. to v4.6.)<br>14-3-3 pred (https://www.compbio.dundee.ac.uk/1433pred)
Data analysis	FACSDiva version 6.1.2<br>XlinkX v.2.0 (Liu et al., 2017,Nat Commun)<br>XiNet (http://crosslinkviewer.org/index.php)<br>R (v4.1.2.)<br>Rstudio (v.1.3.1093)<br>MaxQuant (v.1.6.2.6)<br>CytoScape(v3.7.2) (https://cytoscape.org/)<br>etBrains CLion C++ IDE + GNU Compiler Collection (GCC) (v11.2.0)<br>Particle-based Membrane Simulation Software (Sadeghi & Noé, 2020 Nat Commun and Sadeghi et al., 2018, J. Chem. Phys.)<br>Python (v3.11) + Jupyter Notebook (v6.5.4)<br>Python packages SciPy (https://github.com/scipy/scipy), Numpy (https://github.com/numpy/numpy) , Biopython (https://github.com/biopython/biopython)<br>R packages circlize (https://github.com/jokergoo/circlize, gplots (https://github.com/talgalili/gplots), bio3d (http://thegrantlab.org/bio3d/)<br>Visual Molecular Dynamics (VMD) (v1.9.4a55)<br>Perseus (v1.6.7.0)

MaxQuant 1.6.2.6.
Fiji image analysis software (https://imagej.net/software/fiji/)
CHARMM36m (http://mackerell.umaryland.edu/charmm_ff.shtml)

For manuscripts utilizing custom algorithms or software that are central to the research but not yet described in published literature, software must be made available to editors and reviewers. We strongly encourage code deposition in a community repository (e.g. GitHub). See the Nature Portfolio guidelines for submitting code & software for further information.

Data

Policy information about availability of data

All manuscripts must include a data availability statement. This statement should provide the following information, where applicable:

- Accession codes, unique identifiers, or web links for publicly available datasets
- A description of any restrictions on data availability
- For clinical datasets or third party data, please ensure that the statement adheres to our policy

The mass spectrometry proteomics data have been deposited to the ProteomeXchange Consortium via the PRIDE partner repository with the dataset identifier PXD031911. A list of viral mutants and their amino acid exchanges is supplied in Supplementary Table 7. A list of raw-files for each proteomic experiment is supplied in Supplementary Table 8. The simulation trajectory can be obtained from https://ftp.mi.fu-berlin.de/pub/cmb-data/hcmv_trajectories. Fasta files for proteomic searches were downloaded from Uniprot (https://www.uniprot.org/, human sequences) or GenBank (identifier: EF999921.1, viral sequences). AlphaFold2 models of human proteins (v4) were downloaded from https://alphafold.ebi.ac.uk/. AlphaFold2 models of viral proteins were downloaded from https://www.bosse-lab.org/herpesfolds/.

Human research participants

Policy information about studies involving human research participants and Sex and Gender in Research.

Reporting on sex and gender	No human research participants were involved.
Population characteristics	No human research participants were involved.
Recruitment	No human research participants were recruited.
Ethics oversight	No committee approved the study protocol.

Note that full information on the approval of the study protocol must also be provided in the manuscript.

Field-specific reporting

Please select the one below that is the best fit for your research. If you are not sure, read the appropriate sections before making your selection.

☒ Life sciences ☐ Behavioural & social sciences ☐ Ecological, evolutionary & environmental sciences

For a reference copy of the document with all sections, see nature.com/documents/nr-reporting-summary-flat.pdf

Life sciences study design

All studies must disclose on these points even when the disclosure is negative.

Sample size	No statistical methods were used to predetermine sample sizes. Sample sizes were chosen based on prior knowledge of the intrinsic variability of individual experimental methods, as performed in previous studies. Our sample sizes are similar to those reported in previous publications (Zydek et al., Plos Pathog., 2010; Liu et al., 2018, MCP; Bogdanow et al., 2020, Nat Commun; Bogdanow et al., Nat Commun, 2019)
Data exclusions	No data were excluded
Replication	Western blot experiments were replicated at least twice. Sample sizes were n=2 for XL-MS and SILAC virion (phospho)proteomics (including label-swaps). n=3 for AP-MS, growth curves and FACS experiments. n=4 for label-free comparison of viral and cellular proteomes. n=2 for absolute quantification of viral protein abundance (in technical duplicates or triplicates). Replications were successful. Electron microscopy images were acquired from single biological replicates.
Randomization	Samples were not randomized, as no experimental groups were used in this study
Blinding	Investigators were not blinded, as the aim of the study was not based on any prior hypotheses (Large scale XL-MS of the particles). Furthermore, the read-out of many experiments (LC-MS/MS, FACS (e.g. immunotitrations)) were not based on subjective assessments. Western blot experiments were not blinded as a a single researcher performed all experimental/analysis steps. Blinding was performed to analyze virion preparations from negative stainings.

Reporting for specific materials, systems and methods

We require information from authors about some types of materials, experimental systems and methods used in many studies. Here, indicate whether each material, system or method listed is relevant to your study. If you are not sure if a list item applies to your research, read the appropriate section before selecting a response.

Materials & experimental systems

n/a	Involved in the study
☐	☒ Antibodies
☐	☒ Eukaryotic cell lines
☒	☐ Palaeontology and archaeology
☒	☐ Animals and other organisms
☒	☐ Clinical data
☒	☐ Dual use research of concern

Methods

n/a	Involved in the study
☒	☐ ChIP-seq
☐	☒ Flow cytometry
☒	☐ MRI-based neuroimaging

Antibodies

Antibodies used

anti-UL32 (clone XP1, provided by Bodo Plachter)
anti-PP1 (clone E9, sc-7482, Santa Cruz)
anti-14-3-3 (clone H8, sc-1657, Santa Cruz)
anit-UL99 (clone CH19, sc-69749)
anti-IE1/2 Alexa Fluor 488 conjgated (clone 8B1.2, MAB810X, Merck)
anti-GFP (132005, Synaptic Systems, guinea pig)
anti-RPS6 (clone 5G10, #2217, Cell signaling Technology)
anti-UL85 (rabbit polyclonal, provided by Wade Gibson)
anti-UL82 (clone 2H10-9, provided by Tom Shenk)
anti-UL83 (clone CH12, sc-56976, Santa Cruz)
anti-UL122/UL123 (clone E13, Biomerieux, cat# 11-003)

Validation

The anti-UL32 antibody has been validated using Immunoblotting, Immunofluorescence and ELISA against prokaryotic and viral source of the antigen by Scholl et al., 1988, J Gen Virol
The anti-PP1 antibody was validated by the manufacturer in Immunoblotting (https://www.scbt.com/p/pp1-antibody-e-9). Results from the Immunoblotting experiments from this antibody also validated by mass spectrometry. See Extended Data Fig 6b, Fig. 4e.
The anti-14-3-3 antibody was validated by the manufacturer in Immunoblotting (https://www.scbt.com/p/pan-14-3-3-antibody-h-8?requestFrom=search). Results from the Immunoblotting experiments from this antibody also validated by mass spectrometry. See Extended Data Fig. 5c-e.
The anti-UL99 antibody was validated previously in Immunoblotting experiments (e.g. Weisbach et al., 2017, PLoS Pathog.) with no detection of the viral antigen in uninfected cells and detection of the antigen with true-late kinetics in infected cells.
The anti-IE1/2 antibody is validated in this manuscript, showing no signal in uninfected cells (see Extended Data Figure 7c).
The anti-GFP antibody has been validated by the manufacturer in WB and IHC (https://sysy.com/product/132005) and was validated in EM by the absence of specific signal in WT-HCMV infected cells in contrast to Nucleocapsid associated signal in HCMV-pp150-GFP infected cells.
The anti-RPS6 antibody has been validated by the manufacturer for the detection of human, rodent and monkey RPS6 in immunoblotting, immunofluorescence and immunohistochemistry approaches (https://www.cellsignal.com/products/primary-antibodies/s6-ribosomal-protein-5g10-rabbit-mab/2217). The outstanding specificity and performance of this RPS6 antibody clone has been documented in more than 2000 scientific publications.
The anti-UL85 antibody has been validated using immunoblotting analysis of purified HCMV particles and infected cell extracts. It shows no signal in uninfected cells. It recognizes the C-terminal 15 amino acids of UL85. See Gibson et al., 1996, J. Virol., Borst et al., 2022, mBio, doi: 10.1128/mbio.01007-22.
The anti-UL82 antibody has been validated using immunoblotting in infected cell extracts. It shows no signal in uninfected cells. See vanDeusen et al., 2015, J virol; Kalejta et al., 2003, Molecular and Cellular Biology.
The anti-UL83 antibody has been validated using immunoblotting analysis of infected cells. It shows no signal in uninfected cells and detects the antigen with early-late kinetics. (Weisbach et al., 2017, Plos Pathog; Eifler et al., Plos Pathog., 2014)
The anti-UL122/UL123 antibody has been validated for immunofluorescence and immunoblotting. It detects an immediate-early nuclear antigen (Laib Sampaio et al., 2018, Biotechniques) in IF. In immunoblotting it detects two antigens with immediate-early kinetics absent from non-infected cells (see Ext. Data Fig 7b).

Eukaryotic cell lines

Policy information about cell lines and Sex and Gender in Research

Cell line source(s)	Primary lung fibroblast, Fi301, Institute of Virology, Susanna Prösch
Authentication	The cell line was not further authenticated.
Mycoplasma contamination	The cell line tested negative for Mycoplasma contamination

Commonly misidentified lines (See ICLAC register)

No commonly misidentifed cell line was used

Flow Cytometry

Plots

Confirm that:

☒ The axis labels state the marker and fluorochrome used (e.g. CD4-FITC).

☒ The axis scales are clearly visible. Include numbers along axes only for bottom left plot of group (a 'group' is an analysis of identical markers).

☒ All plots are contour plots with outliers or pseudocolor plots.

☒ A numerical value for number of cells or percentage (with statistics) is provided.

Methodology

Sample preparation

First, the cell culture supernatant containing floating cells was pipetted into a polypropylene centrifuge tube. The remaining adherent cells were washed with PBS and then detached from the plastic surface of the culture dish by incubation in Trypsin-EDTA. The resulting cell suspension was combined with the culture supernatant in the centrifuge tubes. Fetal calf serum was added at 10% final concentration to inactivate Trypsin. Cells were collected by centrifugation in a swing-out rotor at 300 g. After removal of the supernatant, the cell pellet was resuspended in 1 ml PBS. 3.5 ml of absolute ice-cold ethanol were added dropwise while vortexing the cell suspension at medium strength. Subsequently, samples were incubated for at least 16 h on ice. Next, cells were pelleted by centrifugation at 400 g and washed once with PBS. Then, the cells were incubated for 12-16 h on ice with an Alexa Fluor 488-conjugated antibody against the shared N-terminal domain of HCMV proteins IE1 and IE2 (Mab810X, Merck-Millipore, diluted in PBS/1%BSA). After washing once with PBS/1%BSA, cells were resuspended in PBS/ 0.1 mg/ml RNAse A/25 µg/ml propidium iodide (PI). After 15 min incubation at 25°C, samples were ready for flow cytometry.

Instrument

FACSCanto II flow cytometer (BD Biosciences)

Software

FACSDiva (BD Biosciences)

Cell population abundance

FACS sorting was done with TB40-BAC4 infected fibroblasts according to the IE1/2 expression. IE1/2 positive cell population abundance was between 4 % and 97 % of all living cells in the respective cell cycle compartment.

Gating strategy

A contour plot was created displaying on a linear scale the forward light scatter (FSC) and sideward light scatter (SSC) of measured particles. An FSC threshold was set for exclusion of cell debris. A region P1 was set that excludes larger cell debris and cell aggregates from further analysis. Based on the area (A) and width (W) of the propidium iodide fluorescence signal (PerCP channel), a P2 region was defined that excludes cell doublets and cells with a >2n DNA content from further analysis. Finally, based on cellular DNA content and on the Alexa Fluor 488 fluorescence (FITC channel) from IE1/IE2 immunostaining, four subpopulations of P2 were defined: P3 consists of IE-positive G1 cells; P4 of IE-positive G2/M cells, P5 of IE-negative G1 cells, P6 of IE-negative G2/M cells.

☒ Tick this box to confirm that a figure exemplifying the gating strategy is provided in the Supplementary Information.
