# Supplementary figures and images for: Spatially resolved protein map of intact human cytomegalovirus virions
Source: Nat Microbiol. 2023 Aug 7;8(9):1732–47. doi: 10.1038/s41564-023-01433-8 (PMC10465357; doi:10.1038/s41564-023-01433-8)

Fig. 4d

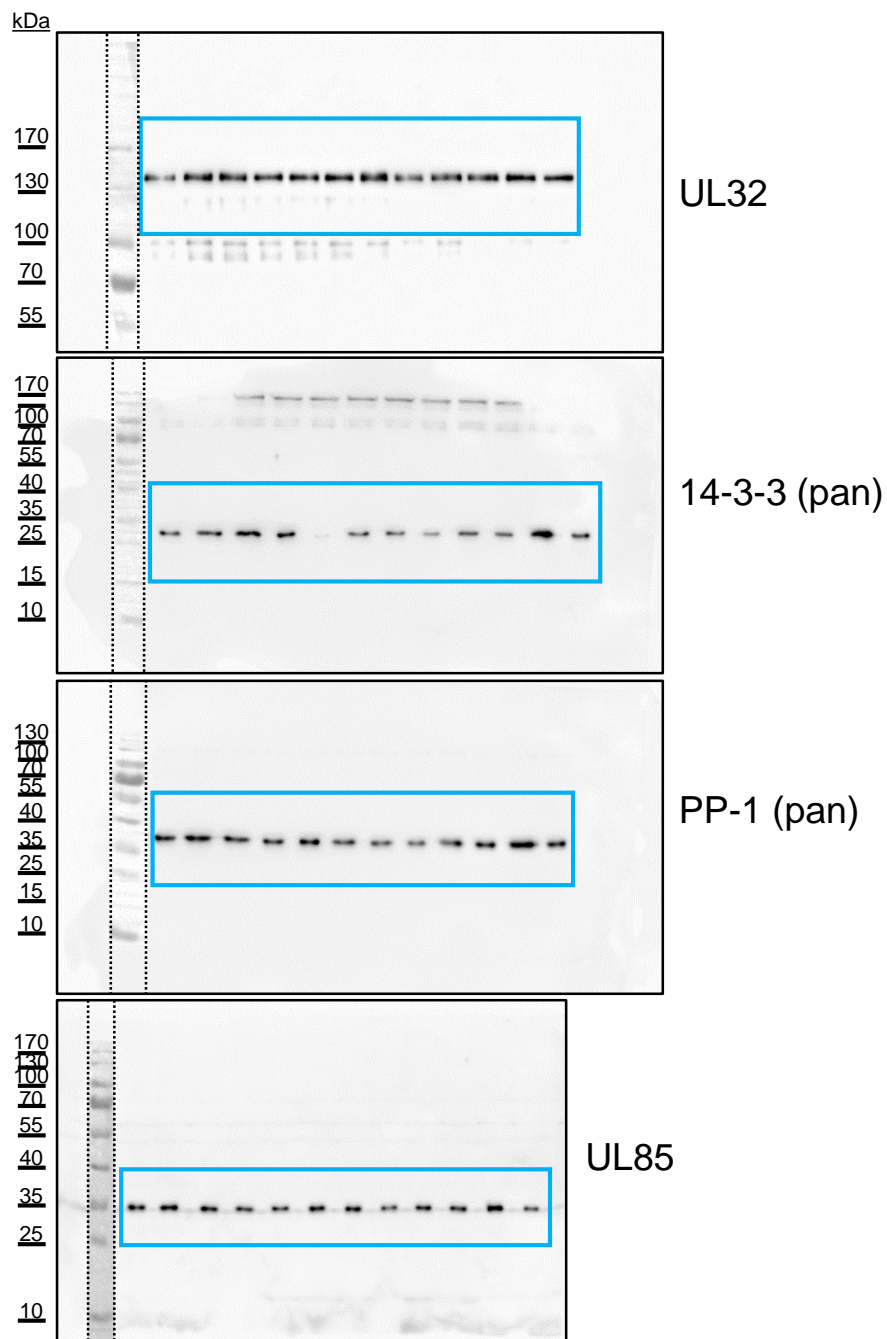

Fig. 4e

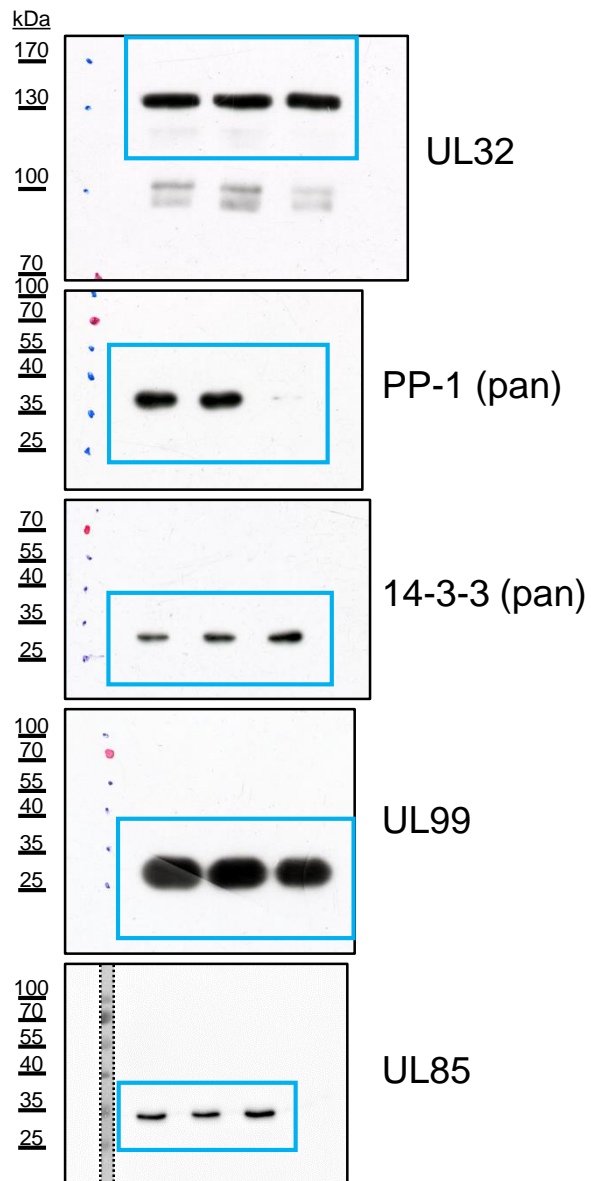

Ext. Data Fig. 5d

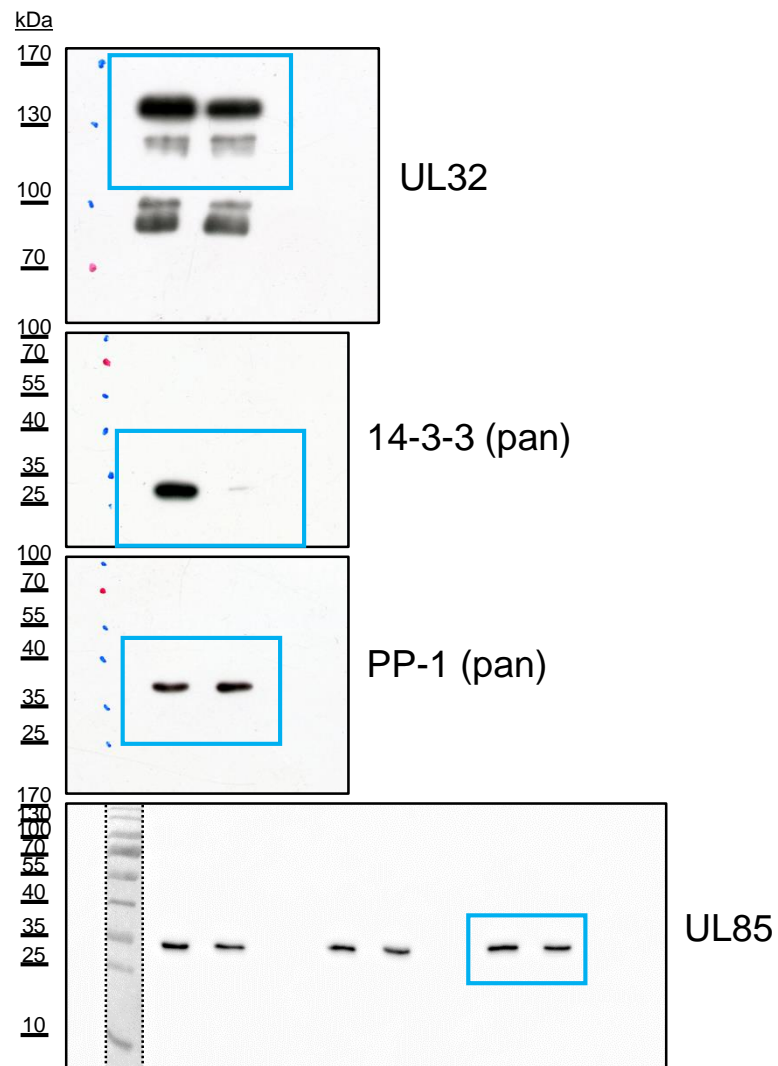

Ext. Data Fig. 5f

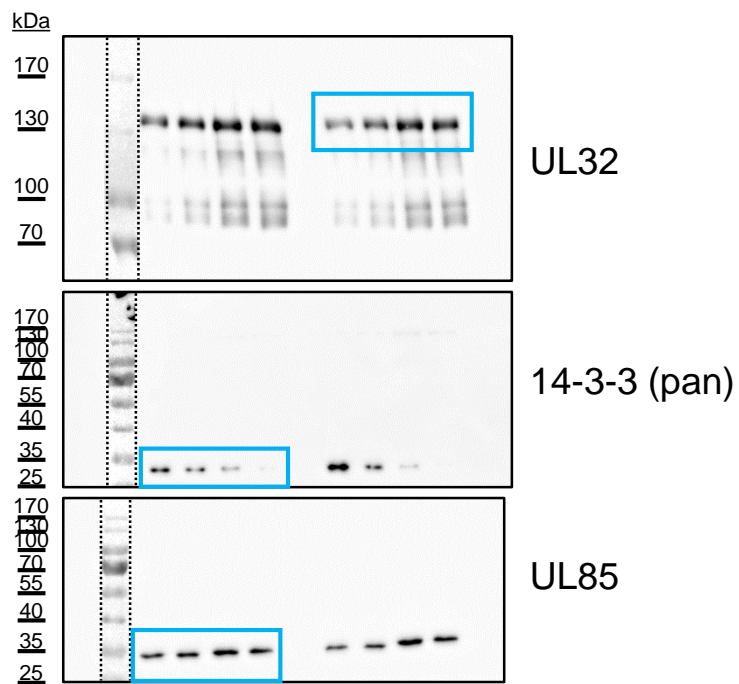

Ext. Data Fig. 7b

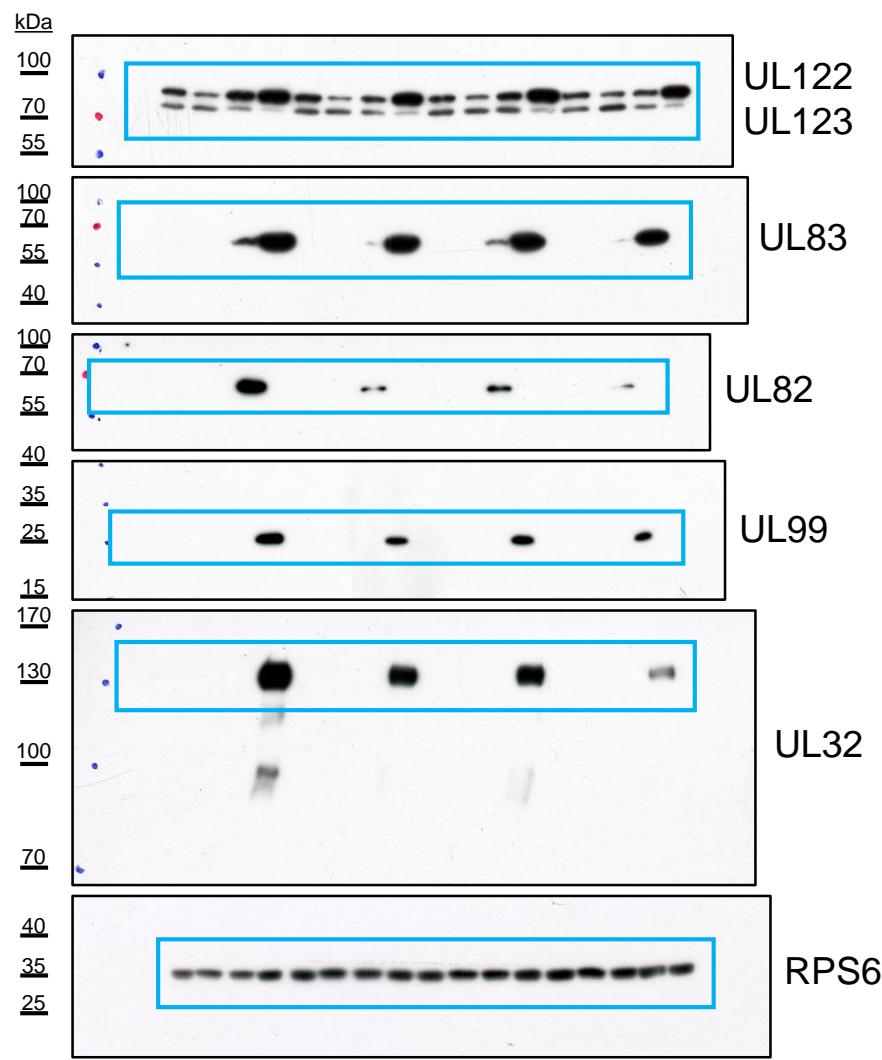

Ext. Data Fig. 7d

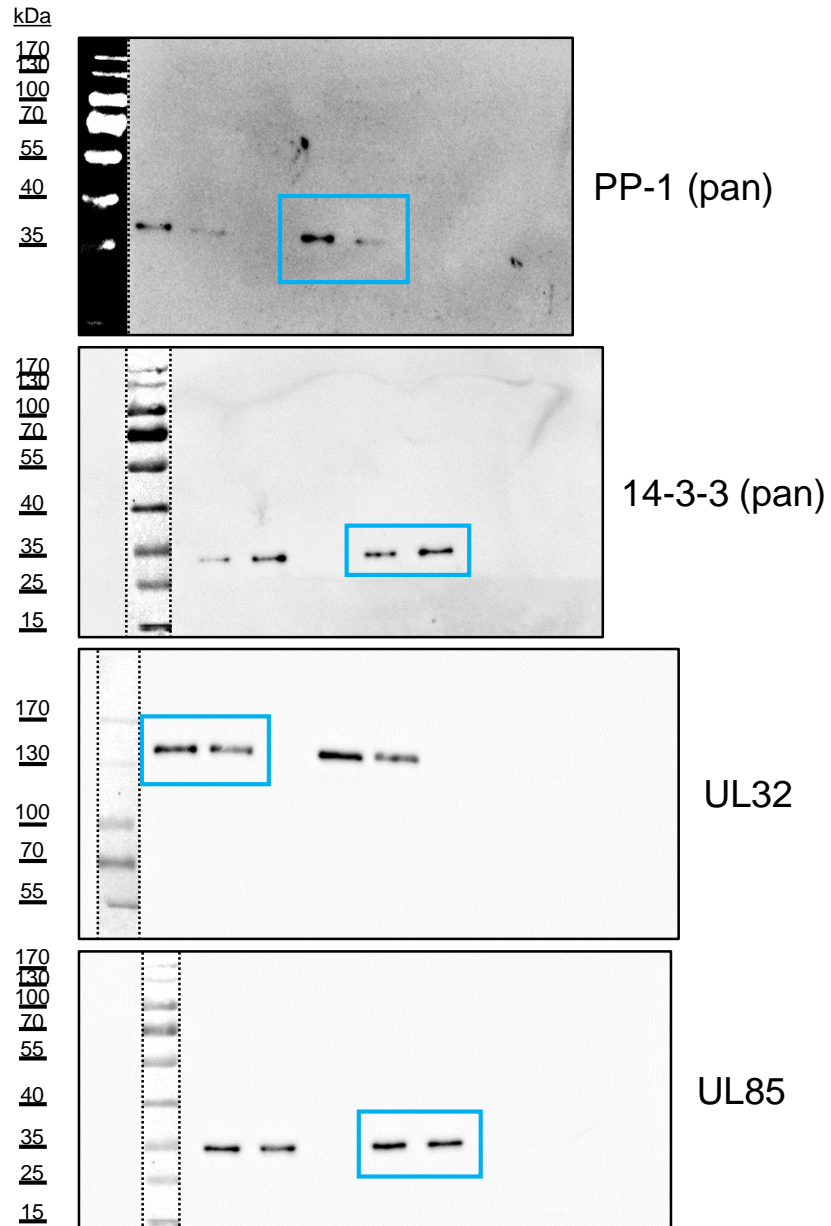

Ext. Data Fig. 9b

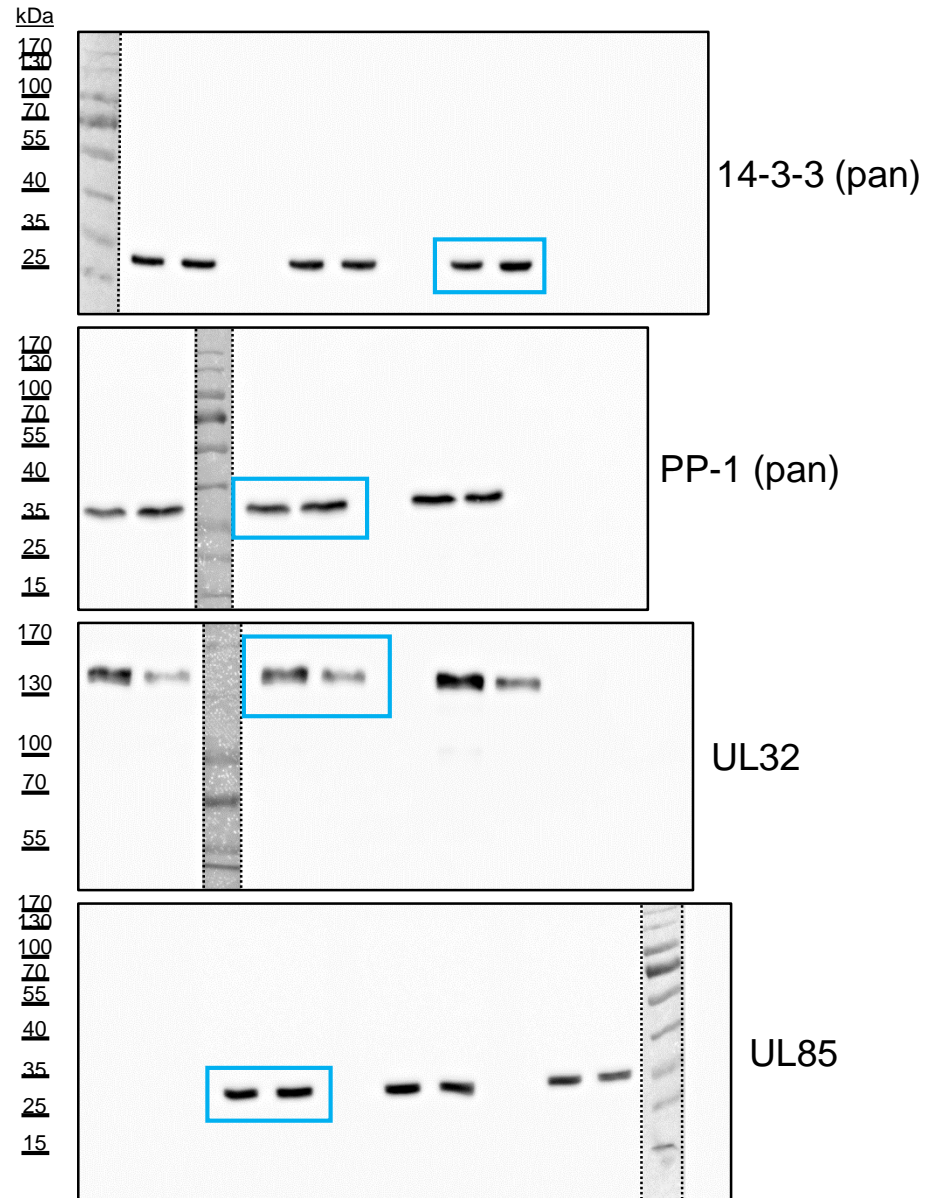

Ext. Data Fig. 9e

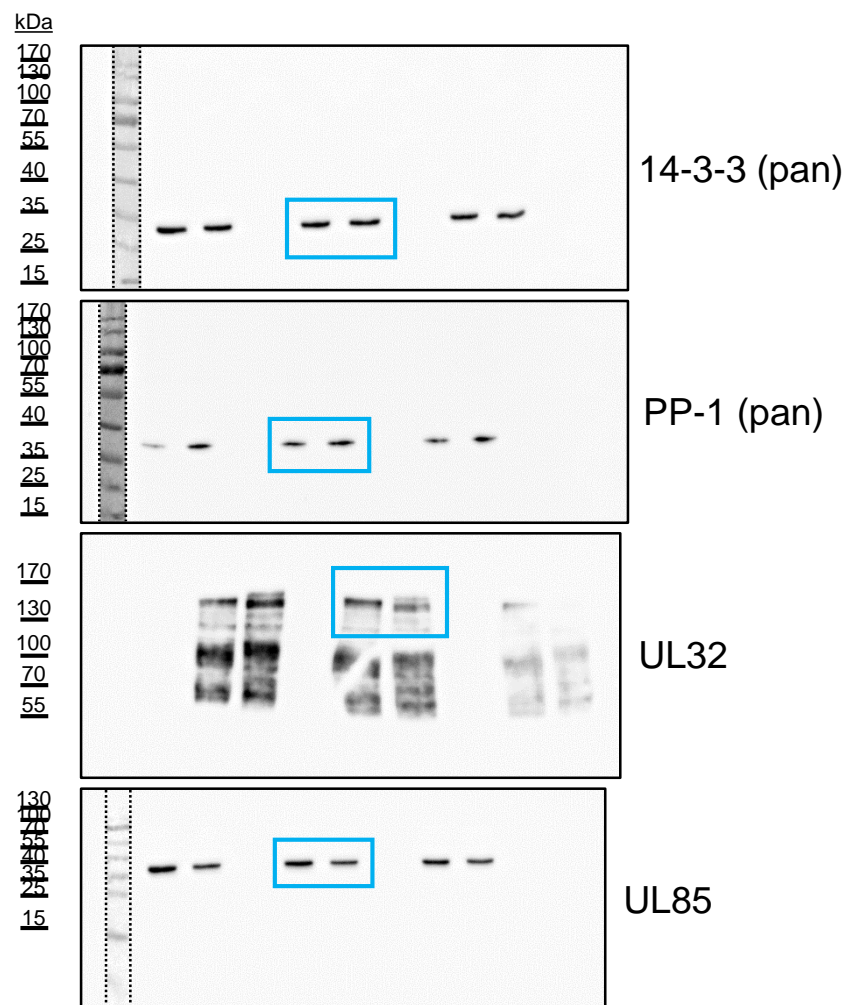

Supplement: Source Data Fig. 4, Source Data Extended Data Figs. 5, 7 and 9 — Statistical source data and unprocessed western blots. [file 41564_2023_1433_MOESM8_ESM.pdf]
