# Supplementary material for: Spatially resolved protein map of intact human cytomegalovirus virions
Source: Nat Microbiol. 2023 Aug 7;8(9):1732–47. doi: 10.1038/s41564-023-01433-8 (PMC10465357; doi:10.1038/s41564-023-01433-8)
Supplement: Supplementary file 3 — Peer Review File [file 41564_2023_1433_MOESM3_ESM.doc]

Peer Review Information

**Journal: Nature Microbiology**

**Manuscript Title:** Spatially resolved protein map of intact human cytomegalovirus particles

**Corresponding author name(s): Dr Fan Liu**

**Decision Letter, initial version:**

| **Message:** | 25th July 2022  *Please ensure you delete the link to your author homepage in this e-mail if you wish to forward it to your co-authors.  Dear Dr Liu,  Thank you for your patience while your manuscript "Spatial, Quantitative and Functional Deconstruction of Virus and Host Protein Interactions Inside Intact Cytomegalovirus Particles" was under peer-review at Nature Microbiology. As you have surmised we were waiting for a final reviewer to deliver but the process has been lengthy so we have processed a decision without the last report.  It has now been seen by 3 referees, whose expertise and comments you will find at the end of this email. Although they find your work of some potential interest, they have raised a number of concerns that will need to be addressed before we can consider publication of the work in Nature Microbiology.  One issue is novelty. This was a concern for the editors before peer review. In light of the novelty issues, please do let me know if you can do as reviewer 1 suggests and 'capitalise on the new data is as follows: with all of the information now gained, is it possible to develop an overall structural model of the HCMV virion (at least from a viral protein point of view)? The authors have high probability information as to protein content, stoichiometry, and, critically given their cross-linking studies, the likely structure of protein-protein interactions. This is in particular given the published high-definition structures of certain viral proteins such as gB, and the AlphaFold models of all HCMV proteins published by the Bosse lab. As they have established distance constraints in their examination of gB, and ~2200 cross-linked peptides, this (plus known information on capsid structure etc) could be leveraged to wider very interesting findings.'  Should further experimental data allow you to address these criticisms, we would be happy to look at a revised manuscript.  We are committed to providing a fair and constructive peer-review process. Please do not hesitate to contact us if there are specific requests from the reviewers that you believe are technically impossible or unlikely to yield a meaningful outcome.  We strongly support public availability of data. Please place the data used in your paper into a public data repository, if one exists, or alternatively, present the data as Source Data or Supplementary Information. If data can only be shared on request, please explain why in your Data Availability Statement, and also in the correspondence with your editor. For some data types, deposition in a public repository is mandatory - more information on our data deposition policies and available repositories can be found at https://www.nature.com/nature-research/editorial-policies/reporting-standards#availability-of-data.  Please include a data availability statement as a separate section after Methods but before references, under the heading "Data Availability”. This section should inform readers about the availability of the data used to support the conclusions of your study. This information includes accession codes to public repositories (data banks for protein, DNA or RNA sequences, microarray, proteomics data etc…), references to source data published alongside the paper, unique identifiers such as URLs to data repository entries, or data set DOIs, and any other statement about data availability. At a minimum, you should include the following statement: “The data that support the findings of this study are available from the corresponding author upon request”, mentioning any restrictions on availability. If DOIs are provided, we also strongly encourage including these in the Reference list (authors, title, publisher (repository name), identifier, year). For more guidance on how to write this section please see: http://www.nature.com/authors/policies/data/data-availability-statements-data-citations.pdf   If revising your manuscript:  * Include a “Response to referees” document detailing, point-by-point, how you addressed each referee comment. If no action was taken to address a point, you must provide a compelling argument. This response will be sent back to the referees along with the revised manuscript.  * If you have not done so already we suggest that you begin to revise your manuscript so that it conforms to our Article format instructions at http://www.nature.com/nmicrobiol/info/final-submission. Refer also to any guidelines provided in this letter.  * Include a revised version of any required reporting checklist. It will be available to referees (and, potentially, statisticians) to aid in their evaluation if the manuscript goes back for peer review. A revised checklist is essential for re-review of the paper.   When submitting the revised version of your manuscript, please pay close attention to our href="https://www.nature.com/nature-portfolio/editorial-policies/image-integrity">Digital Image Integrity Guidelines.</a> and to the following points below:  -- that unprocessed scans are clearly labelled and match the gels and western blots presented in figures. -- that control panels for gels and western blots are appropriately described as loading on sample processing controls -- all images in the paper are checked for duplication of panels and for splicing of gel lanes.  Finally, please ensure that you retain unprocessed data and metadata files after publication, ideally archiving data in perpetuity, as these may be requested during the peer review and production process or after publication if any issues arise.   [redacted]  <strong>Note:</strong> This url links to your confidential homepage and associated information about manuscripts you may have submitted or be reviewing for us. If you wish to forward this e-mail to co-authors, please delete this link to your homepage first.  Nature Microbiology is committed to improving transparency in authorship. As part of our efforts in this direction, we are now requesting that all authors identified as ‘corresponding author’ on published papers create and link their Open Researcher and Contributor Identifier (ORCID) with their account on the Manuscript Tracking System (MTS), prior to acceptance. This applies to primary research papers only. ORCID helps the scientific community achieve unambiguous attribution of all scholarly contributions. You can create and link your ORCID from the home page of the MTS by clicking on ‘Modify my Springer Nature account’. For more information please visit please visit <a href="http://www.springernature.com/orcid">www.springernature.com/orcid</a>.  If you wish to submit a suitably revised manuscript we would hope to receive it within 6 months. If you cannot send it within this time, please let us know. We will be happy to consider your revision, even if a similar study has been accepted for publication at Nature Microbiology or published elsewhere (up to a maximum of 6 months).  In the meantime we hope that you find our referees' comments helpful.  [redacted]   ***************************************************** Reviewer Expertise:  Referee #1: HCMV, antiviral proteins, proteomics Referee #2: 3d structural studies, cryoEM bioinformatics, tumor herpesviruses Referee #3: mass spectrometry approaches to characterize pathogen-host interactions  Reviewer Comments:  Reviewer #1 (Remarks to the Author):  In this study Bogdanow et al describe a spatial interactome within herpesvirus virions. They purified hCMV virions from human embryonic lung fibroblasts and utilised cross-linking MS and quantitative proteomics to determine the presence of four spatially distinct layers bridged by viral tegument protein Ul32. Ul32 was found act as a scaffold and specifically to recruit host proteins PP1 and 14-3-3 in a manner than was key for virus maturation. PP1 mediated phosphorylation was found to be key for recruitment of 14-3-3 proteins which in turn impacted viral replication. Although cross-linking MS has been applied in a number of contexts now, this represents an elegant application to the study of the HCMV virion. Overall the manuscript is presented and written very well, and the conclusions are backed up by the data provided. There are a number of changes that will improve the impact and clarity of both the text and the figures. The decision whether to accept to Nature Microbiology largely rests on the novelty of the data presented. The spatial aspect of the authors’ studies is clearly new, however other aspects of the data are known (or at least studied). In particular, the proteomics of the virion have previously been published as cited by the authors. The HCMV interactome has also been published. Furthermore, Stecher et al 2021 investigated the role of PP1 in the tegument, and identified a probable interaction with UL32. Overall, therefore, this manuscript although clearly of high quality might rest better with Nature Communications. However, one suggestion to increase the novelty and capitalise on the new data is as follows: with all of the information now gained, is it possible to develop an overall structural model of the HCMV virion (at least from a viral protein point of view)? The authors have high probability information as to protein content, stoichiometry, and, critically given their cross-linking studies, the likely structure of protein-protein interactions. This is in particular given the published high-definition structures of certain viral proteins such as gB, and the AlphaFold models of all HCMV proteins published by the Bosse lab. As they have established distance constraints in their examination of gB, and ~2200 cross-linked peptides, this (plus known information on capsid structure etc) could be leveraged to wider very interesting findings.  Main points 1. It would be helpful in the introduction to discuss in more depth what is already known, in order to highlight to the reader which of the findings are novel. The authors already cite proteomic studies by Varnum et al and Coute et al – these should specifically be discussed. A description of what is already known about localisation of tegument proteins would help. Similarly, in the results section, it would be very helpful to generate a supplementary table that has (a) details of the non-redundant 262 protein-protein interactions (in addition to Table S2, which shows the protein cross-links that constitute this data). (b) An explanation of the filtering applied; (c) details of known and novel interactions (e.g. in comparison to the published HCMV interactome – should also be discussed); (d) columns showing the predicted location in the layers of the virion for both interacting proteins; (e) details of existing data about location within the virion of these proteins. Finally, in the discussion, more could be added to place the results within the context of what is currently known in the field?  2. One conclusion of the paper is that proper incorporation of 14-3-3 proteins is crucial for viral replication. As perturbation of PP1 binding sites increases the amount of 14-3-3 incorporation and these viruses are slightly delayed in replication (Fig5d), is it possible to determine how 14-3-3 affects viral replication, for example by knockdown? Or possibly discuss why this cannot be performed.  3. There were a number of viruses created for this study. Only some had growth curves performed. Furthermore, there should be validation for the UL32 mutants that UL32 is expressed to a similar level both in virions and in cell lysates (e.g. using proteomics). a. Fig 4d Why were these particular motifs chosen to make the mutant viruses. I would like to see at least 3 or 5 mutated alone if the 3/4/5 result is to be believed. Also, this would benefit from quantitation as 14-3-3 in some samples appears to be increased for example, 1/2/3. Ext Fig 4d This would also benefit of quantitation b. Fig 4e Same comment on the virions, want to see quantitation and also comparison to major capsid protein. c. Line 320 Need to see blots to confirm no PP1 incorporation into the virion in this virus. d. Ext Fig 8a and B. Could comparison of the viruses be provided? Does gene expression occur as normal? Are both Ul32 expressed to the same level? e. Ext Fig 8c What effect does calyculin A have on infection? Are UL32 levels the same in both conditions, any other effects on replication?  4. Fig 3d (Lines 241-243) In this situation how is it possible to distinguish between the protein milleu of infected cells and the partially or fully constructed virions within the cells. Would this skew the relative protein numbers as there will be some virions within the cell lysate?  Other points  1. Protein protein interaction (PPI) and protein phosphatase 1 (PP1) are very similar acronyms. This lead to a bit of confusion when reading the text I suggest changing PPI to PP-I?  2. Ext Fig 1D – Why are there so many host proteins not linked to virus proteins excluded? Are these host-host interactions from proteins non-specifically incorporated in the tegument? Could the authors comment on the rationale? 3. Ext Fig 2a Why do the authors think that they identified so many fewer proteins than the previous studies. Does this adds up with all of the proteins excluded in Ext Fig 1D?  4. In the introduction (Line 54) authors mention that 32 viral proteins are mapped to hCMV virions during the study, however, in Fig2a only 31 viral proteins are listed on the heat map. It would add clarity to discuss the number of hCMV proteins already known to be in the virion, as there are many more than 31/2 total viral proteins  5. I would have liked to see a comment about the localisation of the binding sites identified on UL32 and what is already known about the structure of this protein. Particularly in regards to the date presented in Fig 3d.  6. Fig 5d. Not clear why is this one-step growth but Fig 5a is a multi-step?  Minor points Lines 37-39. Not clear from this sentence what facilitates the onset of viral gene expression. Line 72 Fig1A doesn’t strictly show crosslinking of lysines. Suggest changing text. Line 79. Seems to me that the second most abundant cross-link is with host proteins (Fig1C). Lines 81-82 Text mentions that no cross-links found between intravirion and virion surface protein domains. Suggest adding this to the legend of Fig1D for clarity. Fig1E Needs further clarification in text. Are the links mapped on the structures the ones found in the study? Do these map to the histograms shown in Ext Fig 1a and b? Fig1F found it hard to discern the difference between the magenta and the orange colours used in the figure. Fig 2b Scale is different for each of the sub-clusters. Line 151 Add reference for known role/localisation of Ul83 in the outer tegument. Line 188 Are these the only lysines on Ul86? Fig3A Ul32 rather than pp150 would be a clearer label. SuppTable 3 and 4 Some of the gene names have converted to dates Line 229 What about the protein copies not in the virion map (Fig3c). What are these? Lines 250-252 Why not mark these proteins on Fig3e Fig4/ExtFig4 What are PLF cells (never mentioned in the methods), are these the same as human embryonic lung fibroblasts. Fig4b Orange extremely hard to see here. Line 267 Clarification needed for how motifs were hypothesised. It is not clear from Fig4c. Line 278 Which residues were converted to alanine? Line 286 Significantly? P values not displayed? Line 307 20-fold fewer progeny at what time point? Need to clarify if this is total or extracellular virus used for the growth curves. Is recruitment of PP1 necessary for infectious viral progeny or for early steps post infection in the recipient cell? Fig5c What is PRH1. Labelled on chart but not discussed Line 384 Microtubuli? Is this correct. Line 711 Experimental design as in extended data as in fig 5c a typo? Potentially meant 5b?   Reviewer #2 (Remarks to the Author):  In this study, Bogdanow et al. employed cross-linking mass spectrometry (XL-MS) to build an extensive virion-wide proximity map of both viral and host proteins in intact extracellular virions of human cytomegalovirus (HCMV). HCMV is a leading viral cause of birth abnormalities and a significant public health concern due to opportunistic infection in AIDS and immune-suppressed populations. Consistent with, and complementary to, a series of recent cryoEM publications of HCMV virion structures showing interactions among virion and host components, the XL-MS data enable perhaps the most substantial depiction of host and virus proteins and their protein-protein interactions (PPIs) within and across different virion layers (envelope, tegument and capsid) of HCMV. In particular, the authors show that tegument protein pUL32 recruits PP1 phosphatase and 14-3-3 proteins and its role in early and late steps during virion assembly. The data established that PP1 antagonizes 14-3-3 binding to pUL32 and is required for efficient start of viral gene expression and production of viral progeny. Compared to previous MS publications, the current study represents a systematic characterization of the spatial coordination and interactions of virus and host proteins within virions. The paper is well written and illustrations nicely prepared. Overall, this paper provides global structural insights into the native configuration of virus and host protein interactions inside herpesvirus particles and has the potential to become a classic citation in the literature. I only have some minor issues that should be addressed.  Minor issues: 1. Throughout the paper, gene names (such as UL32) and protein names are used indiscriminately. This is likely to cause confusion. All protein names should have a prefix of p, such as pUL32. Alternatively, well established common names (pp150 for pUL32 and MCP for UL86, for example) should be (and preferentially) used. 2. Recent cryoEM has shown host RNA interaction with pp150 (Liu et al. Nat Comm). Could such interactions be detected by XL-MS? This should be commented at least in the Discussion.     Reviewer #3 (Remarks to the Author):  This study by Bogdanow et al. describes the analysis of herpesvirions by cross-linking mass spectrometry, a state-of-the-art technology that provides unique insight into spatial organization of complex proteomes. The authors identified over 9,000 crosslinks from which they could extract a high content network of protein-protein interactions (PPIs) in virions. By elegant calculation of PPI specificity values and hierarchical clustering, viral proteins were allocated to four different spatially resolved virion layers. Next to viral proteins, the authors report the layer-specific recruitment of 82 host proteins and they could distinguish specific from non-specifically recruited host proteins. Finally, the incorporation of host PP1 is shown to be important for viral replication by stimulating (early) viral gene expression and by antagonizing (late) incorporation of 14-3-3 proteins.  The study is well performed and the data is of high quality. The work is pioneering virion architecture mapping by XL-MS and therefore of high impact.  I do have a few points that need to be addressed though:  1. Do I understand it correctly that virions were first concentrated and cross-linked, and only purified afterwards? How did you control potential contamination from extracellular proteins, vesicles or debris that might become cross-linked to the particles? Did you check for instance the quality of the preparations by EM or any other method? At least potential limitations of the approach should be discussed.  2. Validation of the 4-layered virion architecture by an orthogonal approach or additional experiments would significantly strengthen the conclusions. I understand this might be difficult, but as a pioneering study in this area this might be worthwhile considering. The authors could for instance analyze mutant virions that are deficient in any of the main layer scaffolds (such as UL86, UL83 or UL100) and show that the architecture is disrupted. Again, if such experiments are not feasible, at least possible future validation and/or follow-up studies should be discussed.  3. The authors conclude that HCMV recruits PP1 to limit antiviral or perturbing effects of 14-3-3 proteins (discussion, line 374), but their findings should also be discussed in light of previous pro- or antiviral functions assigned to PP1 and 14-3-3 proteins.  4. Some concepts and analytical steps should be better explained in the main text: - line 96: please explain the rationale behind the further filtering criteria. - line 187: better explain how host proteins were assigned to the different virion layers. Also, provide a more extensive legend for Fig 3a. Where can I see the viral marker proteins in this figure? - line 325: please briefly explain that the AP-MS experiments were performed on cells infected with the different virus variants (and not for instance on cells transfected with UL-32 variants).  5. Please provide more details on the XL proteomics sample preparation (page 24-25), ideally by adding a step-by-step protocol as a Supplementary Methods file. At least following information should be added: - the amount of virions and cells the analyses were started from, and the amount of extracted/digested proteins - clearly indicate the number of experiments that were performed and the number of replicates/experiments - provide origin of trypsin and LysC enzymes and a brief description of the C8 and C18 purifications - provide full details on the strong cation exchange fractionation for cross-linked peptides, (e.g. what is the mobile phase?) or refer to previous published work  6. Similarly, provide more details on the AP-MS experiments, I am missing for instance information on the starting amounts of cells and , number of replicates, etc. Line 490: I assume proteins were eluted by adding 200 microliter of 8M guanidine hydrochloride?  7. A clear overview of all viral mutants and their sequences that were used in the study should be provided. For instance, I could not find the exact positions of the phosphosite mutants in Fig. 4. Also, what are the critical amino acid residues that were mutated in the SILK/RFxFmut variant? How were these mutant viruses created?  8. The authors submitted their raw data files to the PRIDE database, but I am missing a clear overview of which file(s) belong to which experiment(s). Please provide such overview, for instance as a Supplementary Table, so readers can re-use the files in a meaningful way.      ***************************************************** |
| --- | --- |

**Author Rebuttal to Initial comments**

***Point-by-point response***

We thank all three reviewers for reading our manuscript and for their constructive and valuable comments! We were particularly pleased to read that our “pioneering work” is of “high impact” (reviewer #3), “clearly of high quality” and “presented and written very well” (reviewer #1). We would also hope it to become a “classic citation in the literature” (reviewer #2).

We carefully addressed all points by the reviewers and performed a number of additional experiments and analyses that substantially strengthened the manuscript, corroborated our conclusions and significantly extended our findings. The major point raised by reviewer #1 was to integrate our data into a virion model. While this task took us longer than we had hoped, we are now satisfied with the result and think that our study is now ready for publication in Nature Microbiology.

(i) Most importantly, we provide a model of a viral particle that is based on integrative coarse-grained modeling using copy numbers provided by proteomic data and interaction restriction strength provided by cross-linking MS. The model takes into account (a) structural predictions, (b) cross-linking data, (c) copy number estimates and (d) realistic membrane kinetics. This is the first virion model of a complex DNA virus and allows for a refined view of the spatial organization of proteins in the virion **(see new Figure 6 and new Extended Data Figure 10)**.

(ii) We included additional control experiments/ analyses as suggested by reviewer #1. See Extended Data Figs. 2f,g, 7d,e, 9b,e and Fig. 5d.

(iii) We included additional growth curve experiments for selected viral mutants (Extended Data Fig. 7a and Figure R7, below).

(iv) We created a set of mutants allowing for a better understanding of the sequence determinants for 14-3-3 binding to UL32/pp150 (Extended Data Figure 5f,g).

(v) We added electron microscopy data showing the purity of particles upon density gradients and cross-linking (Extended Data Fig. 1) and supplied the readers with a detailed step-by-step protocol for XL-MS of virions (Supplementary Information).

Reviewer #1 (Remarks to the Author):

In this study Bogdanow et al describe a spatial interactome within herpesvirus virions. They purified hCMV virions from human embryonic lung fibroblasts and utilised cross-linking MS and quantitative proteomics to determine the presence of four spatially distinct layers bridged by viral tegument protein Ul32. Ul32 was found act as a scaffold and specifically to recruit host proteins PP1 and 14-3-3 in a manner that was key for virus maturation. PP1 mediated phosphorylation was found to be key for recruitment of 14-3-3 proteins which in turn impacted viral replication. Although cross-linking MS has been applied in a number of contexts now, this represents an elegant application to the study of the HCMV virion. Overall the manuscript is presented and written very well, and the conclusions are backed up by the data provided. There are a number of changes that will improve the impact and clarity of both the text and the figures.

The decision whether to accept to Nature Microbiology largely rests on the novelty of the data presented. The spatial aspect of the authors’ studies is clearly new, however other aspects of the data are known (or at least studied). In particular, the proteomics of the virion have previously been published as cited by the authors. The HCMV interactome has also been published. Furthermore, Stecher et al 2021 investigated the role of PP1 in the tegument, and identified a probable interaction with UL32. Overall, therefore, this manuscript although clearly of high quality might rest better with Nature Communications.

We thank the reviewer for the encouragement and her/his thoughtful comments! We agree that our manuscript provides the first spatial deconstruction of virus and host interactions for any virus and are happy to read that it is of high quality. However, we do not agree with the reviewer’s assessment regarding the novelty of our findings.

(i) It is true that the protein content of viral particles has been measured previously, leading to long lists of >2,000 proteins associated with purified particles. However, in this study we addressed three key points of biological relevance that were missing from previous studies: 1) copy number estimates, 2) which proteins are specifically recruited to the particles and 3) the pairwise interactions between proteins in the particle. Importantly, integrating these three angles we do not only know that a protein is present but also how many copies and based on which interactions it is likely recruited. Additionally, the enrichment ratios differentiate between non-specifically incorporated and selectively recruited proteins.

(ii) While the HCMV interactome has been published using AP-MS (see Noble et al., 2018, eLife) and thermal proteome profiling (Hashimoto et al, 2020, Nat Commun) in infected cells, the employed methods cannot address interactions inside viral particles, which is so far unexplored. We feel that our dataset has to be viewed as complementary compared to these other approaches that are performed from infected cells and using different methods. Indeed, both approaches are biochemically different and can report on different sets of interactors (Extended Data Figs. 2f,g). Our dataset provides an extensive interactomic landscape and dissects the molecular and structural details with respect to the viral particles.

(iii) Concerning the study by Stecher et al., 2021, we would like to point out that these authors did not investigate “the role of PP1 in the tegument”. Instead, in their study using PP-1 inhibitor experiments they found that PP-1 is important for translational regulation during infection. We provide a recruitment mechanism for PP-1, a functional role during early and late steps, and its mechanistic explanation. We reference Stecher et al. (ref. #52) in our new version of the manuscript.

However, one suggestion to increase the novelty and capitalise on the new data is as follows: with all of the information now gained, is it possible to develop an overall structural model of the HCMV virion (at least from a viral protein point of view)? The authors have high probability information as to protein content, stoichiometry, and, critically given their cross-linking studies, the likely structure of protein-protein interactions. This is in particular given the published high-definition structures of certain viral proteins such as gB, and the AlphaFold models of all HCMV proteins published by the Bosse lab. As they have established distance constraints in their examination of gB, and ~2200 cross-linked peptides, this (plus known information on capsid structure etc) could be leveraged to wider very interesting findings.

The idea of using the information gained from this study to develop an overall structural model of the HCMV virion is certainly intriguing. To address this, we performed a coarse-grained modeling of all viral and host proteins, including the nucleocapsid, tegument and membrane glycoproteins. In this coarse-grained model, individual proteins are approximated by beads. The bead radii for tegument proteins were determined by the radius of gyration obtained from the AlphaFold2 (AF2) monomeric predictions. The bead radii for viral glycoproteins and capsid proteins were approximated from the cryoEM or AF2 models. Here, to make the visualization more realistic, several differently sized subdomain beads were considered to give a shape reminiscent of those models (see our new Extended Data Fig.10 in the manuscript). The number of beads was given by the copy numbers and pairwise bead interaction constraints were quantitatively approximated from cross-linking data. The lipid bilayer was also modeled by beads according to a membrane model with a diameter of 190 nm (Sadeghi and Noé, Nat Commun, 2020). The simulation further allows for Brownian molecular movement of the individual beads. Beads were randomly placed and the simulation was run until the system reached its free energy minimum. Our new Supplementary video 1 shows equilibrium sampling of the model.

The most challenging part was to model the long disordered region of pp150/UL32, which we found to have domain specific associations. In order to fit it into the coarse grained model, we chopped the 1049 amino acid long protein into 11 segments based on molecular dynamics simulation of the AF2 predicted monomer. Each segment length was approximated from its radius of gyration and considered in the model as three beads, giving a chain of connected beads. Each of the segments was allowed to have independent interactions with other beads according to the segment-specific cross-links.

We leverage this model to better present and visualize the spatial localizations, copy numbers and shapes of proteins inside of the HCMV particle (see below Figure R2a). This represents a major advancement to our initial analyses as we incorporated important physical constraints into a model. We report this integrated model in the new Figure 6 of the revised manuscript. Importantly, this model recapitulates the spatial organization of UL32 within the tegument, giving each of its segments likely localization within the tegument (Figure R2b). In consistence with our cross-linking data, a fraction of the more c-terminal segments of UL32 contact the intravirion domains of viral glycoproteins.

**Figure R2. Coarse-grained model of a HCMV virion. (a)** Cross-sectional view through the equilibrium state with transparent membrane. Bead species identity as indicated. UL32 c-terminal segments make occasional contacts with intra-virion domains of viral glycoproteins. **(b)** Probability distributions of UL32 segments in the tegument. These data were added to Fig. 6.

Unfortunately, it is not currently feasible to generate a fully atomistic AF2-based virion model due to technical limitations.

There are a number of issues, when applied to (herpes)viral proteins specifically. (i) The multiple sequence alignment (MSA) of herpesviral genes covers generally less sequences than that of eukaryotic or prokaryotic organisms, which limits the possibility of MSA-based prediction methods for those herpesviral proteins that are not well conserved. (ii) AlphaFold2 performs poorly in chains without many homotypic contacts, a characteristic of disordered regions that are enriched in the tegument.

Despite these limitations, AF2 can generate monomeric structure of every viral protein, including some high-quality ones. However, additional challenges arise when applied to this specific task: Simply taking the AF2-predicted monomers, as made available by Jens Bosse of Hannover Medical School, will not help us to make an atomistic model of the particle since these proteins need to be assembled into complexes. For this to work properly, AlphaFold2 requires prior knowledge of the interaction stoichiometry of a complex, which is not provided by this or any other study. Even if this were provided, AlphaFold2 is not able to predict the orientation of a protein/complex within the particle. When applied to host-virus interactions, AF2 is expected to be even worse than in the case of virus-virus interactions. This is due to its reliance on capturing the co-evolutionary changes in amino acids as provided in the MSA. These evolutionary constraints are much stronger within one organism (host-host) than between two organisms (host-virus).

One could of course ignore these caveats and predict at least dimers, using AF2 multimer. To test if this is feasible, we have collaborated with Jens Bosse to predict all models of pairwise protein interactions involving viral proteins. The results of these predictions indicate that the vast majority of docking models did not have reliable quality (see Figure R3), underscoring the difficulties in developing an overall structural model of the virion using the information gained from this study and AlphaFold2. While AlphaFold2 may provide valuable insights for some interactions when interpreted cautiously, it is unlikely to give reliable results at a comprehensive level, which would be required for a “structural model”.

**Figure R3: Evaluation of host-virus and virus-virus predicted AF2 dimers from virion XL-MS-data.** Multimers for cross-linked PPIs were predicted using the AF2 algorithm and evaluated for quality by calculating pDockQ (predicted DockQ) scores, according to Bryant, Pozzati and Elofsson, Nat Commun, 2022. This pDockQ integrates the average pLDDT at a contact site and the number of residue-contacts for a PPI. pDockQ values below 0.23 can be considered poor, above 0.23 and below 0.5 acceptable and above 0.5 good.

In addition, any modeling attempt would require a good model on the viral protein UL32 as it serves as a scaffold for the assembly of viral and host proteins. Unfortunately, the AF2 model of UL32, being a disordered protein, is very poor in the C-terminal region (AA303-AA1049) (see Figure R4). AF2 will thus not help us to build an overall atomistic model as the most important scaffold is not adequately predicted. We thus decided for the mesoscopic simulation with coarse-graining individual proteins.

**Figure R4. AlphaFold2 model of UL32.** **(a)** Color coding by domain, 1-302 highlighted blue, 303-1049 (green). **(b)** color coded by model confidence.

Main points

1. It would be helpful in the introduction to discuss in more depth what is already known, in order to highlight to the reader which of the findings are novel. The authors already cite proteomic studies by Varnum et al and Coute et al – these should specifically be discussed. A description of what is already known about localisation of tegument proteins would help.

We thank the reviewer for these suggestions. We also agree with the reviewer that adding more content in the introduction and discussion is probably helpful. However, due to the length restriction set by the journal, we are not able to extensively discuss all aspects that these studies provide and therefore can only focus on the most important aspects.

We cited the proteomic studies identifying proteins from purified HCMV previously already in the context of identified/cross-linked proteins in the results section:

*“79 of these host proteins have been observed in previous proteomic studies as associated with purified virions using standard proteomics* [*^15–18^*](https://paperpile.com/c/sf2lJz/dUz2+cv7s+C3gJ+dRpL) *(Extended Data Figs. 2a-b)”*

Now additionally in the Introduction:

*“In addition, previous studies only provided insight into the overall protein composition of herpesvirions* [*^14–18^*](https://paperpile.com/c/sf2lJz/s9goN+dRpL+cv7s+dUz2+C3gJ)*”*

And, in the Methods section:

*“Including several proteins as reference points is expected to give more reliable copy number estimates than previously used one-point calibration of the intensity of UL86 with its copy number* [*^16,18^*](https://paperpile.com/c/sf2lJz/C3gJ+cv7s)*“*

Furthermore, we added the following paragraph into the discussion, putting our findings in context to existing observations:

*“While many of the allocations are in congruence with previous observations*[*^16,29,32–34^*](https://paperpile.com/c/sf2lJz/gHIu+syq9+EwiV+X7vQ+cv7s)*, some were unexpected. For example, pp71/UL82 and UL35 clustered to the nucleocapsid-proximal tegument but have previously been associated with typical outer tegument properties, such as detergent solubility and nuclear trafficking*[*^3,30,31^*](https://paperpile.com/c/sf2lJz/aeEP+G7Tr+wfKt)*. In this context, it is interesting that the release of pp71/UL82 from the nucleocapsid-proximal tegument may have regulatory potential*[*^44^*](https://paperpile.com/c/sf2lJz/ZLbc)*.”*

Similarly, in the results section, it would be very helpful to generate a supplementary table that has (a) details of the non-redundant 262 protein-protein interactions (in addition to Table S2, which shows the protein cross-links that constitute this data). (b) An explanation of the filtering applied; (c) details of known and novel interactions (e.g. in comparison to the published HCMV interactome – should also be discussed); (d) columns showing the predicted location in the layers of the virion for both interacting proteins; (e) details of existing data about location within the virion of these proteins.

We made a new Supplementary Table 3 with the suggested columns, a and c-e. According to our filtering, all interactions contained in Supplementary Table 2 survived the filtering. We therefore did not include the criterion (b) in Supplementary Table 3.

The comparison to the dataset from the Weekes lab’s pioneering study of HCMV protein interactions in infected cells via AP-MS is a very good idea! We observed a relatively low overlap of interactors (~5 %), underscoring that these datasets should be viewed as complementary (see below Figure R5). In total, we could identify prior evidence for PPIs involving viral proteins for ~20 %, when taking other publications into account.

**Figure R5. Comparison of PPIs to published datasets. (a)** For comparison, we included annotated interactors from Nobre et al., 2018, eLife of viral baits that were also included in the XL-MS PPI-map. PPIs common to both datasets are depicted with their gene names. **(b)** Number of PPIs identified in this study with and without existing prior evidence in the literature. Referenced publications include: Y2H (To et al.,2011, PLoS One, Phillips et al., 2011, J Virol), AP-MS (Nobre et al., 2018, eLife; Du et al., 2013, PLoS One, Moorman et al., 2009, MCP), co-IPs (To et al.,2011, PLoS One), cryoEM (Wrapp et al., 2022, Sci Adv, Dai et al., 2013, PLoS Pathog; Yu et al., Science, 2017), and biochemical experiments (Mun Kwon et al., 2021, J Virol, Hamilton et al., 2018, Placenta).

We added these data to Extended Data Fig. 2. and refer to it in the Results:

*Of the PPIs involving viral proteins, 5 % were found in an AP-MS dataset from infected cells* [*^26^*](https://paperpile.com/c/sf2lJz/ybFA) *and an additional ~15 % had prior evidence in the literature (Supplementary Table 3, Extended Data Figs. 2 f,g).*

Finally, in the discussion, more could be added to place the results within the context of what is currently known in the field?

We substantially extended the discussion section, including the following paragraph:

*“We compared our XL-MS dataset from intact virions to a previously published large scale AP-MS dataset from infected cells, finding ~5 % of overlap in PPIs. When interpreting this number, it is important to keep limitations and strengths of both approaches in mind. First, this is comparing interactions from infected cells to interactions in virions, where the relative abundances of host and viral proteins are divergent. AP-MS interactors need to withstand cell lysis, washing and affinity purifications, possibly losing low-affinity interactions and potentially creating interactions between proteins that may not be close to each other in the intact cell.*

*XL-MS identifies contact sites from proteins in proximity in their intact environment. While this retains low-affinity interactors, it is possible that some interactions cannot be observed when the environment is disrupted. Our data also do not allow us to draw conclusions about the affinity of two interaction partners, especially in environments of macromolecular crowding. In addition, the large complexity of cross-linked peptides makes it challenging to identify cross-links from low abundant interaction partners. In our situation, this appears not to be particularly problematic, as our map included >91 % of the protein copies inside a particle.”*

And a paragraph about PP-1 and its role in viral infections (see also our response to reviewer #3, point 4):

*“PP-1 is a target that is exploited by various viral proteins, such as HSV-1 ICP34.5* [*^48^*](https://paperpile.com/c/sf2lJz/9bnZ)*, HSV-1 pUL21* [*^49^*](https://paperpile.com/c/sf2lJz/J49F)*, Measles/Nipah virus V protein* [*^50^*](https://paperpile.com/c/sf2lJz/vbiU) *and HIV Tat* [*^51^*](https://paperpile.com/c/sf2lJz/jmPz)*. This regulates phospho-sensitive processes such as translation* [*^48^*](https://paperpile.com/c/sf2lJz/9bnZ)*, transcription* [*^51^*](https://paperpile.com/c/sf2lJz/jmPz) *and RNA-sensing* [*^50^*](https://paperpile.com/c/sf2lJz/vbiU)*. Recruitment of PP-1 to UL32 is important for high-titer replication of HCMV (Figure 5a) , consistent with previous inhibitor experiments targeting the substrate recruitment site of PP-1* [*^52^*](https://paperpile.com/c/sf2lJz/zi6j)*. We show that this interaction modulates the phosphorylation status of UL32 and positively regulates the onset of viral gene expression (Figure 5b,d, Ext. Data Fig. 6d).”*

2. One conclusion of the paper is that proper incorporation of 14-3-3 proteins is crucial for viral replication. As perturbation of PP1 binding sites increases the amount of 14-3-3 incorporation and these viruses are slightly delayed in replication (Fig5d), is it possible to determine how 14-3-3 affects viral replication, for example by knockdown? Or possibly discuss why this cannot be performed.

The suggestion of the reviewer makes perfect sense! However, it is difficult to knock-down all seven 14-3-3 isoforms. Except for 14-3-3-σ, we found all of them interacting with pp150/UL32, suggesting functional redundancy. We therefore think that a single, double or even triple knockdown experiment is unlikely to give meaningful results. In contrast, overexpression of 14-3-3 proteins is one possibility to test their (perhaps antiviral) function (see for example Riedl et al., Cell Host Microbe, 2019). Given that 14-3-3 proteins are among the most abundant proteins in cells, very high overexpression levels would be required, as is achieved for example by transient transfection. However, cell lines permissive for HCMV are very difficult to transfect. We therefore created stably transduced fibroblasts expressing a HALO-tag fused 14-3-3 protein (YWHAZ). The observed overexpression level compared to the endogenous 14-3-3 levels was, unfortunately, not high enough to allow for experimental assessment of a potential overexpression phenotype (see Figure R6, below).

**Figure R6: Stable ectopic expression of an epitope tagged 14-3-3 variant.** To create an experimental system for analyzing the effects of 14-3-3 overexpression, we cloned the 14-3-3 isoform YWHAZ together with a Halo epitope tag into the lentiviral expression vector pCDH-CMV-MCS-EF1-Puro (System Biosciences). To avoid transcriptional repression by the UL122/IE2 gene product of HCMV, we mutated the cis repression signal (crs) within the CMV promoter. We generated lentiviruses on 293T cells and used them to infect HEL fibroblasts. After puromycin selection of stably transduced cells, we superinfected those cells by HCMV and measured 14-3-3 expression at the indicated time points by immunoblot analysis.

3. There were a number of viruses created for this study. Only some had growth curves performed. Furthermore, there should be validation for the UL32 mutants that UL32 is expressed to a similar level both in virions and in cell lysates (e.g. using proteomics).

We thank the reviewer for this comment. We would also like to point out that we purposefully did not perform growth curve analysis of viruses where we did not observe any effects on incorporation of virion proteins (e.g. virus mutants: 6, 6/7, 3, ,3/6, 1,1/2, 1/2/6,1/2/3, 1/2/3/6 Figure 4d). However, we agree that growth curve analyses of virus mutants that are used for functional experiments are probably helpful for the reviewer to evaluate our results.

Below are additional growth curves for 14-3-3 binding deficient virus (3/4/5mut), the single cyclin A-binding site mutant (RXLmut) and combined PP1-binding/cyclin A mutants (RXL/SILK/RVXFmut). In contrast to the SILK/RVXF motif mutation, mutation of the 14-3-3 binding sites or the RXLmotif alone did not have any effect on virus growth. The growth defect of the SILK/RVXFmut could be partially rescued by an RXL motif mutation. This effect is barely significant (p=0.042), we therefore did not include it in the original version of the manuscript. We explain the partial rescue by the ability of the RXL/SILK/RVXFmut virus to (i) start IE-gene expression more efficiently than the SILK/RVXFmut (see Fig. 5b) and (ii) by its lower affinity for CDK-dependent phosphorylation (Bogdanow et al., PNAS, 2013), which may affect binding of 14-3-3 at late stage. We do not include these data in the revised version of the manuscript, as it is already quite long.

**Figure R7: Growth kinetics of select mutant viruses.** MOI = 5 on primary lung fibroblasts. All viruses were grown in parallel. WT, SILK/RVXFmut and SILK/RVXF/3/4/5mut are also displayed in Figure 5e.

Additionally we included growth curves when only one of the PP1 interaction motifs were mutated (see also subpoint d below) into Extended Data Figure 7a.

For the UL32 levels, please see our controls related to subpoint d below.

a. Fig 4d Why were these particular motifs chosen to make the mutant viruses. I would like to see at least 3 or 5 mutated alone if the 3/4/5 result is to be believed. Also, this would benefit from quantitation as 14-3-3 in some samples appears to be increased for example, 1/2/3. Ext Fig 4d This would also benefit of quantitation.

We selected these sites for various reasons. First, there are simple technical reasons in the design of the mutants by, for example, a mutagenesis primer able to target several sites in one go. Second, we considered that 14-3-3 bind as dimers, thereby typically targeting several motifs on the same protein that may be adjacent. Third, we considered prediction tools, suggesting several sites as potential targets (Fig. 4c). Fourth, we closely investigated the surrounding neighborhood of potential sites, particularly in respect to identified phosphorylation sites (Fig. 4c).

Concerning the additional motifs, we now present more detailed analyses for prerequisites of 14-3-3 binding and mutated 4+5, 3+4, and 3 alone. We observed the strongest effect for 4+5 and site 3 contributes partially. We thank the reviewer for bringing this to our attention, which improved the MS (see below Figure R8).

**Figure R8. Contribution of individual sites in 3/4/5 motifs for incorporation of 14-3-3 into virions.** **(a)** Exemplary western blot of purified virions blotted for UL32, 14-3-3 and UL85. **(b)** quantitation of UL32 and 14-3-3 protein levels based on n=4 replicates normalized to UL85 and WT. p-values based on unpaired two-sided t-tests. We added these data into Extended Data Figure 5f and g.

Concerning the quantitation of western blot data. We do not think that it is necessary to perform quantitative western analysis when we validate our findings by another quantitative approach (quantitative (SILAC) proteomics, see Ext. Data Fig. 5e). Therefore we did not perform this analysis.

b. Fig 4e Same comment on the virions, want to see quantitation and also comparison to major capsid protein.

We have performed quantitative MS experiments (see Extended Data Fig. 6b) and therefore did not perform quantitative Western blotting. The idea to blot for an additional viral protein makes perfect sense as we also mutated UL32. Following the reviewers comment, we additionally blotted minor capsid protein (UL85, mCP) and included this in the Figure panel (Figure 4e). We do not include blots for MCP, as our antibody did not yield sufficiently clean bands.

c. Line 320 Need to see blots to confirm no PP1 incorporation into the virion in this virus.

We thank the referee for this comment! Indeed this is an important control. As we also did not do quantitative mass-spectrometry, we followed the reviewers comment and performed western blot analysis + quantitation (Figure R9). This shows that less PP-1 is incorporated upon SILK/RVXF mutation and that instead more 14-3-3 is incorporated, consistent with our conclusions (Figure 5f).

**Figure R9. PP-1 incorporation in the virions in RXLmut genetic background.** (a) Exemplary western blot analysis of select viral and host proteins from purified virions. (b) Quantitation of n=4 (14-3-3) or n=3 (PP-1, UL32) replicates. P-values are based on unpaired two-sided t-tests. We added this control to Extended Data Figure 7d,e.

d. Ext Fig 8a and B. Could comparison of the viruses be provided? Does gene expression occur as normal? Are both Ul32 expressed to the same level?

We thank the reviewer for this comment. As expected from the growth curve analyses in Fig. 5b (see also below Figure R10a), we indeed observed slower progression of viral gene expression kinetics with the mutant virus (Figure R10b). Note that we observed an intermediate phenotype with viruses where only one of the binding sites were mutated (SILKmut, RVXFmut), both in growth and kinetic of viral gene expression.

**Figure R10. Growth curves and viral protein level kinetics of single and double PP1-binding deficient mutant viruses.** These data were added to Extended Data Figure 7 a,b.

Accordingly, both UL32 are not expressed to the same level and instead less UL32 is present with the mutant. To corroborate this finding by a quantitative approach, we performed proteomic analysis of whole cell lysates, showing that almost all viral proteins are less abundant with the mutant **(see Figure R11, below)**.

**Figure R11. Protein levels from infected cell lysates at 5 dpi and MOI=5, comparing WT to RVXF/SILKmut infection.** The experiment was performed label-free in n=3 replicates, showing mean differences and a t-test p-value based on the replicates. Grey lines indicate a log2 fold-change of 1/-1 and p-value at 0.01.

In principle, it is possible that the UL32 differences in the lysates impact our analysis of UL32 interaction partners by AP-MS and quantitative proteomics. For example, when UL32 is more abundant in the precipitates with the wildtype, its interaction partners may also be more abundant, making it seem as if the interaction partners to UL32 are diverging between wildtype and mutant. Here, it is important to make sure that the levels of UL32 in the precipitates are equal. Indeed, after AP, we observed rather similar levels of UL32 (see Fig. 5c, UL32 highlighted), thus allowing for a comparison of the wildtype and mutant interactome. The AP balances the whole cell lysate differences, probably because the AP against pp150 (a highly abundant protein, rank #113 in the lysate) is performed under saturating conditions. In order to fully rule out that differences in abundances in the whole cell lysates interfere with the AP-MS level differences, we designed an orthogonal experiment with the Phosphatase inhibitor treatment (see below, subpoint e). Importantly, both approaches find 14-3-3 enriched with UL32 when binding or activity of PP-1 is perturbed.

e. Ext Fig 8c What effect does calyculin A have on infection? Are UL32 levels the same in both conditions, any other effects on replication?

We thank the reviewer for her/his question and would like to point out that it is not expected that this short treatment (20 min, directly before harvest) leads to pronounced differences in protein levels. The situation is of course different when Calyculin A is added pre-infection or shortly after inoculum, when it can block the accumulation of viral proteins (see Stecher et al., 2021, Future Microbiology, Figure 2G). This, however, we did not do. To validate our expectation we blotted UL32 levels after calyculin A treatment, indicating no important differences (Figure R12a).

The expected consequence of short-term phosphatase inhibitor treatment directly before harvest is not on overall UL32 protein levels, but instead on its phosphorylated proteoforms. To test this, we analyzed the mass-spec runs of our calyA/DMSO treated and UL32-GFP precipitated samples for identified peptides and phosphopeptides (Figure R12b). Our analysis shows approximately the same number and intensity of overall identified UL32 peptides. However, consistent with more UL32 being phosphorylated upon calyculin A treatment, we identified more and higher abundant phosphopeptides of UL32 under calyculin A. Thus, under calyculin A, pp150 is stronger phosphorylated, consistent with stronger binding of 14-3-3.

**Figure R12. Calyculin A treatment increases phosphorylation of UL32.** (a) Lysate levels of selected proteins. (b) intensities of pp150/UL32 derived peptides or phosphosites. These data were added to Extended Data Figure 8 or Figure 5d.

4. Fig 3d (Lines 241-243) In this situation how is it possible to distinguish between the protein milleu of infected cells and the partially or fully constructed virions within the cells. Would this skew the relative protein numbers as there will be some virions within the cell lysate?

This is a good point! The reviewer is right, there are many late viral proteins within the cell that may be part of partially or fully, but not yet released, viral particles. The reviewer is also right that we cannot distinguish “between the protein milieu of the infected cell and [...] virions within the cells”. In our case, both are merged as both are present inside the cell. It is very likely that this results in lower enrichment ratios of e.g. structural proteins, than in the hypothetical case when “partially or fully constructed virions" within the cells were removed.

Although our analysis likely provides lower enrichment ratios, we believe that these ratios give great indication on the specificity of protein incorporation. To illustrate this, we compare our data to Varnum et al., 2004, J Virol, and assess the enrichment ratios of proteins that were considered structural (capsid, glycoproteins, tegument) and non-structural (e.g. transcription, or replication-related) in the original publication. Although all of these proteins are routinely identified from purified particles, our experiment clearly separates these two classes of proteins (Figure R13). This result shows the quantitative ratios are very helpful in determining which proteins are specifically recruited to the particles, and that these transcription- and replication-related proteins are not enriched above their cellular levels in virions.

**Figure R13 HCMV proteins identified by Varnum et al., J Virol, 2004 mapped to enrichment ratios**. Annotations as published by Varnum et al., J Virol, 2004, except UL45, which is better considered tegument and not transcription/replication related, as it has lost RNR (ribonucleotide reductase) function.

Indeed, looking at the distribution of ratios comparing cellular to viral protein levels more broadly, we observed that most proteins, although many of them can be reliably identified in virions, are not specifically recruited (see below, Figure R14).

**Figure R14. Distribution of enrichment ratios for host and viral proteins.**

Other points

1. Protein protein interaction (PPI) and protein phosphatase 1 (PP1) are very similar acronyms. This lead to a bit of confusion when reading the text I suggest changing PPI to PP-I?

Thanks, we agree! A common acronym for PP1 is also “PP-1”. We replaced PP1 accordingly in text and figures.

2. Ext Fig 1D – Why are there so many host proteins not linked to virus proteins excluded? Are these host-host interactions from proteins non-specifically incorporated in the tegument? Could the authors comment on the rationale?

To begin, we would like to comment on why so many of the links come from these host proteins in the first place. This is because the number of links that we identify for proteins (i.e. host and virus) mainly depends on the abundances of the proteins (i.e., generally speaking the higher abundant a protein presents in the sample, the more cross-links one could identify from this protein). In our purified virion sample, the overall abundance of the viral proteins is much higher than that of host proteins, but the variety of the host proteins co-purifying with the virion is much higher than that of viral proteins. This results in the identification of many but sparse cross-links on host proteins.

There are three reasons of why many host proteins were excluded:

First, XL-MS identifications of intra-links (cross-links within the same protein sequence) are much more frequent than inter-links (cross-links connecting different protein sequences). In particular, lower abundant proteins will sometimes only yield intra-links, while the higher abundant ones will yield inter- and intra-links.

The relevant biological information we can draw from proteins that are only intra-linked is, unfortunately, limited, as we would only be able to assess the structure of the monomer (or homomer). Therefore, we excluded these from our map. From 1,292 host proteins for which we identified a cross-link, 745 exclusively were identified by intra-links, explaining the largest portion of the host proteins that were excluded. The level of information that we have about these is basically only that they are present in the sample.

Second, we observed some host proteins to form independent networks (potentially co-purified host proteins in our virion preparation) or are higher-degree interactors to a viral protein (that is, they are connected to a viral protein only via at least one other host protein). In these cases, we cannot be completely sure that such a higher-degree host protein is part of the particle, as cross-linking is only able to give information on binary interaction. We reasoned that it is more conservative to remove them from the XL-MS virion map and therefore did so, reducing the list further to 204 host proteins (only those that are directly interacting to viral proteins).

Third, we wanted to predict likely localizations for these proteins within the virion (see Figure 3a). To do so, we required a host protein to have at least two cross-links to viral proteins to include it in our XL-MS virion map. In addition, we filtered for cross-link identification reproducibility by requiring identification of the cross-link in both replicates for high abundant proteins (> 10 copies) or either of the replicates when it is lowly abundant (< 10 copies). This latter filtering takes into account that there are many lowly abundant proteins for which we cannot expect identification reproducibility, thus increasing their sensitivity. By these filtering steps, we ended up with 82 host proteins.

3. Ext Fig 2a Why do the authors think that they identified so many fewer proteins than the previous studies. Does this adds up with all of the proteins excluded in Ext Fig 1D?

Indeed, we “only” include 82 host proteins into the virion map. This is due to the three reasons as discussed in point #2. Below we comment on how important these “excluded” proteins are:

(i) The most important reason that we have many fewer proteins is that we only included proteins that we are sure to be incorporated in the virion rather than proteins present in the sample. As discussed in point 2, we only included host proteins that have direct cross-links with viral proteins, indicating they are either incorporated inside of the virion or tightly associated with glycoproteins. Previous proteomic experiments only provide information on what proteins present in the sample, but these proteins could well be co-purified host proteins during virion purification. We actually also performed proteomics in our study and indeed in our analysis of linear peptides the number of identified proteins from purified particles (2,254 protein groups, see Supplementary Table 4) is on par and even exceeds some of the numbers in the literature.

(ii) From our quantitative assessment of protein copy numbers, we show that the proteins included in our PPI-map (82 host proteins and 42 viral proteins) already cover 91% of protein copies within the particle (see Fig. 3C). This indicates that the many other proteins reported by previous studies are either extremely low abundant in the virion or simply co-purified proteins in the sample. For example, our MS-analysis of purified virions indicates that more than 85 % of proteins are incorporated with fewer than 1 copy on average (Supplementary Table 4). The lowest copy number we quantified is: ~ 1 * 10^-5^ per virion.

4. In the introduction (Line 54) authors mention that 32 viral proteins are mapped to hCMV virions during the study, however, in Fig2a only 31 viral proteins are listed on the heat map. It would add clarity to discuss the number of hCMV proteins already known to be in the virion, as there are many more than 31/2 total viral proteins

The reviewer is right, we mentioned in this line that we “build a virion-wide proximity map of 32 viral and 82 host proteins in intact extracellular virions of human cytomegalovirus”. This refers to the proximity-based map in Fig. 1f, which contains 32 viral proteins in the interconnected part of the network. We included only 31 proteins in the heatmap as we did not consider proteins with cross-link counts below a cut-off of 9 in order to have sufficient data points for the clustering.

The reviewer addresses an important point. What is the number of viral proteins present in the virion? The literature gives estimates between 51 and 73, with an intersection of 46 and union of 82. We find 104 with standard linear proteomics, of which 53 have greater than 1 copy per virion, suggesting, on average, constitutive incorporation. Our definition of a virion-incorporated protein would entail that it is present with at least one copy on average.

We added a clarification to the results (where discussing copy numbers):

*“53 viral proteins are present with more than one copy per virion.”*

And in the introduction:

*“In addition, previous studies only provided insight into the overall protein composition of herpesvirions, identifying between 46 and 82 viral proteins*[*^14–18^*](https://paperpile.com/c/sf2lJz/s9goN+dRpL+cv7s+dUz2+C3gJ)*”*

5. I would have liked to see a comment about the localisation of the binding sites identified on UL32 and what is already known about the structure of this protein. Particularly in regards to the date presented in Fig 3d.

The pp150 c-terminal region (AA303-1049) is disordered (i.e. without clearly defined tertiary structure and is also not visible in electron density). We mentioned this previously in the results:

*Considering that UL32 is anchored with its N-terminal domain at the nucleocapsid* [*^35^*](https://paperpile.com/c/sf2lJz/ELkY)*, we reasoned that the disordered C-terminal domain (AA 303-1049) may engage in interactions with the other layers*

Additionally, we now add this a second time to the results:

*Furthermore, UL32 and in particular its C-terminal disordered region engages in many interactions with host proteins, consistent with its scaffolding role for viral proteins.*

6. Fig 5d. Not clear why is this one-step growth but Fig 5a is a multi-step?

We included both a one step (Ext. Data Fig. 7a) and a multi-step growth curve (Fig. 5a) for comparing the PP-1-binding deficient mutant to wildtype.

Typically mutations in tegument proteins that play roles during entry (like UL32) have stronger (or sometimes even only) phenotypes during low MOI conditions. This is due to the fact that at high MOI, other, compensating mechanisms play a role. Using a high MOI may mask potential early effects. Since we did not know what to expect, we first used a low MOI condition (Fig. 5a) and then also tested high MOI (Ext. Data Fig 7a), exactly how we structured the manuscript.

The later functional experiments (Figure 5b,c) were performed under high MOI conditions, thus making it necessary to test the contribution of 14–3-3 to growth under high MOI conditions (Figure 5e).

Minor points

Lines 37-39. Not clear from this sentence what facilitates the onset of viral gene expression.

We rephrased to:

*“Virions contain the viral nucleic acid genome enclosed in a capsid protein shell. A number of co-packaged proteins facilitate the infection process and the onset of viral gene expression.”*

Line 72 Fig1A doesn’t strictly show crosslinking of lysines. Suggest changing text.

We transferred the reference to Fig.1a to the end of the paragraph. It reads:

*“Therefore, we isolated extracellular particles from infectious cell culture supernatants and cross-linked them with disuccinimidyl sulfoxide (DSSO), which connects lysines from proteins in close spatial proximity. After purification of the cross-linked virions using tartrate-glycerol density gradient centrifugation, protein extraction and tryptic digestion, the cross-linked peptides were identified by liquid chromatography mass spectrometry (LC-MS) (Fig. 1a).”*

Line 79. Seems to me that the second most abundant cross-link is with host proteins (Fig1C).

Nice catch! We corrected the text.

Lines 81-82 Text mentions that no cross-links found between intravirion and virion surface protein domains. Suggest adding this to the legend of Fig1D for clarity.

We have included the following sentence in the original version of the figure legend and feel that it is sufficiently clear:

*“No cross-links have been observed linking extra-virion domains of viral glycoproteins to their intra-virion domains or to intra-virion resident proteins.”*

Fig1E Needs further clarification in text. Are the links mapped on the structures the ones found in the study? Do these map to the histograms shown in Ext Fig 1a and b?

All this is correct. We clarified the text:

*“To analyze whether this constraint is met, we mapped our cross-links on structural models of the homotrimeric viral glycoprotein UL55 (also known as gB) in its pre- and post-fusion state (Fig. 1e, Extended Data Figs. 2a-b)."*

And Figure legend:

***Extended Data Fig.2. Data filtering and reproducibility. (a,b)*** *Histogram of distances between Cα atoms obtained from mapping cross-links* *onto the cryoEM structural models of homotrimeric UL55 in pre-* ***(a)*** *or post-fusion* ***(b)*** *conformations (pdb: 7kdp, 7kdd). See also Figure 1e for visualization of the cross-links on the structural models.*

Fig1F found it hard to discern the difference between the magenta and the orange colours used in the figure.

We changed the orange coloring in Figure 1.

Fig 2b Scale is different for each of the sub-clusters.

We now scaled each sub-cluster equally to allow better comparison.

Line 151 Add reference for known role/localisation of Ul83 in the outer tegument.

We added Reyda et al., J Virol, 2014 as a reference at the position in the text.

Line 188 Are these the only lysines on Ul86?

No, these are not the only lysines on UL86. We would like to refer the reviewer to Extended Data Figure 4c, which highlights the lysines on UL86 (blue lines on the UL86 bar).

Fig3A Ul32 rather than pp150 would be a clearer label.

Thanks, we corrected the label.

SuppTable 3 and 4 Some of the gene names have converted to dates

Thanks for carefully looking! We corrected the Tables.

Line 229 What about the protein copies not in the virion map (Fig3c). What are these?

This is the sum of all other (~2,000) proteins that were identified associated with purified particles. Almost all with very low copy numbers. We could not observe relevant functional enrichment among these.

Lines 250-252 Why not mark these proteins on Fig3e

We agree and marked OCRL and BICD2 on Figure 3e.

Fig4/ExtFig4 What are PLF cells (never mentioned in the methods), are these the same as human embryonic lung fibroblasts.

These are “primary lung fibroblasts” (PLFs) and refer to the same cells as “human embryonic lung fibroblasts” (HELFs). We replaced “PLFs” by “HELFs” as it appears more commonly used.

Fig4b Orange extremely hard to see here.

We increased saturation and decreased transparency of the orange.

Line 267 Clarification needed for how motifs were hypothesised. It is not clear from Fig4c.

See our comment to main point 3a, above.

Line 278 Which residues were converted to alanine?

We thank the reviewer for this comment and acknowledge that this was never mentioned in the text or Figures. We refer the reviewer to our new Supplementary Table 7, which contains all information about the mutated amino acids. In this specific case, the KTVAF (aa 1001-1005) amino acids were mutated to KTAAA and SILK (aa 969-972) were mutated to SALA.

Line 286 Significantly? P values not displayed?

The P-values are displayed in the Figure panel Ext. Data Figure 6d (top of the boxplot).

Line 307 20-fold fewer progeny at what time point? Need to clarify if this is total or extracellular virus used for the growth curves. Is recruitment of PP1 necessary for infectious viral progeny or for early steps post infection in the recipient cell?

We clarified that this is the maximum difference observed at 12 dpi and low MOI. This growth curve analysis captures replication across the entire viral life cycle including production of viral progeny and early steps post entry. We clarified in the Methods that immunotitrations for determining virus titers are based on extracellular virus.

Fig5c What is PRH1. Labelled on chart but not discussed

We removed the PRH1 label.

Line 384 Microtubuli? Is this correct.

We changed to microtubules as it appears more commonly used.

Line 711 Experimental design as in extended data as in fig 5c a typo? Potentially meant 5b?

Nice catch! We corrected this.

Reviewer #2 (Remarks to the Author):

In this study, Bogdanow et al. employed cross-linking mass spectrometry (XL-MS) to build an extensive virion-wide proximity map of both viral and host proteins in intact extracellular virions of human cytomegalovirus (HCMV). HCMV is a leading viral cause of birth abnormalities and a significant public health concern due to opportunistic infection in AIDS and immune-suppressed populations. Consistent with, and complementary to, a series of recent cryoEM publications of HCMV virion structures showing interactions among virion and host components, the XL-MS data enable perhaps the most substantial depiction of host and virus proteins and their protein-protein interactions (PPIs) within and across different virion layers (envelope, tegument and capsid) of HCMV. In particular, the authors show that tegument protein pUL32 recruits PP1 phosphatase and 14-3-3 proteins and its role in early and late steps during virion assembly. The data established that PP1 antagonizes 14-3-3 binding to

pUL32 and is required for efficient start of viral gene expression and production of viral progeny. Compared to previous MS publications, the current study represents a systematic characterization of the spatial coordination and interactions of virus and host proteins within virions. The paper is well written and illustrations nicely prepared. Overall, this paper provides global structural insights into the native configuration of virus and host protein interactions inside herpesvirus particles and has the potential to become a classic citation in the literature. I only have some minor issues that should be addressed.

We thank the reviewer for the positive feedback!

Minor issues:

1. Throughout the paper, gene names (such as UL32) and protein names are used indiscriminately. This is likely to cause confusion. All protein names should have a prefix of p, such as pUL32. Alternatively, well established common names (pp150 for pUL32 and MCP for UL86, for example) should be (and preferentially) used.

For the analysis of our dataset, we rely on fasta databases for the specific strain that was used. As there is no reference proteome for TB40-BAC4 available, we used the TB40-BAC4 database as provided by NCBI (see <https://www.ncbi.nlm.nih.gov/nuccore/157779983>). We agree with the reviewer that the naming of some of the viral proteins may be confusing. In order to avoid this potential confusion, we explicitly mentioned other common names for the respective genes upon first appearance in the manuscript.

However, the prefix “p” to indicate a protein is not as commonly used as suggested by the reviewer. Instead, several proteomics studies only use the gene names for their identified proteins. It is common practice in proteomic studies to mention the proteins with their corresponding gene name for human proteins (here, typically the HUGO Official gene symbol is used), and viral proteins (see e.g. Nobre et al., 2018, eLife). To account for this, we mentioned in the methods part that peptides were matched to proteins encoded by the respective genes and the gene name was then used for display in figures, tables and text.

2. Recent cryoEM has shown host RNA interaction with pp150 (Liu et al. Nat Comm). Could such interactions be detected by XL-MS? This should be commented at least in the Discussion.

In principle, there are cross-linking methods able to record protein-RNA interactions. See for example the work of the Leitner group (e.g. Dorn et al., Nat. Methods, 2017). The approach we performed exclusively captures protein-protein interactions. We clarified this in the discussion:

*“In addition, HCMV virions contain RNA species* [*^54,55^*](https://paperpile.com/c/sf2lJz/hSpR+iUcT)*, an aspect that our study did not cover.”*

Reviewer #3 (Remarks to the Author):

This study by Bogdanow et al. describes the analysis of herpesvirions by cross-linking mass spectrometry, a state-of-the-art technology that provides unique insight into spatial organization of complex proteomes. The authors identified over 9,000 crosslinks from which they could extract a high content network of protein-protein interactions (PPIs) in virions. By elegant calculation of PPI specificity values and hierarchical clustering, viral proteins were allocated to four different spatially resolved virion layers. Next to viral proteins, the authors report the layer-specific recruitment of 82 host proteins and they could distinguish specific from non-specifically recruited host proteins. Finally, the incorporation of host PP1 is shown to be important for viral replication by stimulating (early) viral gene expression and by antagonizing (late) incorporation of 14-3-3 proteins.

The study is well performed and the data is of high quality. The work is pioneering virion architecture mapping by XL-MS and therefore of high impact.

We thank the reviewer for the positive feedback!

I do have a few points that need to be addressed though:

1. Do I understand it correctly that virions were first concentrated and cross-linked, and only purified afterwards? How did you control potential contamination from extracellular proteins, vesicles or debris that might become cross-linked to the particles? Did you check for instance the quality of the preparations by EM or any other method? At least potential limitations of the approach should be discussed.

Yes, the reviewer is correct and also has a good point! In the standard viral purification protocol we adapted from a previous publication (ref. # 63 in our manuscript), we and others observed that purified particles are damaged due to purification as evidenced by membrane blebbing (ref #16, Reyda et al., and ref #25, Si et al.). For this reason we chose to cross-link the particles at an upstream stage (i.e. before purification), as we reasoned that this would better capture their native morphologies at the point of cross-linking.

We expect that HCMV particles cross-linked to vesicles or large protein assemblies would not run at the same density as single particles. To assess this expectation, we imaged HCMV particles in dependence of cross-linking and purification (see below, Figure R15) using negative staining and transmission electron microscopy (TEM). We observed that the average diameter of particles (~190 nm) was not altered by performing cross-linking prior to purification. This argues against major contaminants due to cross-linking. The effect of the cross-linking protocol is rather that it leads to a slightly higher percentage of disintegrated particles (smaller than 160 nm diameters) compared to non-cross-linked samples. Importantly, these, most likely disrupted particles, can be removed by the purification protocol, enriching virions in the expected size range.

We added the data to the revised version of the manuscript and refer to it in the results section.

*“We evaluated the quality of the particle preparations in dependence of performing cross-linking and/or gradient purifications by negative staining electron microscopy (EM) (Extended Data Fig. 1).”*

**Figure R15. Estimation of sample purity from cross-linked and non-cross-linked HCMV particles.**

**(a)** Exemplary micrographs of negatively stained HCMV particles. Purification using glycerol tartrate gradients and cross-linking treatment as indicated. Scale bar represents 1000 nm. Manually selected particles are indicated by yellow circles. **(b,c)** Distribution of diameters of manually picked particles of the non-cross-linked (b) or cross-linked (c) samples. Purification led to a more homogenous population of particle diameters. As previously noted [^25^](https://paperpile.com/c/sf2lJz/9ccz), we also find that the purification leads to membrane blebs, thus representing non-native particle morphologies. **(d,e)** Two cut-offs were defined to categorize unusually small (<160 nm) and large particles (>220 nm). These correspond to the median of diameters from the purified sample +/- two times the standard deviation. The fraction of particles with diameters that fall below 160 nm, exceed 220 nm or fall in between are indicated for the non-cross-linked (d) or cross-linked (e) sample types. Purification results in more homogeneous particle diameters, enriching virions in the expected size range. Thus, our protocol captures the native configuration of the particles at relatively high sample purity. At least 367 particles were counted per condition. These data were added to Extended Data Fig. 1.

2. Validation of the 4-layered virion architecture by an orthogonal approach or additional experiments would significantly strengthen the conclusions. I understand this might be difficult, but as a pioneering study in this area this might be worthwhile considering. The authors could for instance analyze mutant virions that are deficient in any of the main layer scaffolds (such as UL86, UL83 or UL100) and show that the architecture is disrupted. Again, if such experiments are not feasible, at least possible future validation and/or follow-up studies should be discussed.

This is a valid point that we were also thinking about. Deletion of the scaffolding genes UL86, UL32 and UL100 is lethal for the virus underscoring the importance of these scaffolds for proper architecture and efficient virion production. The deletion of UL83 (also known as pp65, annotated as outer tegument scaffold) is however feasible and the composition of viral particles devoid of UL83 were previously analyzed by proteomics (reference 22) and western blotting (reference 34). We referred to the phenotype of this mutant in the following paragraph in the results:

*“The second distinct subcluster represents the outer, non-membrane bound tegument. Its composition fits previous observations showing that the UL83 (also known as pp65) protein is important for incorporating UL69, UL97 and UL25 into mature particles* [*^16,35^*](https://paperpile.com/c/sf2lJz/syq9+cv7s) *“*

In order to provide additional evidence of the scaffolding role of the UL32 c-terminal disordered domain, we created a mutant with the C-terminal domain deleted (AA303-AA1049). Unfortunately, this mutant was not viable, which prohibited further experiments but is consistent with the scaffolding role of UL32 C-terminal domain. Experiments are ongoing with mutant viruses, having C-terminal truncations of pp150 in different lengths. However, including experiments with these mutants is out of scope for this manuscript and likely fits better to another publication.

While these issues prevent experimental disruption of virion architecture, we would like to point out that we assessed the plausibility of our findings by an *in silico* approach, creating a coarse-grained model of the particle (see Figure 6 and Extended Data Fig. 10). This model recapitulates the major role for UL32 in tegument organization as established in this manuscript (e.g. the domain-dependent localizations of UL32 within the virion).

3. The authors conclude that HCMV recruits PP1 to limit antiviral or perturbing effects of 14-3-3 proteins (discussion, line 374), but their findings should also be discussed in light of previous pro- or antiviral functions assigned to PP1 and 14-3-3 proteins.

We thank the reviewer for this comment. Due to manuscript length limitations by the journal we only had enough space to discuss the most important aspects of the manuscript. We agree that adding more context would be helpful to the readers. We added the following sentences to the revised version of the manuscript. The paragraph reads as follows:

*“PP-1 is a target that is exploited by various viral proteins, such as HSV1 ICP34.5* [*^48^*](https://paperpile.com/c/sf2lJz/9bnZ)*, HSV-1 pUL21* [*^49^*](https://paperpile.com/c/sf2lJz/J49F)*, Measles/Nipah virus V protein* [*^50^*](https://paperpile.com/c/sf2lJz/vbiU) *and HIV Tat* [*^51^*](https://paperpile.com/c/sf2lJz/jmPz)*. This regulates phospho-sensitive processes such as translation* [*^48^*](https://paperpile.com/c/sf2lJz/9bnZ)*, transcription* [*^51^*](https://paperpile.com/c/sf2lJz/jmPz) *and RNA-sensing* [*^50^*](https://paperpile.com/c/sf2lJz/vbiU)*. Recruitment of PP-1 to UL32 is important for high-titer replication of HCMV (Figure 5a) , consistent with previous inhibitor experiments targeting the substrate recruitment site of PP-1* [*^52^*](https://paperpile.com/c/sf2lJz/zi6j)*. We show that this interaction modulates the phosphorylation status of UL32 and positively regulates the onset of viral gene expression (Figure 5b,d, Ext. Data Fig. 6d).”*

4. Some concepts and analytical steps should be better explained in the main text:

- line 96: please explain the rationale behind the further filtering criteria.

We thank the referee for this suggestion and agree that this can be explained better. We added a few sentences and rephrased the corresponding section in the Methods section:

*“In order to supply a XL-MS PPI-map with high density of relevant biological information, we applied further filtering criteria. For this filtering, it was first checked whether cross-links were identified in both replicates independently at 1% FDR. We required that cross-links from abundant virion proteins (> 10 copies) be identified in both replicates. When a cross-link involved a lower abundant protein (that is, < 10 copies) it was required that the cross-link is only identified in one of the replicates. The rationale is that this balances cross-link confidence for highly abundant proteins with sensitivity for low abundant proteins. Second, PPIs were only reported when there were two unique cross-links identified, giving a higher confidence set of PPIs. In order to retain only host proteins incorporated into the particle, host proteins were removed that did not directly link to viral proteins. The filtered set of cross-links is available in Supplementary Table 2. The list of the corresponding PPIs together with evidence from existing data is available in Supplementary Table 3”*

- line 187: better explain how host proteins were assigned to the different virion layers. Also, provide a more extensive legend for Fig 3a. Where can I see the viral marker proteins in this figure?

We rephrased the corresponding text as:

*“To determine their location, we first counted the cross-links that individual host proteins had to viral proteins of the different layers, as established in Figure 2a. To normalize for differences in the absolute number of cross-links these layer-specific link counts were then z-scored. Clustering these data resolved host protein localization to the individual layers.”*

In addition, we rephrased the legend of Figure 3a

***“(a)*** *Heatmap depiction host protein localization across virion layers. Cross-link counts of host proteins to viral proteins of the respective layers (NC: nucleocapsid, IT: inner tegument (nucleocapsid-proximal tegument), OT: outer tegument, M: membrane, including viral transmembrane proteins and membrane-proximal tegument) were z-scored and hierarchically clustered using euclidean distance.”*

The reviewer is also right in not seeing the viral marker proteins, as they are not included. Including them would require referencing individual viral proteins to themselves.

- line 325: please briefly explain that the AP-MS experiments were performed on cells infected with the different virus variants (and not for instance on cells transfected with UL-32 variants).

We changed the respective text to:

*“To this end, we performed two interaction proteomics experiments from virus infected cells: First, comparing the interactors of WT-UL32 to the SILK/RVxFmut-UL32 (Extended Data Fig. 7a-b) and second, comparing the interactors of WT-UL32 under treatment with phosphatase inhibitor calyculin A to a control (Extended Data Fig. 7c-d).”*

5. Please provide more details on the XL proteomics sample preparation (page 24-25), ideally by adding a step-by-step protocol as a Supplementary Methods file. At least following information should be added:

- the amount of virions and cells the analyses were started from, and the amount of extracted/digested proteins

- clearly indicate the number of experiments that were performed and the number of replicates/experiments

- provide origin of trypsin and LysC enzymes and a brief description of the C8 and C18 purifications

- provide full details on the strong cation exchange fractionation for cross-linked peptides, (e.g. what is the mobile phase?) or refer to previous published work

We thank the reviewer for this comment. We now include an extensive step-by-step protocol in the Supplementary Information addressing these points.

6. Similarly, provide more details on the AP-MS experiments, I am missing for instance information on the starting amounts of cells and , number of replicates, etc. Line 490: I assume proteins were eluted by adding 200 microliter of 8M guanidine hydrochloride?

We now include specifics about the starting material for AP-MS. The number of replicates is depicted in the figure legends throughout the manuscript. We now added this as well to the methods:

*“HELFs (human embryonic lung fibroblasts) were infected with HCMV-UL32-GFP (phosphatase-activity dependent interactome, GFP-AP), HCMV-UL32-HA or HCMV-UL32-SILK/RVxFmut-HA (PP1-binding dependent interactome, HA-AP) at an MOI of 5 IU/cell. Experiments were performed in n=3 replicates, with one confluent 15-cm dish as starting material per replicate and experiment.”*

Concerning the concentration of guanidine hydrochloride: This is correct! We added the 8 M concentration to the text.

7. A clear overview of all viral mutants and their sequences that were used in the study should be provided. For instance, I could not find the exact positions of the phosphosite mutants in Fig. 4. Also, what are the critical amino acid residues that were mutated in the SILK/RFxFmut variant? How were these mutant viruses created?

We now provide the readers now in Supplementary Table 7 with a full list of amino acid sequences mutated in this study. In the case of PP-1 binding mutants, the RVxF motif (KTVAF, aa 1001-1005 in UL32) was mutated to RAxA (KTAAA) and the SILK motif (SILK, aa 969-972 in UL32) was mutated to SALA (SALA). As mentioned in the Methods section, all virus mutants were created by a traceless BAC mutagenesis protocol established by Tischer et al. See Supplementary Table 7 for nucleotide sequences of the mutagenesis primers.

8. The authors submitted their raw data files to the PRIDE database, but I am missing a clear overview of which file(s) belong to which experiment(s). Please provide such overview, for instance as a Supplementary Table, so readers can re-use the files in a meaningful way.

We apologize for this negligence and provide an additional Supplementary Table 8 containing an overview of proteomic experiments and their corresponding files.

| **Decision Letter, first revision:** | | | |
| --- | --- | --- | --- |
|  | **Message:** | Our ref: NMICROBIOL-22051057A  27th April 2023  Dear Dr. Liu,  Thank you for submitting your revised manuscript "Spatial, Quantitative and Functional Deconstruction of Virus and Host Protein Interactions Inside Intact Cytomegalovirus Particles" (NMICROBIOL-22051057A). It has now been seen by the original referees and their comments are below. The reviewers find that the paper has improved in revision, and therefore we'll be happy in principle to publish it in Nature Microbiology, pending minor revisions to satisfy the referees' final requests and to comply with our editorial and formatting guidelines.  If the current version of your manuscript is in a PDF format, please email us a copy of the file in an editable format (Microsoft Word or LaTex)-- we can not proceed with PDFs at this stage.  We are now performing detailed checks on your paper and will send you a checklist detailing our editorial and formatting requirements several weeks. Please do not upload the final materials and make any revisions until you receive this additional information from us.  Thank you again for your interest in Nature Microbiology Please do not hesitate to contact me if you have any questions.  [redacted]    Reviewer #1 (Remarks to the Author):  The authors have extensively revised their manuscript and included really valuable new data, particularly the first-ever coarse-grained structural model of a herpes virion. They should be congratulated for all of their hard work with this. As such, the manuscript is very well suited for publication in Nature Microbiology without further modification, and will form an extremely valuable addition to the field.   Reviewer #2 (Remarks to the Author):  The authors have done a thorough and careful revision in responding to the reviewers’ comments. My concerns have been addressed adequately.   Reviewer #3 (Remarks to the Author):  I appreciate the author’s efforts in addressing my comments. The manuscript has significantly improved and the coarse-grained model is a strong addition to the paper. Apart from the final small comment below, I have no further questions for the authors.  The legend of Extended Data Figure 1 states that “Manually selected particles are indicated by yellow circles”. I don’t see any yellow circles in the figure. Please adapt text and/or figure. |  |

**Author Rebuttal, first revision:**

Point-by-point response

Reviewer #1:

Remarks to the Author:

The authors have extensively revised their manuscript and included really valuable new data, particularly the first-ever coarse-grained structural model of a herpes virion. They should be congratulated for all of their hard work with this. As such, the manuscript is very well suited for publication in Nature Microbiology without further modification, and will form an extremely valuable addition to the field.

*We thank the reviewer for his/her positive evaluation!*

Reviewer #2:

Remarks to the Author:

The authors have done a thorough and careful revision in responding to the reviewers’ comments. My concerns have been addressed adequately.

*We thank the reviewer for his/her valuable assessment!*

Reviewer #3:

Remarks to the Author:

I appreciate the author’s efforts in addressing my comments. The manuscript has significantly improved and the coarse-grained model is a strong addition to the paper. Apart from the final small comment below, I have no further questions for the authors.

*We thank the reviewer for evaluating our manuscript!*

The legend of Extended Data Figure 1 states that “Manually selected particles are indicated by yellow circles”. I don’t see any yellow circles in the figure. Please adapt text and/or figure.

*We agree that the circles are hardly visible. We therefore overlaid them with yellow circles/ellipses having an increased stroke width.*

**Final Decision Letter:**

| **Message:** | 20th June 2023  Dear Fan,  I am pleased to accept your Article "Spatially resolved protein map of intact human cytomegalovirus particles" for publication in Nature Microbiology. Thank you for having chosen to submit your work to us and many congratulations.  Over the next few weeks, your paper will be copyedited to ensure that it conforms to Nature Microbiology style. We look particularly carefully at the titles of all papers to ensure that they are relatively brief and understandable.  Once your paper is typeset, you will receive an email with a link to choose the appropriate publishing options for your paper and our Author Services team will be in touch regarding any additional information that may be required. Once your paper has been scheduled for online publication, the Nature press office will be in touch to confirm the details.  After the grant of rights is completed, you will receive a link to your electronic proof via email with a request to make any corrections within 48 hours. If, when you receive your proof, you cannot meet this deadline, please inform us at rjsproduction@springernature.com immediately. You will not receive your proofs until the publishing agreement has been received through our system  Due to the importance of these deadlines, we ask you please us know now whether you will be difficult to contact over the next month. If this is the case, we ask you provide us with the contact information (email, phone and fax) of someone who will be able to check the proofs on your behalf, and who will be available to address any last-minute problems.  Acceptance of your manuscript is conditional on all authors' agreement with our publication policies (see https://www.nature.com/nmicrobiol/editorial-policies). In particular your manuscript must not be published elsewhere and there must be no announcement of the work to any media outlet until the publication date (the day on which it is uploaded onto our website).  Please note that <i>Nature Microbiology</i> is a Transformative Journal (TJ). Authors may publish their research with us through the traditional subscription access route or make their paper immediately open access through payment of an article-processing charge (APC). Authors will not be required to make a final decision about access to their article until it has been accepted. <a href="https://www.springernature.com/gp/open-research/transformative-journals"> Find out more about Transformative Journals</a>  **Authors may need to take specific actions to achieve <a href="https://www.springernature.com/gp/open-research/funding/policy-compliance-faqs"> compliance</a> with funder and institutional open access mandates.** If your research is supported by a funder that requires immediate open access (e.g. according to <a href="https://www.springernature.com/gp/open-research/plan-s-compliance">Plan S principles</a>) then you should select the gold OA route, and we will direct you to the compliant route where possible. For authors selecting the subscription publication route, the journal’s standard licensing terms will need to be accepted, including <a href="https://www.nature.com/nature-portfolio/editorial-policies/self-archiving-and-license-to-publish">self-archiving policies</a>. Those licensing terms will supersede any other terms that the author or any third party may assert apply to any version of the manuscript.   If you have any questions about our publishing options, costs, Open Access requirements, or our legal forms, please contact ASJournals@springernature.com  An online order form for reprints of your paper is available at <a href="https://www.nature.com/reprints/author-reprints.html">https://www.nature.com/reprints/author-reprints.html</a>. All co-authors, authors' institutions and authors' funding agencies can order reprints using the form appropriate to their geographical region.  We welcome the submission of potential cover material (including a short caption of around 40 words) related to your manuscript; suggestions should be sent to Nature Microbiology as electronic files (the image should be 300 dpi at 210 x 297 mm in either TIFF or JPEG format). Please note that such pictures should be selected more for their aesthetic appeal than for their scientific content, and that colour images work better than black and white or grayscale images. Please do not try to design a cover with the Nature Microbiology logo etc., and please do not submit composites of images related to your work. I am sure you will understand that we cannot make any promise as to whether any of your suggestions might be selected for the cover of the journal.  You can now use a single sign-on for all your accounts, view the status of all your manuscript submissions and reviews, access usage statistics for your published articles and download a record of your refereeing activity for the Nature journals.  To assist our authors in disseminating their research to the broader community, our SharedIt initiative provides you with a unique shareable link that will allow anyone (with or without a subscription) to read the published article. Recipients of the link with a subscription will also be able to download and print the PDF.   As soon as your article is published, you will receive an automated email with your shareable link.  Congrats again to you and your co-authors! I'm looking forward seeing your paper published!   With kind regards,  [redacted]    P.S. Click on the following link if you would like to recommend Nature Microbiology to your librarian http://www.nature.com/subscriptions/recommend.html#forms   ** Visit the Springer Nature Editorial and Publishing website at <a href="http://editorial-jobs.springernature.com?utm_source=ejP_NMicro_email&utm_medium=ejP_NMicro_email&utm_campaign=ejp_NMicro">www.springernature.com/editorial-and-publishing-jobs</a> for more information about our career opportunities. If you have any questions please click <a href="mailto:editorial.publishing.jobs@springernature.com">here</a>.** |
| --- | --- |
